# Frequency-oriented adaptive real-time object detector for cluttered traffic scenes

**DOI:** 10.1038/s41467-026-76346-1

**Published:** 2026-09-14

**Authors:** Ziqi Li, Tao Gao, Shutao Li, Ting Chen, Yisheng An, Yuanbo Wen, Tao Lei

**Affiliations:** 1 School of Information Engineering, Chang’an University, Xi’an, China; 2 School of Data Science and Artificial Intelligence, Chang’an University, Xi’an, China; 3 School of Electronic Information and Artificial Intelligence, Shaanxi University of Science and Technology, Xi’an, China

**Keywords:** Computer science, Technology

## Abstract

Given the growing demand for traffic object detection in autonomous driving, achieving both efficiency and accuracy on in-vehicle platforms remains challenging. To address this issue, we propose a Frequency-Oriented Adaptive Detector for vehicle-mounted intelligent traffic object detection. It integrates high- and low-frequency information to enhance the texture and semantic representations of multi-scale objects in complex traffic scenarios while maintaining low computational overhead. Specifically, we introduce Frequency Dynamic Convolution to construct a lightweight backbone with frequency-domain adaptive dilated receptive fields and balanced effective bandwidth. The adaptive kernel decomposes convolutional weights into high- and low-frequency components, which are selectively recalibrated to balance frequency responses in feature maps. Moreover, we propose an Adaptive Frequency-Oriented Fusion framework to reorganize high- and low-frequency features across scales. The framework balances object details, fine boundaries, and deep semantic features, thereby reducing feature inconsistencies during multi-scale fusion. Extensive experiments demonstrate that our Frequency-Oriented Adaptive Detector outperforms state-of-the-art detectors. With only 8.77 million parameters, it achieves mAP values of 90.9%, 53.7%, 50.2%, and 53.1% on KITTI, BDD100K, Cityscapes, and Waymo datasets respectively.

## Abstract (web summary)

This study presents an efficient AI detector for intelligent vehicles called Frequency-oriented Adaptive Detector. By adaptively using high- and low-frequency visual information, it improves object detection in complex traffic scenes while keeping computation low.

## Introduction

Vision-based traffic scene perception has emerged as a key research frontier in intelligent transportation systems, enabling the detection, recognition, and tracking of diverse road participants. Accurate object detection forms the foundation of reliable scene understanding, serving as a critical component for downstream tasks such as autonomous navigation and traffic management. However, vehicle-mounted traffic object detection (TOD) remains a challenging task due to several inherent difficulties, including scale variation induced by perspective changes, frequent occlusions, diverse lighting and weather conditions, and the limited performance of lightweight detection networks when handling low-quality or small-scale objects. Representative examples of these complexities are illustrated in Supplementary Fig. 1, drawn from the KITTI^1^, BDD100K^2^, Cityscapes^3^ and Waymo^4^ datasets.

Deep learning-based object detection algorithms can be broadly categorized into two main paradigms: two-stage and one-stage detectors. Representative two-stage paradigms, such as Faster R-CNN^5^ decouple object detection into proposal generation and subsequent per-region refinement, striking a favorable accuracy-efficiency trade-off but typically incurring considerable computational cost. In contrast, one-stage detectors such as SSD^6^, YOLO^7–12^, and RetinaNet^13^ formulate object detection as a unified dense prediction task, directly regressing class probabilities and bounding box coordinates in a single feed-forward pass, thereby achieving a more favorable balance between accuracy and inference efficiency. Nonetheless, deploying these models on embedded automotive platforms remains non-trivial due to stringent constraints on GPU capacity and memory bandwidth, which substantially hinder the practicality of computation-intensive detectors in real-world scenarios.

To achieve a favorable trade-off between detection performance and computational efficiency on vehicle-mounted platforms, extensive efforts have focused on model compression and architectural optimization. Regarding feature extraction, recent studies have underscored the importance of a large receptive field for scene understanding^14,15^, although enlarging it inevitably incurs additional computational cost. To alleviate this trade-off, content-adaptive mechanisms^16–23^ have been proposed to dynamically adjust convolutional operations based on input-dependent variations. Vision Transformers^15,24–26^ further advance this paradigm by employing self-attention to capture long-range dependencies ^27–31^, yet their substantial computational overhead restricts their applicability on embedded systems. Alternatively, dilated convolutions expand the receptive field by inserting spatial gaps between kernel elements with a predefined dilation rate *D*, enabling broader contextual perception at relatively low computational cost-but often at the expense of sensitivity to high-frequency details^32^. Meanwhile, deformable convolutions^16,33,34^ introduce learnable spatial offsets of *K* × *K* × 2 to adaptively capture geometric transformations across diverse visual conditions. However, in object detection-where precise spatial localization is essential-preserving feature consistency under such adaptive sampling remains a significant challenge.

Notably, in autonomous driving scenarios, environmental noise predominantly manifests as low-frequency interference in the frequency domain^35–37^, whereas the structural and textural details of traffic objects are primarily concentrated in high-frequency components. Exploiting this spectral distinction, frequency-domain feature extraction enables the suppression of background noise while enhancing the representation of salient objects. Frequency analysis, a long-standing foundation of signal processing^38,39^, has recently been incorporated into deep learning frameworks to model non-local dependencies^40–44^ and to improve domain generalization^45^. According to the scaling property of the Fourier Transform^46,47^, increasing the dilation rate from 1 to *D* proportionally narrows the frequency response bandwidth by a factor of $\frac{1}{D}$, thereby reducing the model’s sensitivity to high-frequency details. This insight motivates the incorporation of frequency-domain principles into convolutional network design to enhance multi-frequency feature representation^48^. From a spectral perspective, feature alignment and boundary precision are governed by distinct frequency components: low-frequency structures encode semantic consistency, while high-frequency components capture boundary details. However, multi-scale operations introduce frequency-dependent distortions that disrupt this balance, leading to feature inconsistency and boundary displacement. Consequently, the underlying challenge is a spectral imbalance, for which frequency-domain modeling provides a natural and principled solution. Additional background on frequency-domain learning and related detection methods is provided in the Supplementary.

Building upon these insights, we propose Frequency Dynamic Convolution (FDC), a novel convolutional operator that aims to extract discriminative object features from frequency-domain noise in complex traffic scenes, eliminate spatial deviations, and enhance feature consistency for position-sensitive regression. Specifically, FDC refines conventional dilated convolution through spectral analysis by introducing a unified, frequency-guided dilation formulation that mitigates redundant coordinate sampling inherent in standard convolutions. This frequency-aware dilation strategy alleviates the heuristic and non-interpretable nature of empirically defined dilation parameters. By dynamically modulating the dilation rate in a spatially adaptive manner, FDC optimizes kernel bandwidth utilization and learns frequency-balancing functions that expand the receptive field while preserving critical high-frequency details, thereby achieving a principled equilibrium between effective bandwidth and contextual coverage.

**Fig. 1:**
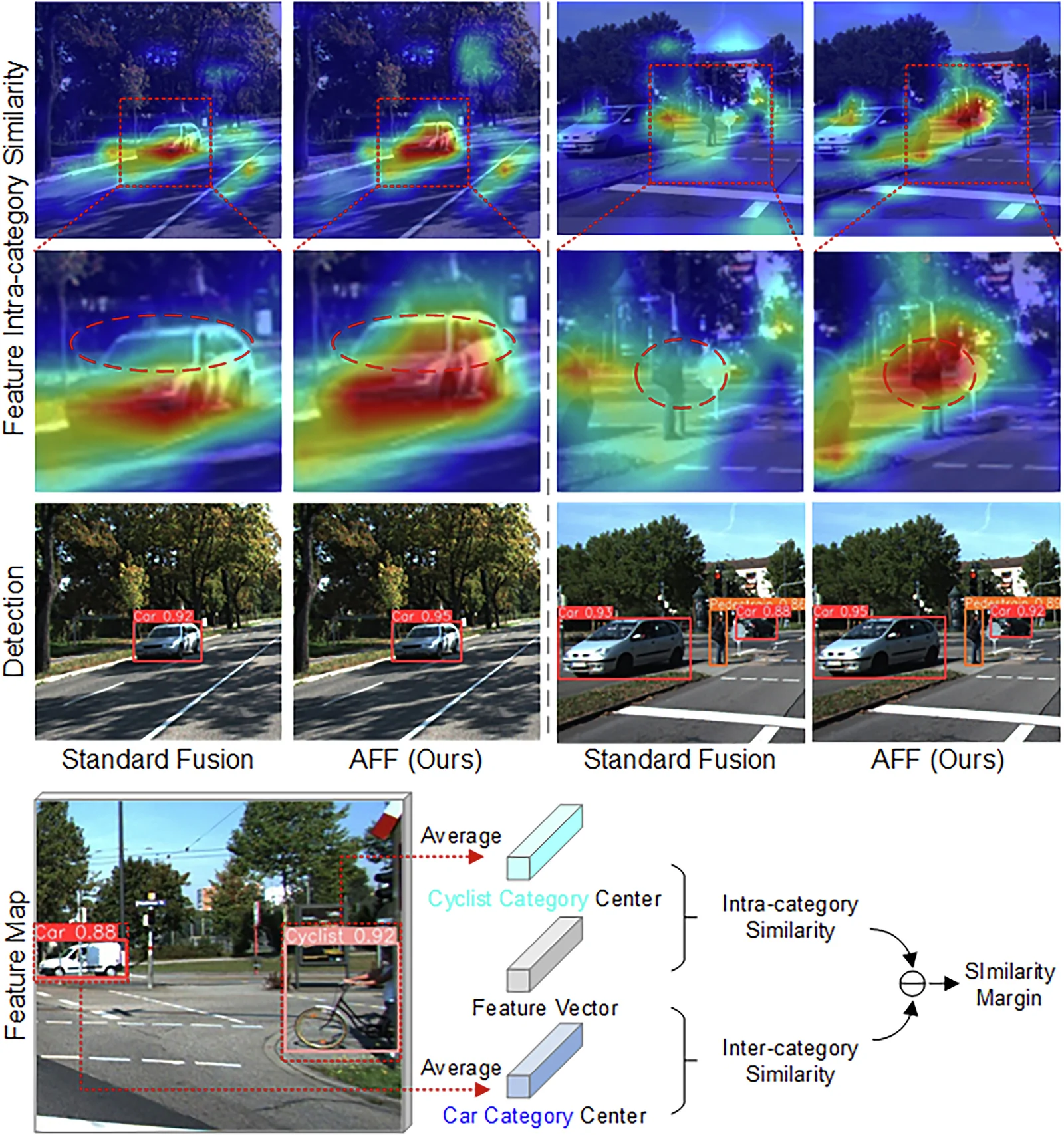
Illustration of intra-category similarity, inter-category similarity, and similarity margin. We visualize intra-category similarity and detection in the above figure. Brighter colors indicate higher intra-category similarity for car (left) and cyclist (right). Standard fusion shows low intra-category similarity and blurred boundaries, reflecting inconsistent high-frequency features and boundary displacement. AFF yields more coherent features and sharper edges, leading to more consistent and precise predictions. Images from the KITTI Vision Benchmark Suite are used with permission and made available under the CC BY-NC-SA 3.0 licence (https://creativecommons.org/licenses/by-nc-sa/3.0/).

Moreover, autonomous driving scenes pose substantial challenges, including background noise, illumination variation, motion blur, and multi-scale traffic objects induced by perspective effects, thereby necessitating the extraction of features with strong category semantics and precise spatial boundaries. To address these challenges, feature fusion has achieved promising performance in integrating high-level semantic features with low-level high-resolution details^49,50^, and has therefore been widely adopted in various architectures^51–54^. In general, high-level features provide rich categorical context, whereas low-level features preserve fine-grained spatial details. However, upsampling coarse features via nearest-neighbor or bilinear interpolation and merging them with high-resolution features often leads to intra-category inconsistency and boundary displacement^48^. The former arises from variations within the same object, such as differences in texture and brightness between a vehicle’s wheel and window^55^, while the latter is caused by excessive smoothing introduced during interpolation^56,57^. Conventional fusion methods^53^ are unable to effectively address these issues and may even exacerbate them by diffusing a single feature across multiple pixels. To quantify these phenomena, feature similarity analysis is employed, as illustrated in Fig. 1. Intra-category inconsistency is evaluated through intra-category similarity, which measures the similarity between each feature vector and its corresponding category center^58^. Likewise, inter-category similarity is computed to assess the similarity margin between different classes. Boundary displacement is characterized by low intra-category similarity and small similarity margins in boundary regions. Since classification scores depend on the similarity between category-aware weights and feature representations^59^, features with reduced intra-category similarity and margin ultimately yield lower classification confidence, leading to potential misclassifications.

Therefore, rapid variations in feature values within traffic objects, corresponding to disturbed high-frequency components, lead to low intra-category similarity and feature inconsistency^60^. Feature inconsistency and boundary degradation arise from the imbalance of frequency components across spatial locations, where insufficient high-frequency information further results in imprecise boundary localization. This highlights the necessity of frequency-aware modeling for preserving feature consistency and boundary precision. Accordingly, we propose an adaptive frequency-oriented fusion (AFF) framework to regulate the allocation of frequency components during multi-scale feature integration. AFF consists of four key components: Low-pass Adaptive Filter (LAF) generator, offset generator, High-pass Adaptive Filter (HAF) generator, and Cross-scale Contribution Allocator (CCA).

Specifically, the LAF applies a low-pass filter to smooth and upsample coarse high-level features, reducing disparities between pixel values and mitigating feature inconsistency. However, when employed independently, it is insufficient to correct large-area inconsistencies or refine boundary details. To address this limitation, we introduce an offset generator. The offset generator first calculates local similarity and then predicts offsets toward regions of high similarity for resampling, effectively replacing features with low intra-category similarity. It rectifies inconsistent features across both large regions and narrow boundary areas. Restoring fine boundary details lost during downsampling of low-level features into high-level representations remains challenging. According to the Nyquist-Shannon sampling theorem^61,62^, frequencies exceeding the Nyquist threshold, defined as half the sampling rate, are irretrievably lost. To address this limitation, we introduce the HAF generator, which applies spatially adaptive high-pass filters to low-level features, extracting intricate boundary details and enhancing high-frequency energy beyond the Nyquist limit. During bottom-up feature integration, the CCA progressively fuses high-level features with low-level features from non-adjacent scales, reducing the semantic gap between disparate scales. Through the coordinated operation of these four components, AFF reconstructs fused features that preserve consistent category information and well-defined object boundaries in complex traffic scenarios.

In this work, we present a frequency-oriented adaptive detector (FAD) for object detection in complex traffic scenes. FAD integrates frequency dynamic convolution (FDC) for frequency-guided feature extraction and adaptive frequency-oriented fusion (AFF) for multi-scale feature integration. FDC adjusts receptive fields through adaptive dilation rates, adaptive kernels, and frequency selection, enabling frequency-aware feature extraction across scales. AFF combines low-pass filtering, offset-based feature alignment, high-pass filtering, and cross-scale contribution allocation to integrate adjacent and non-adjacent scale features. By exploiting complementary low- and high-frequency cues, FAD improves the representation of object textures, boundaries, and semantic structures. Experiments on KITTI, BDD100K, Cityscapes and Waymo 2D show that FAD achieves competitive detection performance with fewer parameters and improved computational efficiency compared with representative methods.

## Results

### Implementation specifications

The network is trained under the following configuration. Mosaic data augmentation is applied with a stochastic probability of 0.5. Training is performed using the Stochastic Gradient Descent (SGD) optimizer with a momentum of 0.9, an initial learning rate of 0.01, and cosine annealing to decay the learning rate to 0.0001. Input images are resized to 640 × 640 pixels and drawn from four datasets, with a batch size of 8. All experiments are conducted in the PyTorch framework on a Windows workstation equipped with an RTX 4070 Ti 12-GB GPU and 32-GB RAM. To further evaluate deployment feasibility, inference is additionally performed on an in-vehicle computing platform RK3588, verifying the deployment suitability and runtime efficiency of the model on an automotive-grade platform.

### Datasets

#### KITTI dataset

^1^ comprises 7481 training images and 7518 test images, with a total of 51867 objects in the training set, of which cars represent 28742. The main challenge in KITTI car detection lies in the abundance of small-sized cars (height < 40 pixels) and occluded instances. Due to the unavailability of ground truth annotations for the KITTI test set, divided 3682 images and 3799 images into training and validation, respectively, for our framework analysis. In the KITTI dataset, performance evaluation was conducted across three difficulty levels: easy, moderate, and hard. The criteria for categorizing these levels and accounting for occlusion scenarios are detailed in Supplementary Table 1.

#### BDD100K dataset

^2^ comprises 100,000 video sequences captured by a car camera, each with a resolution of 1280 × 720 pixels. These sequences cover various times of day, driving scenarios, and weather conditions, and include 10 classes: car, bus, person, bike, truck, motor, train, rider, traffic sign, and traffic light. The dataset contains images with diverse illumination conditions and object sizes. The dataset is partitioned into training, validation, and test sets in a ratio of 7:1:2. Our model is trained using the training and validation data and evaluated on the test set to assess its performance.

#### Cityscapes dataset

^3^ is a widely used benchmark for urban object detection, consisting of high-resolution street-view images captured in 50 European cities under diverse environmental conditions. It features densely populated urban scenes with complex backgrounds, frequent occlusions, and a wide range of object scales. Following the standard split, we use 2975 finely annotated images for training and 500 for validation. These characteristics make Cityscapes suitable for evaluating object detection models in realistic and challenging urban environments.

#### Waymo 2D dataset

^4^ provides camera-based annotations for vehicles, pedestrians, and cyclists. Bounding boxes tightly enclose only the visible regions of objects without estimating occluded parts. Each object is consistently tracked across frames with occlusion states marked. Vehicles include cars and motorcycles but exclude trains and trams; pedestrians cover walking persons and those on small mobility devices; cyclists include both the rider and bicycle. This dataset offers precise, visibility-consistent labels for 2D perception and tracking tasks in autonomous driving.

Representative samples from the KITTI, BDD100K, Cityscapes, and Waymo datasets are provided in Supplementary Fig. 2, illustrating diverse traffic scenes and environmental conditions.

### Ablation study and analysis

We perform the ablation experiment on the KITTI benchmark to demonstrate the effectiveness of FDC, AFF and FAD. Table 1 shows the results of ablation experiments.

**Table 1:** Ablation experiment on KITTI dataset

Method	mAP@50	Car	Cyclist	Pedestrian	Params (M)	FLOPs (G)
Baseline	87.7	96.4	87.5	79.1	8.07	24.8
+ FDC	89.3	97.1	89.0	81.7	8.15	32.6
+ AFF	90.2	97.4	90.3	83.0	6.99	33.2
+ FDC + AFF (FAD)	**90.9**	**97.4**	**91.8**	**83.5**	8.77	39.7

#### Baseline

Initially, we utilize DarkNet53 as the backbone network and PANet to extract multiscale feature maps of autonomous driving images. The decoupling head is employed for object detection. In all experiments, uniform hyperparameters are maintained to ensure fairness in comparison. Finally, the baseline achieves 87.7% mAP on KITTI.

#### Effect of FDC

FDC is devised for frequency-oriented adaptive receptive field expansion, enhancing feature extraction capability for low-quality objects. Integrated with FDC, our model achieves a mAP of 89.3%, indicating a 1.6% improvement. A notable 2.6% improvement was observed in the pedestrian category, and a substantial 2.5% improvement in the cyclist category, both prone to occlusion and forming small pixel objects in the distance. In addition, the model’s parameters (Params) saw a minor rise from 8.07 M to 8.15 M, while floating point operations (FLOPs) increased slightly from 24.8G to 32.6G. This highlights FDC’s effectiveness in enhancing the network’s comprehension of spatial-channel information, resulting in clearer object recognition and improved detection accuracy as it operates on the deep feature map.

#### Effect of AFF

AFF is engineered to effectively capture interdependencies among adjacent and non-adjacent scale features, thereby intensifying the model’s emphasis on critical features while suppressing irrelevant ones. The incorporation of AFF into the baseline network led to a performance improvement of 2.5%. In addition, integrating it into FAD resulted in a 1.6% improvement, with only a marginal increase of 0.62M parameters in the model. Furthermore, mAPs for classes cyclist and pedestrian show improvements of 2.8% and 3.9% respectively, compared to the baseline. This showcases the effectiveness of AFF in TOD, contributing to remarkable detection results with our approach.

#### Effect of FAD

FAD aims to effectively integrate high- and low-frequency information to enhance texture and semantic features, improving traffic multi- scale object detection in complex traffic scenarios while maintaining computational performance. In addition, FAD could mitigate misalignment issues between classification and localization tasks in complex environments, occlusion and small objects. By introducing FAD, we achieve a state-of-the-art detection performance, with respective mAP values of 90.9% on the KITTI datasets, representing 3.2% increase over the baseline. Furthermore, the model’s parameters and floating-point operations amount to 8.77 M and 39.7 G. In conclusion, FAD demonstrates competitive performance on autonomous driving scenes and facilitates the model in achieving optimal results.

#### Receptive field

**Fig. 2:**
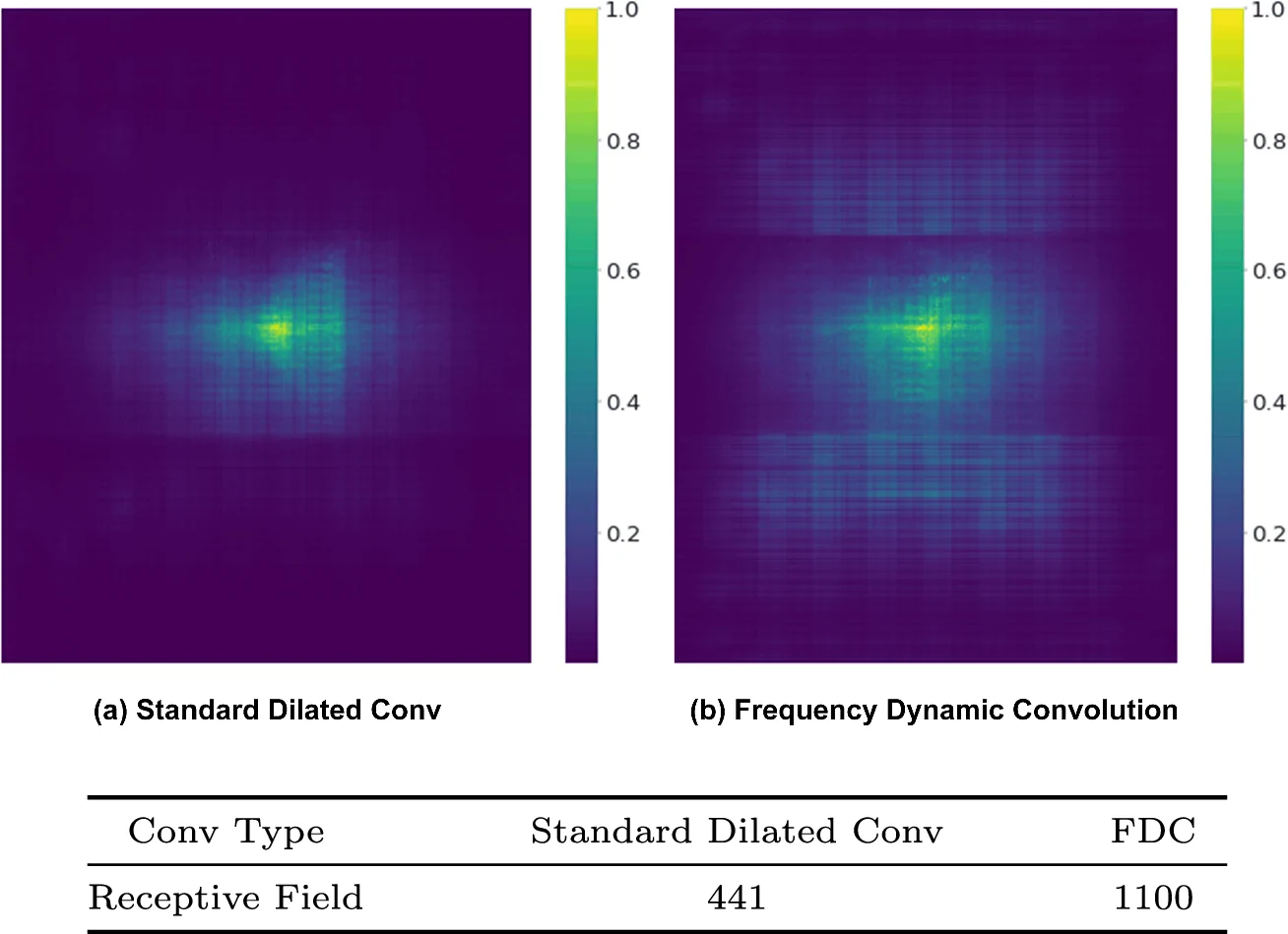
Visualization of theoretical receptive field analysis. Brighter heatmap colors indicate stronger receptive field responses.

Prior work highlights the critical role of large receptive fields in TOD. Unlike conventional dilated DarkNet, which uses a fixed global dilation rate, FDC leverages the adaptive dilation rate strategy to dynamically adjust dilation rates, achieving broader receptive field coverage. As visualized in Fig. 2, FDC enhances this adaptability by prioritizing informative frequency components in feature maps, systematically increasing the average dilation rate and expanding the effective receptive field.

### Comparison experiments and quantitative analysis

In this section, we conduct comparative experiments between FAD and other state-of-the-art detectors on the KITTI, BDD100K and Cityscapes datasets, as presented in Table 3.

#### Results on KITTI

We compare our method with state-of-the-art approaches to the KITTI validation criteria, and detailed results are shown in Table 2. FAD achieves the highest mAP of 90.9%, outperforming all competitors. This highlights the effectiveness of our proposed method. In addition, our approach excels in 2 specific categories: car and cyclist. Notably, FAD achieves an FPS of 57.6, demonstrating the balance between performance and computational resources achieved by our approach.

**Table 2:** Detection performance comparison with related works on the KITTI validation set across classes and difficulties

Method	mAP	Car			Pedestrian			Cyclist			FPS
		Easy	Mod.	Hard	Easy	Mod.	Hard	Easy	Mod.	Hard	
5-Ensemble^70^	73.70	97.84	89.75	80.87	77.08	67.66	59.17	79.45	57.30	54.17	5.10
CertainNet^71^	73.00	93.81	89.36	82.11	76.33	66.13	58.54	78.02	57.49	55.20	16.46
Centernet^72^	71.49	90.30	89.15	82.17	76.53	67.53	59.37	73.48	52.63	50.25	20.21
SSD^73^	60.79	87.34	87.74	77.27	50.38	48.41	43.46	48.25	52.31	52.13	33.17
ASSD^74^	75.28	89.28	89.95	82.11	69.07	62.49	60.18	75.23	76.16	72.83	33.04
SA-YOLO^75^	83.82	91.71	87.16	80.97	86.49	80.63	76.91	87.28	83.44	79.83	32.68
YOLOv8^76^	89.31	98.32	98.50	90.54	90.63	87.65	80.03	89.26	87.01	79.39	69.04
YOLOv10^77^	88.80	98.58	98.60	90.53	90.74	86.69	86.91	89.28	87.04	79.21	66.33
YOLOv11^78^	88.40	98.46	98.37	90.44	90.45	85.00	87.96	87.98	85.76	78.21	85.49
**FAD**	**90.90**	98.55	**98.91**	**90.63**	89.85	**88.16**	**88.13**	**90.84**	**90.33**	**89.71**	57.60

As shown in Table 3, our method surpasses the mainstream YOLO series and RT-DETR in mAP. Notably, it achieves the highest mAP while maintaining exceptional efficiency, with only 8.77 M parameters, making it the most lightweight model among all compared approaches.

#### Results on BDD100K

We conducted a comprehensive evaluation of FAD on the BDD100K dataset, benchmarking it against the latest advancements in the field. FAD notably outperforms its counterparts, achieving a remarkable mAP of over 53.7% (as displayed in Table 3). Our method showcases exceptional generalization capabilities, demonstrating outstanding performance even on larger traffic datasets. Notably, with an input image size of 640 × 640, FAD surpasses all existing detectors, achieving an FPS of 55.6 while maintaining the smallest model size among its peers.

Moreover, our achieved mAP of 53.7% represents a substantial 1.9% improvement over the runner-up, YOLOv11. In addition, the visualization results presented in Supplementary Fig. 3 vividly demonstrate FAD’s precision in detecting objects across diverse scales and aspect ratios, effectively handling complex backgrounds, occlusions, low-quality objects, dense scenes, nighttime conditions, and small objects.

#### Results on cityscapes

As shown in Table 3, FAD attains 50.2% mAP on the Cityscapes dataset, exceeding most existing lightweight detectors, including the latest YOLO series and RT-DETR. Such performance is particularly remarkable given the dataset’s emphasis on densely structured urban environments with frequent occlusions and abundant small objects. These results highlight FAD’s enhanced ability to perceive fine-grained details and maintain detection stability under complex visual conditions, underscoring its robustness in real-world traffic scenarios.

**Table 3:** Quantitative comparisons of the different methods on KITTI, BDD100K and Cityscapes datasets

Method	KITTI		
	mAP	Params	GFLOPS
SA-YOLO^75^	81.8	–	–
YOLOv3^8^	82.2	234.94	65.3
MobileSSD^79^	71.8	33.86	12.5
EdgeYOLO^80^	82.1	24.48	9.9
BHF-MTM^81^	88.6	35.80	–
FD-Head^82^	90.5	–	23.2
YOLOX^12^	89.0	9.00	26.7
YOLOv8^83^	89.3	11.20	28.7
SHW^84^	86.8	8.30	22.9
PIAENet^85^	83.4	17.90	3.2
YOLOv8^76^	89.3	11.40	29.7
YOLOv10^77^	88.8	9.17	24.8
YOLOv11^78^	88.4	9.40	21.5
RT-DETR^86^	88.5	42.70	136.1
**Ours**	**90.9**	**8.77**	39.7

Method	BDD100K		
	mAP	FPS	Params
CertainNet^71^	40.9	–	–
SA-YOLO^75^	17.3	48.5	–
CenterNet^72^	44.5	46.0	93.50
YOLOv7^11^	44.3	71.0	42.00
PASFLN^87^	46.3	40.0	117.00
SDA-OD^88^	42.8	33.0	–
OFFR-YOLO^89^	45.3	–	–
MDNet^90^	45.0	46.0	14.80
SpikeTOD^91^	43.7	–	–
CSIM^92^	50.8	–	–
YOLOv8^76^	51.0	69.8	29.7
YOLOv10^77^	51.8	67.3	24.8
YOLOv11^78^	51.5	80.7	21.5
RT-DETR^86^	51.6	45.1	136.1
**Ours**	**53.7**	55.6	**8.77**

Method	Cityscapes		
	mAP	Params	GFLOPS
MDNet^90^	37.0	15.11	12.3
DAYOLO^93^	49.3	9.35	19.7
PDEM^94^	43.3	–	26.7
UMS^2^-ODNet^95^	48.3	34.9	85.9
Faster R-CNN^96^	40.8	41.5	180.5
YOLOv7^11^	33.5	36.9	55.5
PWNet^97^	49.1	–	–
CPSS-FAT^98^	39.4	48.4	181.1
AugFCOS^99^	42.3	–	–
IRG^100^	47.7	–	–
YOLOv8^76^	47.9	11.40	29.7
YOLOv10^77^	47.3	9.17	24.8
YOLOv11^78^	47.8	9.40	21.5
RT-DETR^86^	48.5	42.70	136.1
**Ours**	**50.2**	**8.77**	39.7

Our proposed method achieves the best metric scores across the three datasets.

#### Results on Waymo 2D

As reported in Table 4, FAD achieves 53.1% mAP@50 and 33.0% mAP@50-95 on the Waymo 2D dataset. Compared with representative YOLO-series detectors and RT-DETR, FAD delivers superior mAP@50 performance while remaining competitive across the vehicle, person, and cyclist categories. These results verify the robustness of FAD in large-scale autonomous driving scenarios characterized by diverse viewpoints, object scales, and complex traffic conditions.

**Table 4:** Quantitative comparisons of the different methods on Waymo 2D datasets

Method	mAP@50	mAP@50-95	Vehicle	Person	Cyclist
YOLOv8^76^	51.0	32.7	64.5	56.2	32.4
RT-DETR^86^	52.3	32.6	65.1	56.7	32.6
YOLOv10^77^	51.8	32.9	65.5	57.0	33.1
YOLOv11^78^	51.0	32.5	64.6	56.6	31.8
FAD	**53.1**	**33.0**	**68.0**	**59.7**	31.7

#### Statistical reliability analysis

To verify that the observed improvements are not caused by stochastic variation, we conduct a statistical analysis on KITTI over five independent runs with different random seeds.

As shown in Supplementary Table 2, our method achieves a mean mAP of 90.5 with a standard deviation of 0.25, indicating stable performance across runs, while the best result reaches 90.9 mAP. The 95% confidence interval (90.3–90.7) further characterizes the uncertainty of the estimated mean. In comparison, YOLOv11 (88.1–88.4) and RT-DETR (88.1–88.5) exhibit non-overlapping confidence intervals, indicating a clear performance margin. Two-sided paired t-tests further confirm the statistical significance of the improvements. Specifically, our method achieves gains of + 2.3 and + 2.2 mAP over YOLOv11 and RT-DETR, with *p*-values of 0.003 and 0.001, respectively. Statistical significance is confirmed, and the observed improvements cannot be attributed to stochastic variation.

### Visualization of detection results

To further substantiate the effectiveness of the proposed FAD, we present a series of qualitative visualizations on the KITTI, BDD100K, and Cityscapes datasets. As illustrated in Supplementary Fig. 3, FAD is evaluated under six representative traffic scenarios in KITTI, exhibiting consistently strong detection performance. The model accurately localizes pedestrians partially concealed by vehicles, overlapping human instances, and distant small-scale objects, highlighting its capacity to handle complex occlusions and scale variations. Moreover, FAD accurately identifies multi-scale and occluded pedestrians and vehicles, demonstrating robustness against cluttered backgrounds and severe illumination fluctuations. Quantitative comparisons in Supplementary Fig. 5 further show improved detection results relative to baseline, with consistent visual evidence of improvement in all evaluated conditions.

**Fig. 3:**
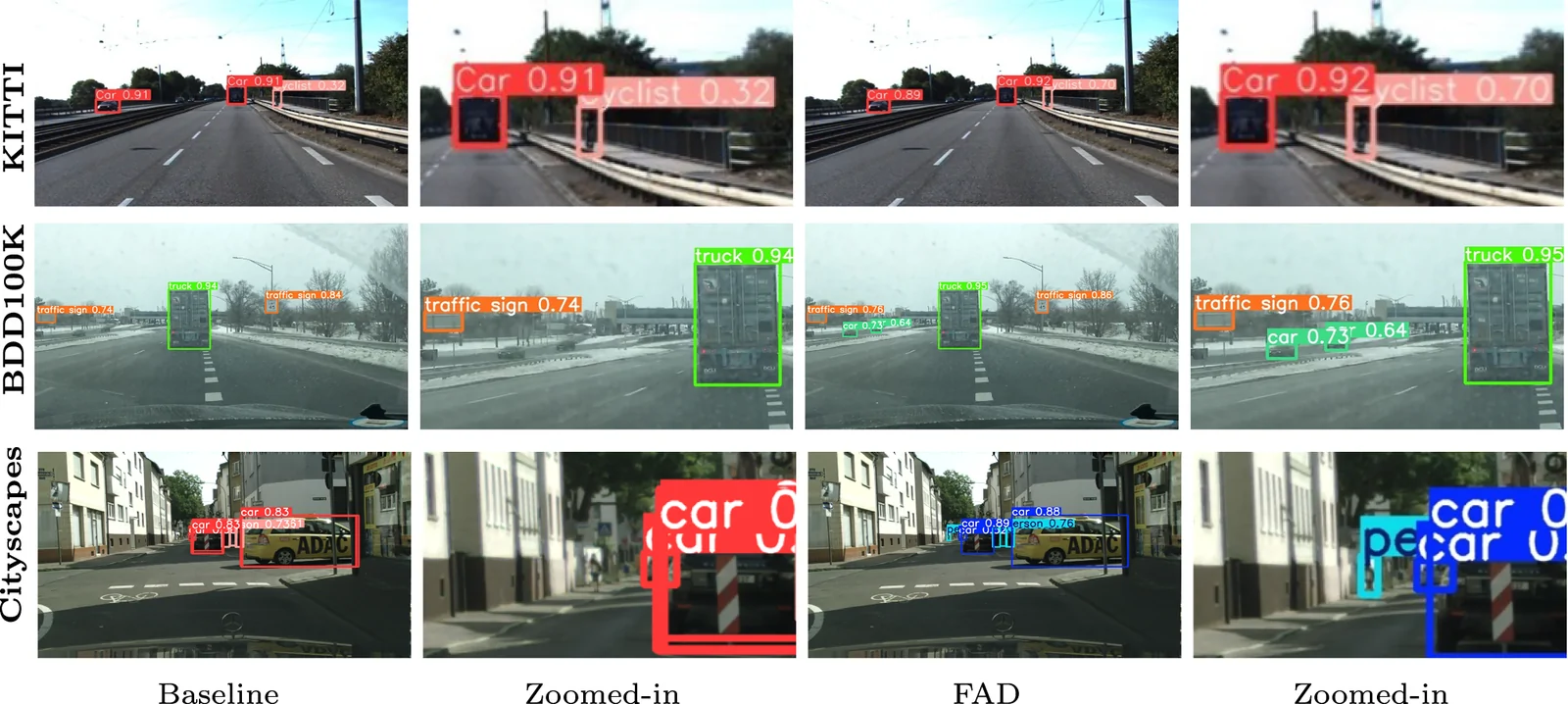
Representative zoom-in view results of FAD on the KITTI, BDD100K and Cityscapes datasets. Since the images we selected contain different-scale objects, zooming in on the page gives a better visual effect. Images from the KITTI Vision Benchmark Suite are used with permission and made available under the CC BY-NC-SA 3.0 licence (https://creativecommons.org/licenses/by-nc-sa/3.0/).

Zoomed-in visualizations in Fig. 3 provide detailed comparisons between FAD and the baseline across datasets. From top to bottom, the rows correspond to KITTI, BDD100K, and Cityscapes. FAD consistently delivers more precise object localization and sharper detection boundaries, maintaining robust performance across diverse visual environments. Specifically, on KITTI, FAD markedly enhances the detection of faint cyclists, improving accuracy from 0.32 to 0.70. On BDD100K, it precisely identifies vehicles in opposing lanes, while on Cityscapes, it successfully captures distant, low-resolution pedestrians. Collectively, these results demonstrate that FAD achieves stable and reliable detection performance across a wide range of challenging traffic scenarios, confirming its strong generalization capability in real-world environments.

Furthermore, by conducting qualitative comparisons between FAD and the baseline, we provide an intuitive illustration of FAD’s improvements in mitigating both missed and false detections. Green boxes indicate correct detections, blue boxes denote missed objects, and red boxes correspond to false positives. FAD enhances object feature representation via frequency-guided feature extraction and adaptive fusion, substantially reducing both missed detections and false positives. This approach improves occlusion handling while preserving robust performance on small and distant objects, such as pedestrians and vehicles.

For BDD100K, as depicted in Supplementary Fig. 3, FAD maintains stable detection performance across diverse environmental conditions. FAD achieves accurate multi-scale object detection across diverse traffic scenarios under both daytime and low-light conditions. As shown in the third-row subfigure, FAD accurately detects multi-scale occluded objects and low-quality small objects caused by perspective distortion from the onboard camera viewpoint. Visualization results on the BDD100K dataset further demonstrate its state-of-the-art performance.

For Cityscapes, which emphasizes dense urban environments characterized by cluttered scenes, occlusions, and small objects, FAD demonstrates accurate detection of occluded and small objects, as further illustrated in Supplementary Fig. 3. Its consistent performance across datasets underscores the effectiveness of our design in addressing the challenges posed by dense layouts and scale variations inherent in urban traffic scenarios.

For Waymo 2D, which features real-world autonomous driving scenarios captured simultaneously from five synchronized on-board cameras, FAD demonstrates robust multi-scale object detection across diverse traffic conditions. As shown in Supplementary Fig. 6, FAD accurately detects occluded vehicles, distant small objects, and objects under varying weather and lighting conditions. These results highlight FAD’s ability to handle complex real-world traffic scenes with multi-camera views, reinforcing its generalization and effectiveness across large-scale autonomous driving datasets.

### In-vehicle computing platform experiments

The in-vehicle computing platform utilized in this study is built upon the Rockchip RK3588 system-on-chip, which offers high-performance heterogeneous computing and multimedia processing capabilities optimized for real-time visual perception and deep learning inference in autonomous driving scenarios. The schematic layout and hardware specifications of the platform are jointly presented in Supplementary Fig. 4.

**Fig. 4:**
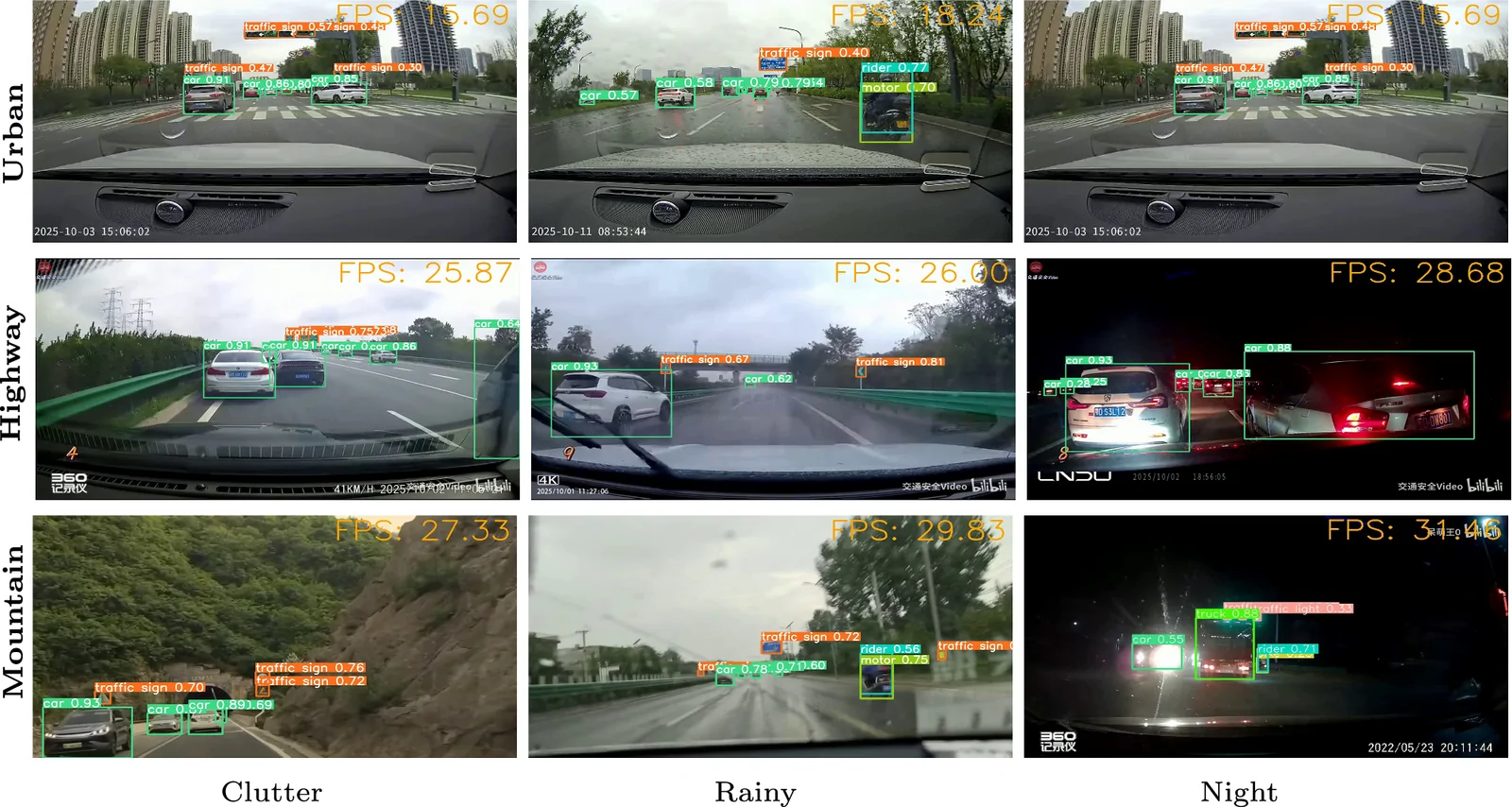
Representative real-time traffic scene detection results of FAD on the RK3588 in-vehicle computing platform, with real-time FPS visualized in the top-right corner. The selected images cover real-world urban, highway, and mountain traffic scenes, with real-time detection achieved on the RK3588 at an average latency of 28.5 ms.

To evaluate the practical performance of the proposed FAD algorithm in dynamic driving conditions, we deployed the inference pipeline primarily on the RK3588’s NPU, while the CPU and GPU handled pre-processing and auxiliary computations. The model was configured with an input resolution of 640 × 640 and converted into the RKNN format to fully exploit the NPU’s parallel acceleration capability. Hardware-level NPU inference reduced computational latency and ensured stable real-time performance. FAD was initialized using pre-trained weights on the BDD100K dataset to ensure robust generalization and performance in real-world traffic scenarios. Experimental results in Fig. 4 indicate that FAD achieves an average inference speed of 35.14 FPS at full input resolution, with an average per-frame latency of 28.5 ms, ensuring real-time processing suitable for in-vehicle deployment. FAD maintains high detection accuracy across varying traffic densities and lighting conditions, with limited impact on mAP. The heterogeneous computing architecture of the RK3588 efficiently balances computational load and energy consumption, enabling the real-time deployment of advanced perception algorithms such as FAD in autonomous driving scenarios.

## Discussion

### Limitations

Despite its demonstrated effectiveness, the proposed FAD framework has certain limitations. Its performance is contingent on the quality of frequency-domain feature decomposition, which may degrade under extreme lighting conditions or adverse weather. Moreover, frequency-domain guided operations inherently incur higher computational costs in terms of GFLOPs, potentially constraining deployment on ultra-low-power edge devices.

### Conclusion

FAD is a lightweight one-stage detector that improves traffic object detection through frequency-guided adaptive feature extraction and fusion. By introducing frequency-adaptive receptive fields and cross-scale frequency-oriented fusion, FAD effectively enhances object details, boundary cues, and semantic consistency while maintaining a compact architecture. Extensive experiments demonstrate that FAD achieves improved detection performance in challenging traffic scenarios, including occlusion, small objects, dense targets, and complex backgrounds. Future work will explore multimodal sensory fusion and more efficient frequency-domain operators to further improve robustness and efficiency.

## Methods

### Frequency dynamic convolution

**Fig. 5:**
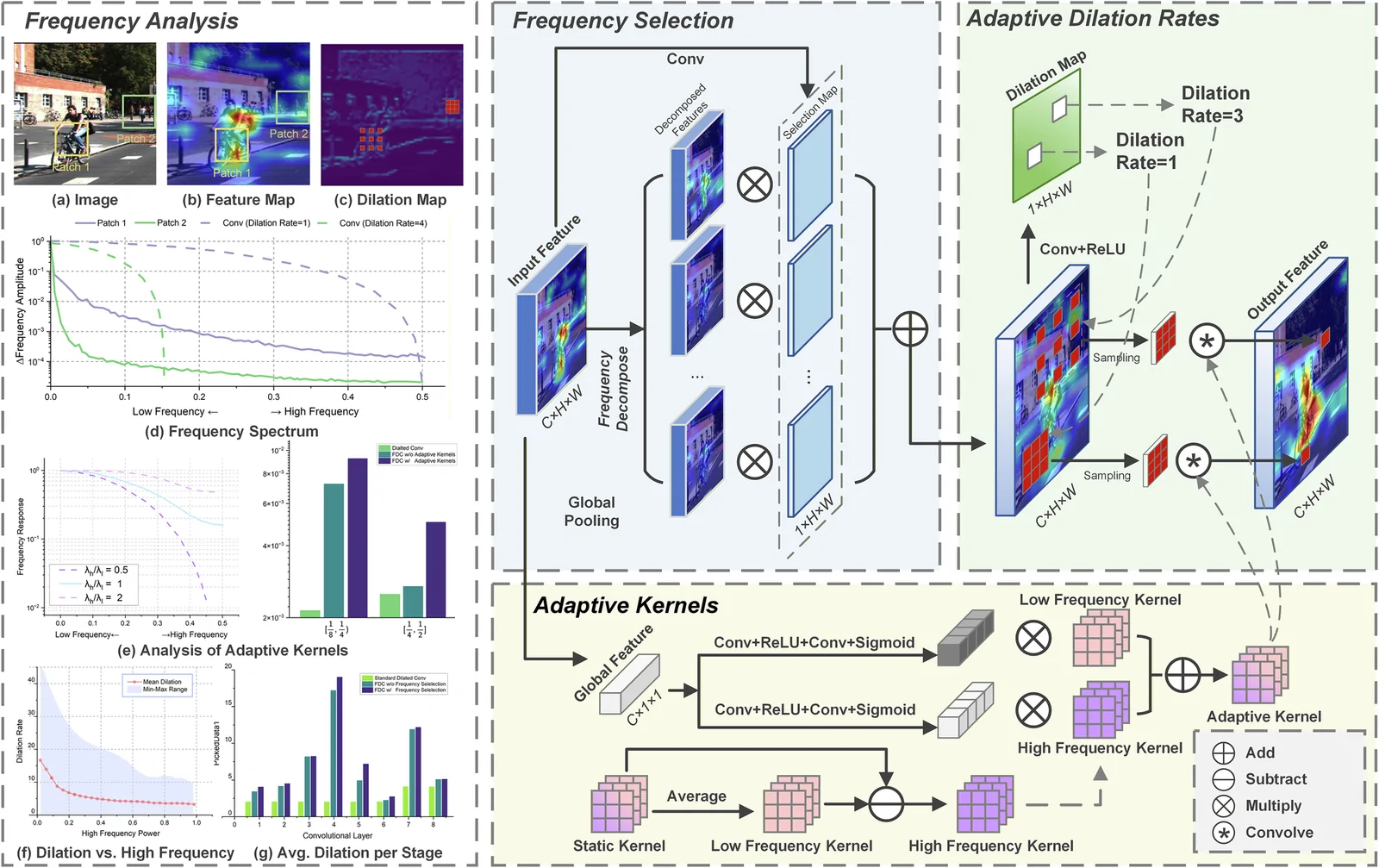
Illustration of the frequency dynamic convolution (FDC) framework with frequency selection, adaptive dilation rates and adaptive kernels. For the input image (**a**), extracted features (**b**) vary spatially. Patch 1 has more high-frequency content, while patch 2 is dominated by low frequencies (**c**). Thus, a small dilation rate for patch 1 preserves effective bandwidth, and a larger rate for patch 2 enables a wider receptive field (**d**). **e** Left: Representative frequency response of a convolutional kernel, where *λ*_*l*_ and *λ*_*h*_ denote adaptive weights for the decomposed low- and high-frequency components. Right: FDC with Adaptive Kernels increases the proportion of high-frequency band power within the ranges $\left[\frac{1}{8},\frac{1}{4}\right)$ and $\left[\frac{1}{4},\frac{1}{2}\right]$, indicating an expansion of the effective bandwidth. **f** Left: The curve shows the relationship between normalized high-frequency power and the predicted dilation rate. FDC with Adaptive Dilated Rates assigns lower dilation rates to regions rich in high-frequency content, such as object boundaries. Right: Mean dilation rates at stages 3 and 4 of the backbone.

An overview of the proposed FDC is provided in Fig. 5. Initially, we present the frequency selection strategy for balancing bandwidth and receptive field, followed by a discussion of adaptive dilation rates and kernels that jointly enhance bandwidth utilization and receptive field size. FDC is designed to address the bandwidth–receptive field trade-off caused by dilation-induced spectral compression, thereby preserving high-frequency sensitivity while enlarging contextual coverage.

### Adaptive dilation rates

**Dilated convolution** is typically denoted as follows:

1 $$\,{{\rm{Y}}}(p)={\sum}_{i=1}^{K\times K}{{{\rm{W}}}}_{i}{{\rm{X}}}\,(p+\Delta {p}_{i}\times D),$$

where Y(*p*) indicates the pixel value at coordinate *p* of the output feature map, *K* is the kernel size, *W*_*i*_ signifies the trainable kernel coefficients, and X(*p* + Δ*p*_*i*_) corresponds to the input feature value at position *p* displaced by Δ*p*_*i*_. The offset $\left.\Delta {p}_{i}\right)$ encodes the *i*-th coordinate in the fixed sampling grid (− 1, − 1); (− 1, 0); …; (+ 1, + 1). The dilation rate *D* governs the expansion of the effective receptive field.

**Frequency analysis**. Previous works have demonstrated that increasing the dilation rate leads to degradation of frequency information^32,63^. As governed by the scaling property of the Fourier transform, increasing the dilation rate from 1 to *D* scales down the convolutional kernel’s frequency response to $\frac{1}{D}$, as depicted in the Fig. 5. Concurrently, the dilated convolution loses its ability to capture frequencies beyond the Nyquist limit, *i*. *e*. , half the sampling rate $\frac{1}{2D}$).

Concretely, we first apply Discrete Fourier Transform (DFT) to map the input features $\,{{\rm{X}}}\,\in {{\mathbb{R}}}^{H\times W}$ to the frequency domain, ${{{\rm{X}}}}_{F}={{{{\mathcal{F}}}}}(\,{{\rm{X}}})$:

2 $${{{\rm{X}}}}_{F}(u,v)=\frac{1}{HW}{\sum}_{h=0}^{H-1}{\sum}_{w=0}^{W-1}{{{\rm{X}}}}\,(h,w){e}^{-2\pi j(uh+vw)},$$

where ${{{{\mathcal{F}}}}}\in {{\mathbb{R}}}^{H\times W}$ denotes the output array of complex numbers from DFT. *H* and *W* represent the height and width of the feature, while *h* and *w* denote the coordinates in the feature map X. ∣*u*∣ and ∣*v*∣ represent the normalized frequencies in the height and width dimensions. After shifting the low-frequency components to the center, *u* is drawn from the set $\{-\frac{H}{2},-\frac{H+1}{2},\ldots,\frac{H-1}{2}\}$, *v* is drawn from the set $\{-\frac{W}{2},-\frac{W+1}{2},\ldots,\frac{W-1}{2}\}$. As a result, frequencies exceeding the Nyquist rate ${{{{{\mathcal{H}}}}}}_{D}^{+}=\{(u,v)| | k| > \frac{1}{2D}\,{{{\rm{or}}}}\,| l| > \frac{1}{2D}\}$ cannot be accurately sampled, limiting the available bandwidth.

**Adaptive dilation rate**. To summarize the above, selecting dilation rates requires balancing receptive field and bandwidth. Given spatial variations in feature maps, optimal rates may differ per pixel. We propose Adaptive Dilation Rate, dynamically assigning pixel-wise rates for better optimization.

3 $$\,{{\rm{Y}}}(p)={\sum}_{i=1}^{K\times K}{{{\rm{W}}}}_{i}{{\rm{X}}}\,(p+\Delta {p}_{i}\times \hat{\,{{\rm{D}}}\,}(p)),$$

$\left.\hat{\,{{\rm{D}}}\,}(p)\right)$ predicted by applying a convolutional layer parameterized by *θ*. Specifically, we introduce a ReLU layer to maintain non-negative dilations while utilizing a modulation mechanism^34^. This design optimizes the receptive field while minimizing frequency information loss at each pixel. We denote the local feature centered at point *p* with window size *s* as *X*^(*p*; *s*)^. The receptive field $\,{{\rm{RF}}}\,(p)=(K-1)\times \left.\hat{\,{{\rm{D}}}\,}(p)\right)+1$ exhibits a positive correlation with $\left.\hat{\,{{\rm{D}}}\,}(p)\right)$. The frequency components within the set ${{{{{\mathcal{H}}}}}}_{\left.\hat{\,{{\rm{D}}}\,}(p)\right)}^{+}$ cannot be precisely reconstructed. Consequently, the information loss can be quantified by computing the high-frequency power: $\,{{\rm{HP}}}\,(p)={\sum}_{{{{{{\mathcal{H}}}}}}_{\left.\hat{\,{{\rm{D}}}}(p)\right)}^{+}}| {{{\rm{X}}}\,}_{F}^{p,s}(u,v)| ^{2}$. Thus, the parameter *θ* can be optimized as:

4 $$\theta={{\max }_{\theta }}\left(\sum \,{{\rm{RF}}}(p)-\sum {{\rm{HP}}}\,(p)\right),$$

Since the frequency set ${{{{{\mathcal{H}}}}}}_{\left.\hat{\,{{\rm{D}}}\,}(p)\right)}^{+}$ is discrete and HP is non-differentiable, direct optimization becomes impractical. Therefore, we implement direct optimization of $\left.\hat{\,{{\rm{D}}}\,}(p)\right)$ through dual mechanisms: (1)Dilation rate increase at low-HP(*p*) positions to broaden receptive fields (2)Dilation rate suppression at high-HP(*p*) positions to conserve frequency information This optimization is formally expressed as:

5 $$\theta={{\max }_{\theta }}\left({\sum}_{{p\in} {{{\rm{HP}}}}^{-}}\hat{\,{{\rm{D}}}}(p)\right)-{\sum}_{p\in} {{{\rm{HP}}}}^{+}\hat{\,{{\rm{D}}}\,}(p)\left.\right)\left.\right),$$

where HP^+^ and HP^−^ denote correspond to pixels with the highest/lowest (*e*. *g*., 25%) high-frequency power values respectively, manifesting as the brighter/darker regions in Fig. 5.

### Adaptive kernels

Adaptive Dilation Rate establishes an optimized trade-off between spectral coverage and spatial context by adaptively assigning pixel-specific dilation rates. This dual-optimization framework considers both the frequency-sensitive effective bandwidth (inherently linked to kernel weights) and receptive field dimensions. While traditional convolution kernels learn to capture features across diverse frequency bands, which are crucial for comprehending intricate visual patterns, but they become static once trained. Our approach further improves effective bandwidth by: (1) decomposing kernel parameters into low- and high-frequency components, and (2) introducing dynamic weighting for tunable frequency response, a lightweight operation adding minor additional parameters and computational overhead. For static convolution kernels, the fixed weight W is represented as:

6 $$\,{{\rm{W}}}\,=\bar{{{{{\rm{W}}}}}}+\hat{\,{{\rm{W}}}\,}.$$

Wherein, $\bar{{{{{\rm{W}}}}}}=\frac{1}{K\times K}{\sum }_{i=1}^{K\times K}{{{\rm{W}}}}_{i}$ denotes the averaged kernel-wise W. It operates as a low-pass *K* × *K* mean filter, followed by a 1 × 1 convolution with parameters specified by $\bar{{{{{\rm{W}}}}}}$. As mentioned in ^64^, larger mean values tend to suppress high-frequency components. The term $\,{{\rm{W}}}\,=\bar{{{{{\rm{W}}}}}}-\hat{\,{{\rm{W}}}\,}$ represents the residual, which captures local variations and highlights the high-frequency components. After decomposition, our Adaptive Kernel dynamically adjusts both high and low-frequency components, and can be formally expressed as:

7 $$\,{{\rm{W}}}\,={\lambda }_{l}\bar{{{{{\rm{W}}}}}}+{\lambda }_{h}\hat{\,{{\rm{W}}}\,},$$

where *λ*_*l*_ and *λ*_*h*_ represent the dynamic weights for each channel, which are predicted using a global pooling followed by convolution layers. Adjusting the ratio of $\frac{{\lambda }_{l}}{{\lambda }_{h}}$ dynamically according to the input context enables the network to focus on specific frequency bands and better adapt to the complexity of visual patterns in the features. This dynamic frequency adaptation improves the capacity of the network to capture both low-frequency context and high-frequency local details, effectively expanding bandwidth and improving performance in segmentation tasks that require the extraction of diverse characteristics at different frequencies.

### Frequency selection strategy

According to previous studies^65^, traditional convolution typically acts as a high-pass filter, causing the resulting features to emphasize high-frequency components. To maintain a high effective bandwidth, smaller dilation rates are often used, which, however, limits the receptive field size. Frequency selection strategy is designed to address this issue by balancing high- and low-frequency components, thereby improving the receptive field in feature representations. Specifically, the Frequency Selection Strategy performs an initial decomposition of the features into distinct frequency bands by applying specialized masks in the Fourier domain:

8 $${{{\rm{X}}}}_{b}={{{{{\mathcal{F}}}}}}^{-1}({{{{{\mathcal{M}}}}}}_{b}{{{\rm{X}}}}_{F}),$$

where ${{{{{\mathcal{F}}}}}}^{-1}$ indicates the inverse Fast Fourier Transform, and ${{{{{\mathcal{M}}}}}}_{b}$ is a binary mask applied to extract the corresponding frequency component.

9 $${{{{{\mathcal{M}}}}}}_{b}(u,v)=\left\{\begin{array}{ll}1\quad &{{{{\rm{if}}}}}{\phi }_{b}\le \max (| u|,| v| ) < {\phi }_{b+1}\\ 0\quad &{{{{\rm{otherwise}}}}}\hfill\end{array}\right..$$

Here, *ϕ*_*b*_ and *ϕ*_*b*+1_ correspond to the *B* + 1 predefined frequency thresholds $\{0,{\phi }_{1},{\phi }_{2},\ldots,{\phi }_{B-1},\frac{1}{2}\}$. Subsequently, the frequency selection strategy dynamically adjusts the weights of the frequency components across different frequency bands in a spatial manner. This is expressed as:

10 $$\hat{\,{{\rm{X}}}}(i,j)={\sum}_{b=0}^{B-1}{{{\rm{A}}}}_{b}(i,j){{{\rm{X}}}}_{b}(i,j),$$

where $\hat{\,{{\rm{X}}}\,}(i,j)$ represents the learned frequency-balanced feature after frequency selection, and ${{{\rm{A}}}}_{b}\in {{\mathbb{R}}}^{H\times W}$ denotes the selection map for the *b*-th frequency band. Specifically, we decompose the frequency into four bands in an octave-wise manner^66^, *i*. *e*. , $[0,\frac{1}{16}),[\frac{1}{16},\frac{1}{8}),[\frac{1}{8},\frac{1}{4})$, and $[\frac{1}{4},\frac{1}{2}]$.

### Adaptive Frequency-oriented Fusion

**Fig. 6:**
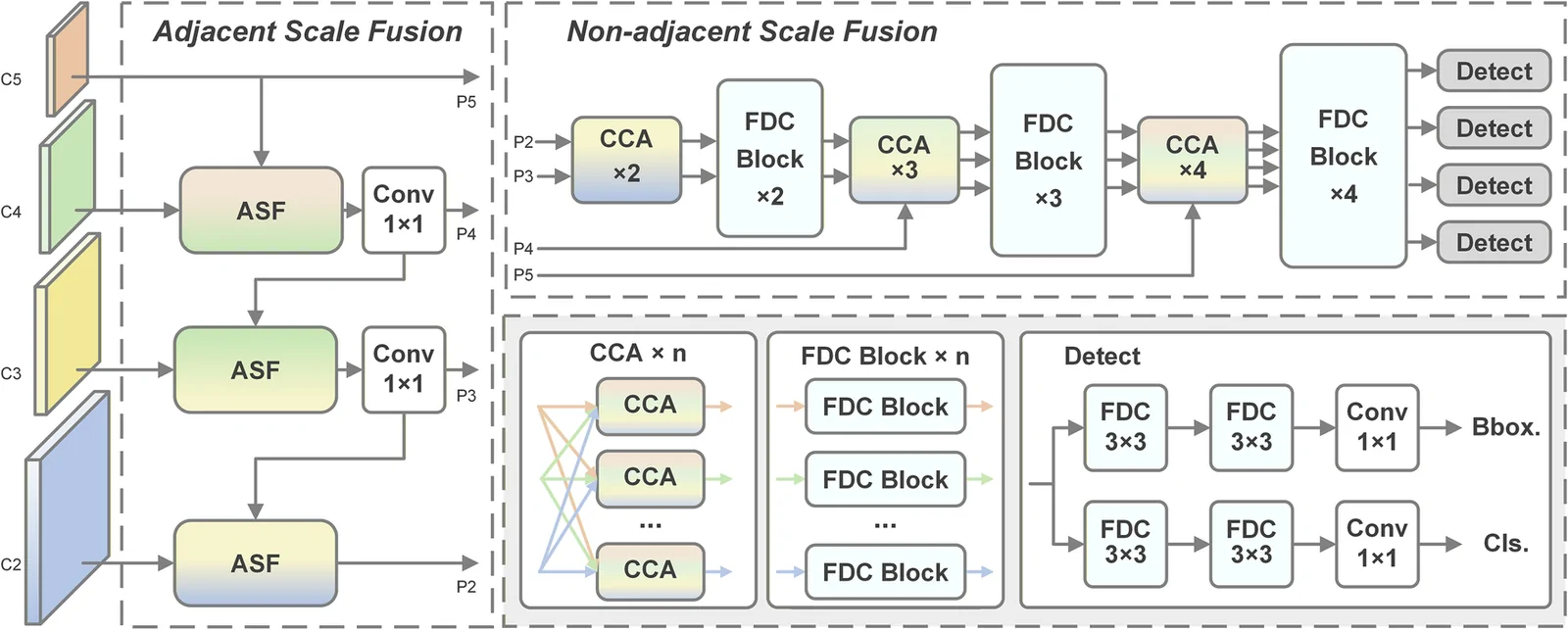
Illustration of the adaptive frequency-oriented fusion (AFF). AFF consists of two stages: adjacent scale fusion and non-adjacent scale fusion. Non-adjacent scale fusion begins with two low-level features, continues with higher-level features, and incorporates top-level features in the final stage.

**Fig. 7:**
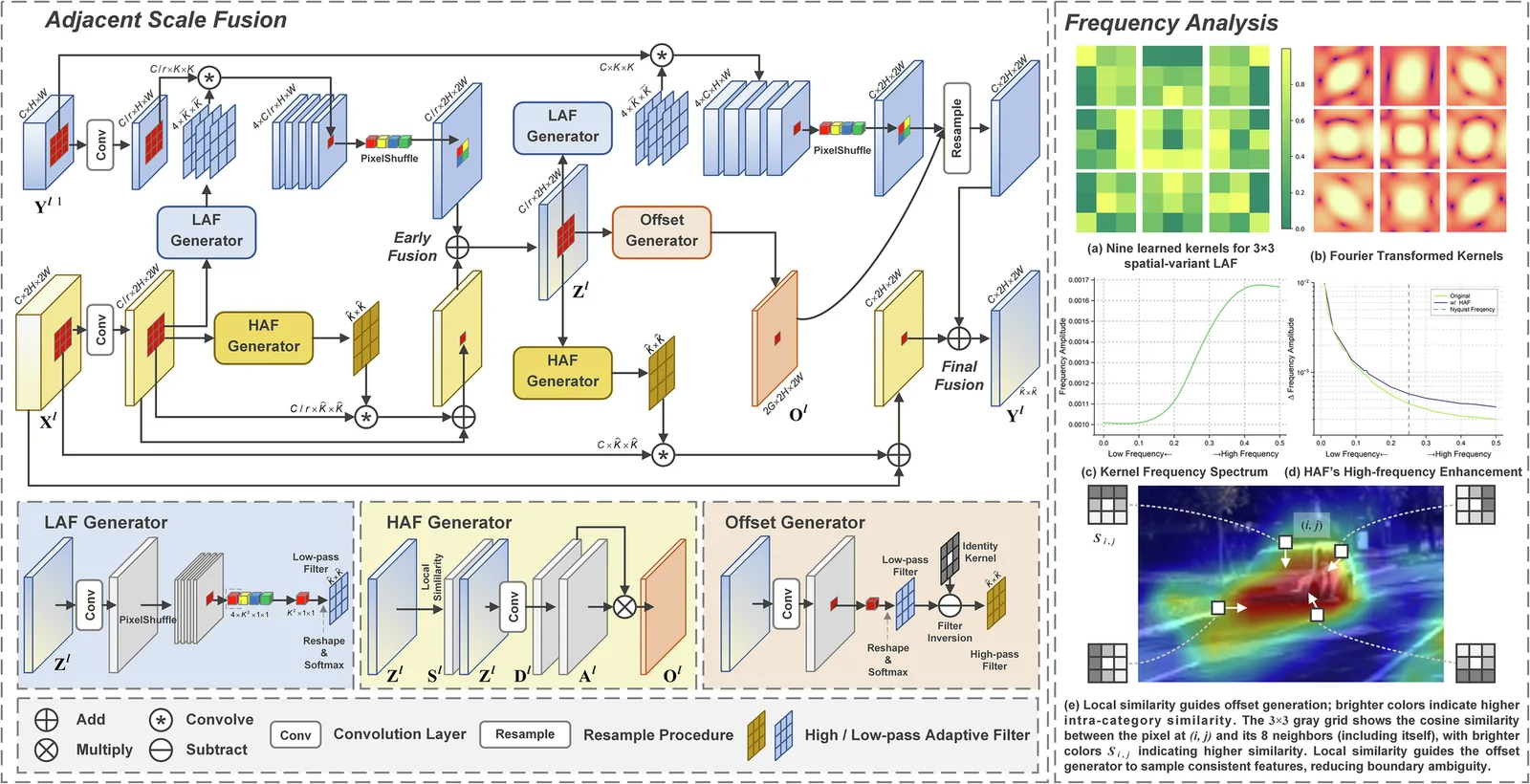
Illustration of frequency-oriented adjacent-scale fusion. Pixel unshuffle reduces spatial size by half and expands channels 4 × , *e*. *g*. , from*C* × 2*H* × 2*W* to 4 × *C* × *H* × *W*. Pixel shuffle is the reverse operation. The LAF generator and HAF in the initial fusion share parameters with those in the final fusion.  ⊗ represents element-wise multiplication, and  ⊖ represents subtraction. **a** Visualizes nine learned 3 × 3 kernels for spatially variant low-pass filters, with brightness indicating weight magnitude. **b** Shows their Fourier transforms. **c** The averaged frequency spectrum reveals stronger high-frequency responses, suggesting the filters depend on high-frequency features for prediction. **d** Quantitative frequency analysis (log-scale) demonstrates that the HAF generator amplifies high-frequency components, improving feature detail and boundary sharpness. **e** Local similarity guides resampling offset prediction.

**Fig. 8:**
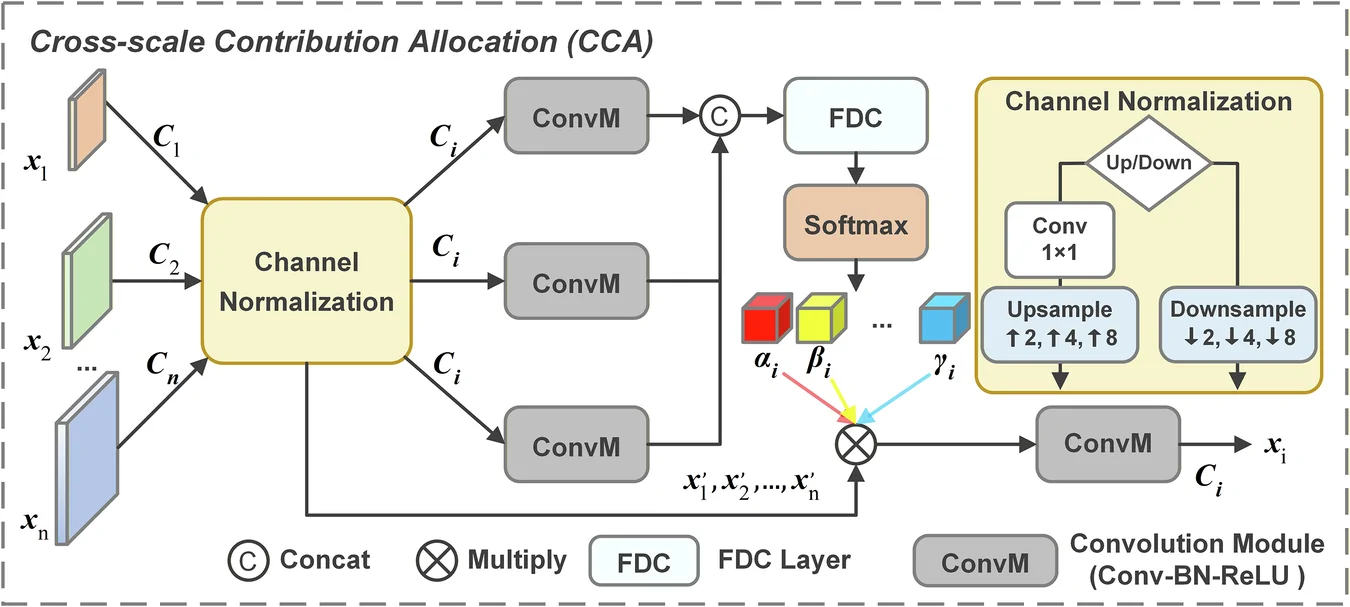
Illustration of cross-scale contribution allocation (CCA), which performs frequency-oriented adaptive spatial fusion. CCA fuses feature maps with different channel dimensions and outputs features with the original channel size.

In this section, we present the Adaptive Frequency-oriented Fusion (AFF) framework, as depicted in Fig. 6. AFF consists of two sequential stages, namely adjacent-scale fusion and non-adjacent-scale fusion. Specifically, as shown in Fig. 7, the adjacent-scale fusion stage comprises three key components: the low-pass adaptive filter generator (LAF), the offset generator, and the high-pass adaptive filter generator (HAF). As shown in Fig. 8, the non-adjacent-scale fusion stage employs cross-scale contribution allocation (CCA) as its key component. AFF is designed to mitigate the spectral imbalance introduced during multi-scale feature fusion by adaptively reallocating low- and high-frequency components for feature consistency and boundary precision.

### Low-pass adaptive filter generator

Low-pass Adaptive Filter Generator (LAF) predicts dynamic low-pass filters to smooth high-level features and reduce feature inconsistency^60^, followed by upsampling of the high-level feature. To generate effective adaptive low-pass filters, it is essential to leverage both high- and low-level features^57^. The LAF generator utilizes the fused Z^*l*^ as input and predicts spatially variant low-pass filters, consisting of a 3 × 3 convolutional layer followed by a softmax layer, as shown below:

11 $${\bar{{{{{\rm{V}}}}}}}^{l}={{{\rm{Conv}}}}_{3\times 3}\left({{{\rm{Z}}}}^{l}\right),$$

12 $${\bar{{{{{\rm{W}}}}}}}_{i,j}^{l,p,q}=\,{{\rm{Softmax}}}\,\left({\bar{{{{{\rm{V}}}}}}}_{i,j}^{l}\right)=\frac{\exp \left({\bar{{{{{\rm{V}}}}}}}_{i,j}^{l,p,q}\right)}{{\sum}_{p,q\in \Omega }\exp \left({\bar{{{{{\rm{V}}}}}}}_{i,j}^{l,p,q}\right)},$$

where ${\bar{{{{{\rm{V}}}}}}}_{l}\in {{\mathbb{R}}}^{{\bar{K}}^{2}\times 2H\times 2W}$ represents the spatially-variant filter weights, $\bar{K}$ is the low-pass filter kernel size. After reshaping, it contains $\bar{K}\times \bar{K}$ filters at each position. A kernel-wise softmax ensures the filters are positive and sum to one, producing smooth low-pass filters in $\bar{{{{{\rm{W}}}}}}\in {{\mathbb{R}}}^{{\bar{K}}^{2}\times 2H\times 2W}$^67^.

Subsequently, we upscale ${{{\rm{Y}}}}_{l+1}\in {{\mathbb{R}}}^{C\times H\times W}$ using a sub-pixel upsampling technique ^68^. Specifically, ${\bar{{{{{\rm{W}}}}}}}^{l}$ is reshaped using a pixel unshuffle method ^68^, halving the height and width while increasing the channel dimension by a factor of 4. The channels are divided into 4 groups, each containing a spatially-variant low-pass filter ${\bar{{{{{\rm{W}}}}}}}^{l,g}\in {{\mathbb{R}}}^{{\bar{K}}^{2}\times H\times W}$, where *g* ∈ {1, 2, 3, 4}. This results in 4 groups of low-pass filtered features, ${\tilde{{{\rm{Y}}}}}^{l+1,g}\in {{\mathbb{R}}}^{C\times H\times W}$, which are then rearranged to form a 2 × upsampled feature ${\tilde{{{\rm{Y}}}}}^{l+1,g}\in {{\mathbb{R}}}^{C\times 2H\times 2W}$:

13 $${\tilde{\,{{\rm{Y}}}\,}}_{i,j}^{l+1}={\sum }_{p,q\in \Omega }{\bar{{{{{\rm{W}}}}}}}_{i,j}^{l,g,p,q}\cdot {\,{{\rm{Y}}}\,}_{i+p,j+q}^{l+1},$$

14 $${\tilde{{{\rm{Y}}}}}^{l+1}={{\rm{PixelShuffle}}}\,\left({\tilde{{{\rm{Y}}}}}^{l+1,1},{\tilde{{{\rm{Y}}}}}^{l+1,2},{\tilde{{{\rm{Y}}}}}^{l+1,3},{\tilde{{{\rm{Y}}}}}^{l+1,4}\right).$$

Spatially variant low-pass filters are adaptively predicted by the LAF generator to enhance feature consistency. As shown in Fig. 1, the visual results reveal that bilinear upsampling in feature fusion causes significant intra-category inconsistency and boundary displacement, as seen in the car’s interior, where similarity is low, and the boundary is blurred. In contrast, the second column of Fig. 1 shows more coherent features with improved interior consistency and sharper boundaries, highlighting the effectiveness of the LAF generator in reducing intra-category variation.

### Offset generator

Although the LAF generator improves intra-category consistency through smoothing, it struggles with large, inconsistent regions and fine boundary details. Larger filters help with broader inconsistencies but blur thin structures, while smaller filters preserve edges but are less effective for wide areas of inconsistency.

To tackle this challenge, we introduce an offset generator. Inspired by the observation that adjacent features with low intra-category similarity may still align closely with highly consistent intra-category features. The offset generator initiates its operation by calculating local cosine similarity metrics:

15 $${\,{{\rm{S}}}}_{i,j}^{l,p,q}=\frac{{\sum }_{c=1}^{c}{{{\rm{Z}}}}_{c,i,j}^{l}\cdot {{{\rm{Z}}}}_{c,i+p,j+q}^{l}}{\sqrt{{\sum }_{c=1}^{c}{({{{\rm{Z}}}}_{c,i,j}^{l})}^{2}}\sqrt{{\sum }_{c=1}^{c}{({{{\rm{Z}}}}_{c,i+p,j+q}^{l})}^{2}}},$$

where $\,{{\rm{S}}}\,\in {{\mathbb{R}}}^{8\times H\times W}$ encodes the cosine similarity between each pixel and its eight neighboring pixels. This similarity map guides the offset generator to sample toward regions with strong intra-category consistency, thereby reducing ambiguity in boundary areas and intra-category inconsistent regions.

The offset generator takes Z^*l*^ and S as inputs to predict the offsets, using two 3 × 3 convolutional layers to estimate the offset direction and scale, as follows:

16 $${{{\rm{O}}}}^{l}={{{\rm{D}}}}^{l}\cdot {{{\rm{A}}}}^{l},$$

17 $${{{\rm{D}}}}^{l}={{\rm{Conv}}}3\times 3({{\rm{Concat}}}({{{\rm{Z}}}}^{l},{{{\rm{S}}}}^{l})),$$

18 $${{{\rm{A}}}}^{l}={{\rm{Sigmoid}}}\,\left(\,{{\rm{Conv}}}3\times 3({{\rm{Concat}}}({{{\rm{Z}}}}^{l},{{{\rm{S}}}}^{l}))\right.,$$

where ${{{\rm{D}}}}^{l}\in {{\mathbb{R}}}^{2G\times H\times W}$ represents the offset direction, ${{{\rm{A}}}}^{l}\in {{\mathbb{R}}}^{2G\times H\times W}$ controls the offset magnitude, and ${{{\rm{O}}}}^{l}\in {{\mathbb{R}}}^{2G\times H\times W}$ corresponds to the final predicted offsets for each pixel in the high-level feature map. The parameter *G* represents the number of offset groups, where the feature map is divided into distinct groups, each with unique spatial offsets to enable finer resampling. This approach facilitates replacing features with low intra-category similarity with those that exhibit higher similarity, allowing the offset generator to address large regions of inconsistent features and enhance boundary refinement, as illustrated in Fig. 7e.

The offset generator directs offsets towards the interior at inner boundaries for better feature consistency, while at outer boundaries, it points them outward to improve clarity. This contrast results in more consistent features and precise boundaries.

### High-pass adaptive filter generator

While the LAF and offset generator enhance high-level features with better intra-category consistency and refined boundaries, they cannot fully recover the detailed boundary information lost during downsampling from lower-level features. The Nyquist-Shannon Sampling Theorem^61,62^ states that frequencies above the Nyquist frequency (half the sampling rate) are lost during downsampling. For example, when downsampling a high-level feature by a factor of 2, frequencies above $\frac{1}{4}$ are aliased. Specifically, we utilize the Discrete Fourier Transform (DFT) to convert the feature map $\,{{\rm{X}}}\,\in {{\mathbb{R}}}^{C\times H\times W}$ into the frequency domain, denoted as ${{{\rm{X}}}}_{F}={{{{\mathcal{F}}}}}(X)$, as demonstrated in Equation (2). High-frequency components exceeding the Nyquist limit, defined as ${{{{{\mathcal{H}}}}}}_{D}^{+}=\{(u,v)| | k| > \frac{1}{4}\,{{\rm{or}}}\,| l| > \frac{1}{4}\}$, are subject to aliasing and cannot be recovered in the downsampled high-level features.

To recover fine boundary details lost during downsampling, the HAF generator is introduced. HAF operates on the initially fused feature Z^*l*^ and generates adaptive high-pass filters with spatial variation. The generator includes a 3 × 3 convolutional layer, a softmax layer, and a subsequent filter inversion step, defined as follows:

19 $${\hat{{{\rm{V}}}}}^{l}={{\rm{Conv}}}3\times 3({{{\rm{Z}}}}^{l}),$$

20 $${\hat{\,{{\rm{W}}}\,}}_{i,j}^{l,p,q}=	 \,{{\rm{E}}}-{{\rm{Softmax}}}\,\left({\hat{\,{{\rm{V}}}\,}}_{i,j}^{l}\right)\\ =	 {{{\rm{E}}}}^{p,q}-\frac{\exp \left({\hat{\,{{\rm{V}}}\,}}_{i,j}^{l,p,q}\right)}{{\sum}_{p,q\in \Omega }\exp \left({\hat{\,{{\rm{V}}}\,}}_{i,j}^{l,p,q}\right)},$$

where ${\hat{{{\rm{V}}}}}^{l}\in {{\mathbb{R}}}^{{\hat{K}}^{2}\times H\times W}$ denotes the preliminary kernels at each spatial coordinate (*i*, *j*), with $\hat{K}$ indicating the high-pass filters kernel size. Following^69^, we generate high-pass kernels ${\hat{{{\rm{W}}}}}_{l}$ by first computing low-pass kernels via kernel-wise softmax, then invert the kernels by subtracting them from **E**. whose weights are [0, 0, 0], [0, 1, 0], [0, 0, 0] when $\hat{K}=3$. After applying high-pass filters and residual addition, the results are obtained as:

21 $${\tilde{\,{{\rm{X}}}}}_{i,j}^{l}={{{\rm{X}}}\,}_{i,j}^{l}+{\sum}_{p,q\in \Omega }{\hat{\,{{\rm{W}}}}}_{i,j}^{l,p,q}\cdot {{{\rm{X}}}\,}_{i,j}^{l}.$$

The HAF generator effectively improves boundary details. For instance, the original feature struggles to define the car outline and a person’s head clearly. With the HAF generator, these boundaries are enhanced, producing finer, more precise low-level features. Figure 7d illustrates that the HAF generator amplifies high-frequency components beyond the Nyquist frequency. The results show the generator’s effectiveness in capturing and preserving critical details, important for a regression task demanding high-resolution features.

### Cross-scale contribution allocation

To assign different spatial weights to features of different levels during the multi-level feature fusion process, enhance the importance of key levels, and mitigate the impact of conflicting information from different objects, we propose the Cross-scale Contribution Allocation module (CCA) to fuse the features of four scales, as shown in Fig. 8.

We define ${x}_{ij}^{n\to l}$ as the feature vector at position (*i*, *j*) projected from layer *n* to layer *l*. The final output feature vector, denoted as ${y}_{ij}^{l}$, is adaptively aggregated across multiple layers through spatial fusion, and can be expressed as a linear combination of ${x}_{ij}^{1\to l}$, ${x}_{ij}^{2\to l}$, and ${x}_{ij}^{3\to l}$ as follows:

22 $${y}_{ij}^{l}={\alpha }_{ij}^{l}\cdot {x}_{ij}^{1\to l}+{\beta }_{ij}^{l}\cdot {x}_{ij}^{2\to l}+{\gamma }_{ij}^{l}\cdot {x}_{ij}^{3\to l}$$

where *l*, ${\alpha }_{ij}^{l}$, ${\beta }_{ij}^{l}$, and ${\gamma }_{ij}^{l}$ correspond to the spatial weights assigned to the four respective feature levels, constrained by ${\alpha }_{ij}^{l}+{\beta }_{ij}^{l}+{\gamma }_{ij}^{l}=1.$ Each feature vector *x*_1→*l*_, *x*_2→*l*_, *x*_3→*l*_ is initially compressed to 8 channels through convolution. After concate nation along the channel dimension, a further convolutional operation reduces the combined feature to 3 channels. Finally, applying Softmax over the channel axis yields the coefficients {*α*^*l*^, *β*^*l*^, *γ*^*l*^}. To accommodate the varying numbers of fused features in different stages of the AFF, we introduce a stage-specific number of adaptive spatial fusion modules in each stage.

## Supplementary information

supplementary information

Transparent Peer Review file

## Source data

source data

## Data Availability

The KITTI data used in this study are available from the KITTI Vision Benchmark Suite at https://www.cvlibs.net/datasets/kitti/. The BDD100K data used in this study are available from the BDD100K database at https://bair.berkeley.edu/blog/2018/05/30/bdd/. The Cityscapes data used in this study are available from the Cityscapes database at https://www.cityscapes-dataset.com/. The Waymo Open Dataset data used in this study are available from the Waymo Open Dataset database at https://waymo.com/open/. The numerical data generated in this study are provided in the Source Data file. Source data are provided in this paper.

## Code availability

The code used in this study is available at GitHub: https://feekyzz.github.io/Frequency-oriented-Adaptive-Real-Time-Object-Detector-for-Cluttered-Traffic-Scenes/, and has been archived in Zenodo with the https://doi.org/10.5281/zenodo.20746878.
