# Supplementary material for: Frequency-oriented adaptive real-time object detector for cluttered traffic scenes
Source: Nat Commun. 2026 Sep 14;17:9787. doi: 10.1038/s41467-026-76346-1 (PMC13575256; doi:10.1038/s41467-026-76346-1)
Supplement: Supplementary file 1 [file 41467_2026_76346_MOESM1_ESM.pdf]

# Supplementary Information

## Frequency-oriented Adaptive Real-Time Object Detector for Cluttered Traffic Scenes

Ziqi Li[1], Tao Gao[2,*], Shutao Li[1,*], Ting Chen[1], Yisheng An[1], Yuanbo Wen[1], and Tao Lei[3]

[1]School of Information Engineering, Chang'an University, Xi'an 710064, China
[2]School of Data Science and Artificial Intelligence, Chang'an University, Xi'an 710064, China
[3]School of Electronic Information and Artificial Intelligence, Shaanxi University of Science and Technology, Xi'an 710021, China

*Correspondence: gtnwpu@126.com; shutao_li@hnu.edu.cn

### Frequency Domain Learning

Frequency-domain analysis has long been established as a fundamental technique in traditional signal processing [38, 39], and recent studies have increasingly adopted it within deep learning contexts to enhance model efficiency and representation capacity, particularly for visual tasks where spatial structures can be effectively decomposed and analyzed in the spectral domain. Rahaman *et al.* [101] and Xu *et al.* [102] identify a tendency of deeper hidden layers to favor lower-frequency components during training, resulting in a learning bias toward low-frequency modes, a phenomenon referred to as the spectral bias or frequency principle. Zhang *et al.* [103] examine the impact of frequency aliasing on the shift-invariance of contemporary models, and AdaBlur [98] addresses this issue by incorporating content-aware low-pass filters during downsampling to mitigate aliasing effects. Similarly, FLC [104] demonstrates that frequency aliasing can substantially compromise model robustness. Qin *et al.* [105] and Magid *et al.* [100] incorporate additional frequency components derived from discrete cosine transform (DCT) coefficients into channel attention mechanisms, thereby improving feature representation. Huang *et al.* [42] apply the classical convolution theorem to develop adaptive frequency filters that function as global token mixers within DNNs. Furthermore, a range of frequency-domain techniques is integrated into network architectures to facilitate the modeling of non-local dependencies [40–44]. Chen *et al.* [106] employ low-pass filters to suppress high-frequency noise introduced by image degradation, effectively enhancing the performance of instance segmentation under low-light conditions. In parallel, numerous studies demonstrate that adversarial attacks can be realized by manipulating high-frequency components [60, 107, 108]. For example, Luo *et al.* [60] show that perturbations in the high-frequency domain significantly reduce intra-category similarity, thereby deteriorating the quality of learned representations. Therefore, our proposed approach introduces a frequency-domain interpretation of dilated convolution to better motivate and strengthen its effectiveness for visual perception tasks, enhancing its capacity to capture features across diverse frequency components. In addition, intra-category inconsistency and boundary displacement are

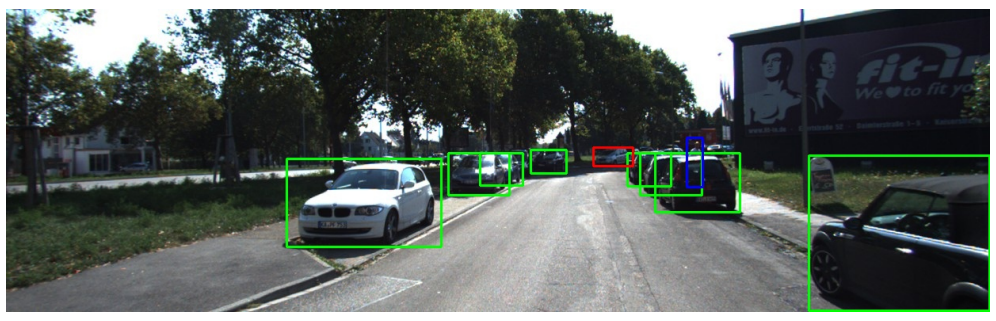

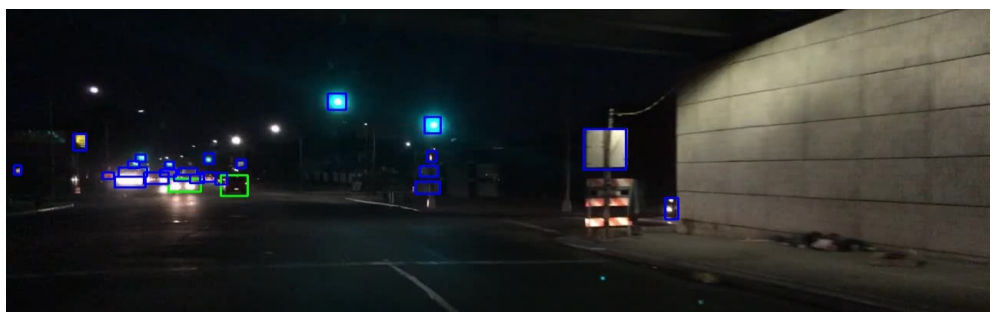

(a) Errors and misses due to small objects and occlusions. (b) Misses occur due to low-light scenarios.

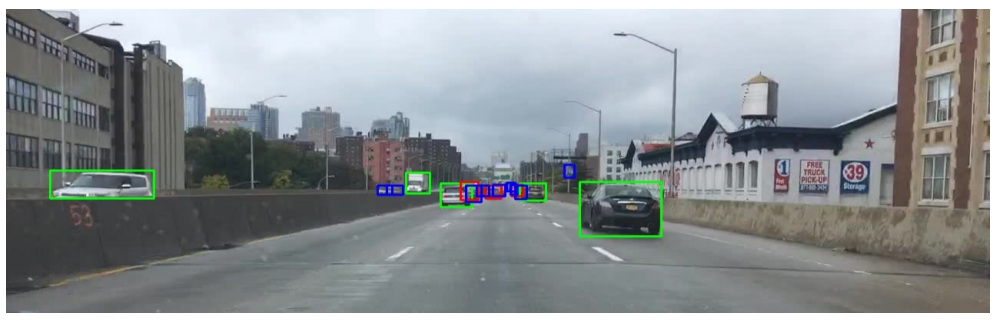

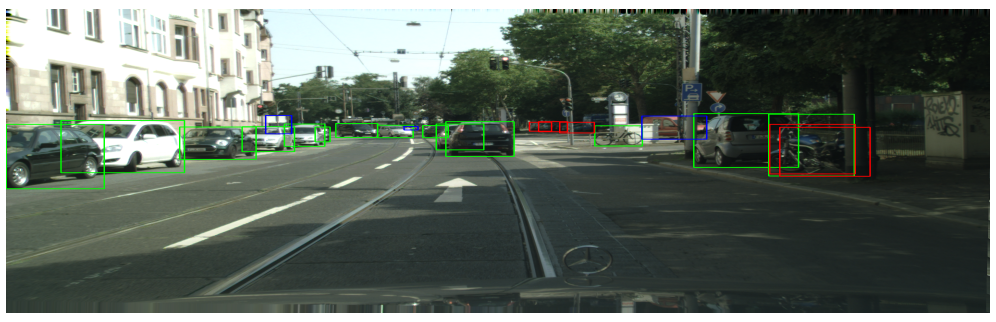

(c) Errors and misses caused by dense objects. (d) Errors and misses caused by low-quality objects.

**Supplementary Fig 1**: Visualizing autonomous driving images include on-road cars captured by a camera mounted upon a driving car which have more occlusions, dense small objects, motion blur, various types of noise and extensive scale variations. Boxes in these figures, green denotes detections, blue represents misses, and red signifies errors. Images from the KITTI Vision Benchmark Suite are used with permission and made available under the CC BY-NC-SA 3.0 licence (https://creativecommons.org/licenses/by-nc-sa/3.0/).

attributed to the absence of high-frequency content, aligning with findings in [100]. To address these challenges, adaptive low-pass filters are employed to suppress inconsistent high-frequency noise, while high-pass filters are utilized to preserve task-relevant high-frequency details and to sharpen boundaries, ultimately improving the fidelity and discriminative power of visual representations.

**Traffic Object Detection Networks**

In this section, we provide a concise overview of monocular vision traffic object detection methods, as our approach falls within the domain of monocular vision detection. The prevailing CNN-based object detectors in the current landscape can be categorized into two-stage [5, 109] and one-stage [7, 66] approaches. The majority of these methods employ the feature pyramid network (FPN) [53] to address the challenge of multiscale variation [110, 111] among objects. In contrast to methodologies such as those presented in [112, 113], which rely exclusively on a single feature map for detecting objects of various scales, FPN capitalizes on the hierarchical nature of convolutional neural networks (CNNs). FPN integrates low-level features that preserve details with high-level features abundant in semantics, and allocates classification and localization tasks to specific feature maps depending on the scale of objects within the images. The notable insight [114] that feature maps at lower layers exhibit strong responses to edges or instance parts underscores the necessity of enhancing a bottom-up pathway to propagate features with robust boundary information and to enrich all features with sufficient localization capability. Recent investigations [85, 111, 115] have sought to layer this feature fusion structure multiple times to facilitate extensive information exchange between high-level and low-level features. The aforementioned works represent a strategic compromise aimed at mitigating the inaccurate localization

information present in high-level feature maps and addressing the insufficient semantic context within low-level feature maps. For TOD task, numerous studies aim to address the common challenges encountered in detecting traffic objects in images and videos, including a wide range of object scales, occlusion and complex backgrounds [77, 83, 84, 87].

**Feature Fusion and Enhancement**

Feature fusion combines fine-grained, high-resolution features from lower layers with coarse, high-level features to integrate both detailed and semantic information [116]. In recent years, improvements to feature fusion methods have mainly been categorized into kernel-based approaches and sampling-based approaches. To integrate low- and high-resolution features, upsampling is required to restore spatial detail. Traditional methods such as nearest-neighbor and bilinear interpolation adopt fixed, handcrafted kernels determined by pixel distance. Although deconvolution [117], PixelShuffle [99], and DUpsampling [118] involve learnable parameters, their kernels become spatially invariant after training. Recent studies have emphasized the importance of dynamic upsampling. Un-pooling [119] introduces dynamic behavior by using max pooling indices to guide upsampling positions. CARAFE [120] reconstructs local decoder features in a context-aware manner. IndexNet [121] and A2U [122] utilize sub-networks to predict data-dependent kernels, yet mainly rely on encoder features. SAPA [56] and FADE [57] further incorporate both encoder and decoder information to generate adaptive kernels. These methods collectively highlight the potential of data-driven, content-aware upsampling in improving feature fusion quality.

Recent methods improve feature fusion by adjusting sampling positions to enhance spatial alignment. GUM [123] and SFNet [124] predict offsets to guide feature warping. AlignSeg [125] extends this by aligning multi-scale features with learned offsets. FaPN [126] applies deformable convolutions [16] for progressive alignment across resolutions, while IFA [127] employs implicit neural representations to infer offsets. Dysample [128] dynamically samples high-level features for content-aware upsampling.

These approaches apply spatial offsets—explicit or implicit—to better align features of different resolutions. Additionally, works such as [49], [55], [129] integrate channel attention or gating mechanisms to adaptively fuse features using context-conditioned weights. Mask2Former [130] combines deformable attention with learned offsets and weights for multi-scale fusion. Our method relates to both kernel- and sampling-based strategies but differs by explicitly defining and quantifying intra-category inconsistency and boundary displacement. The proposed AFF addresses both through feature similarity analysis, enhancing consistency and boundary precision.

**Supplementary Table 1**: Criteria for Dividing the Three Levels in KITTI Dataset

| Criteria | Easy | Moderate | Hard |
|---|---|---|---|
| Min Height (pixel) | 40 | 25 | 25 |
| Occlusion | Fully Visible | Partially Occluded | Difficult to see |
| Truncation (%) | 15 | 30 | 50 |

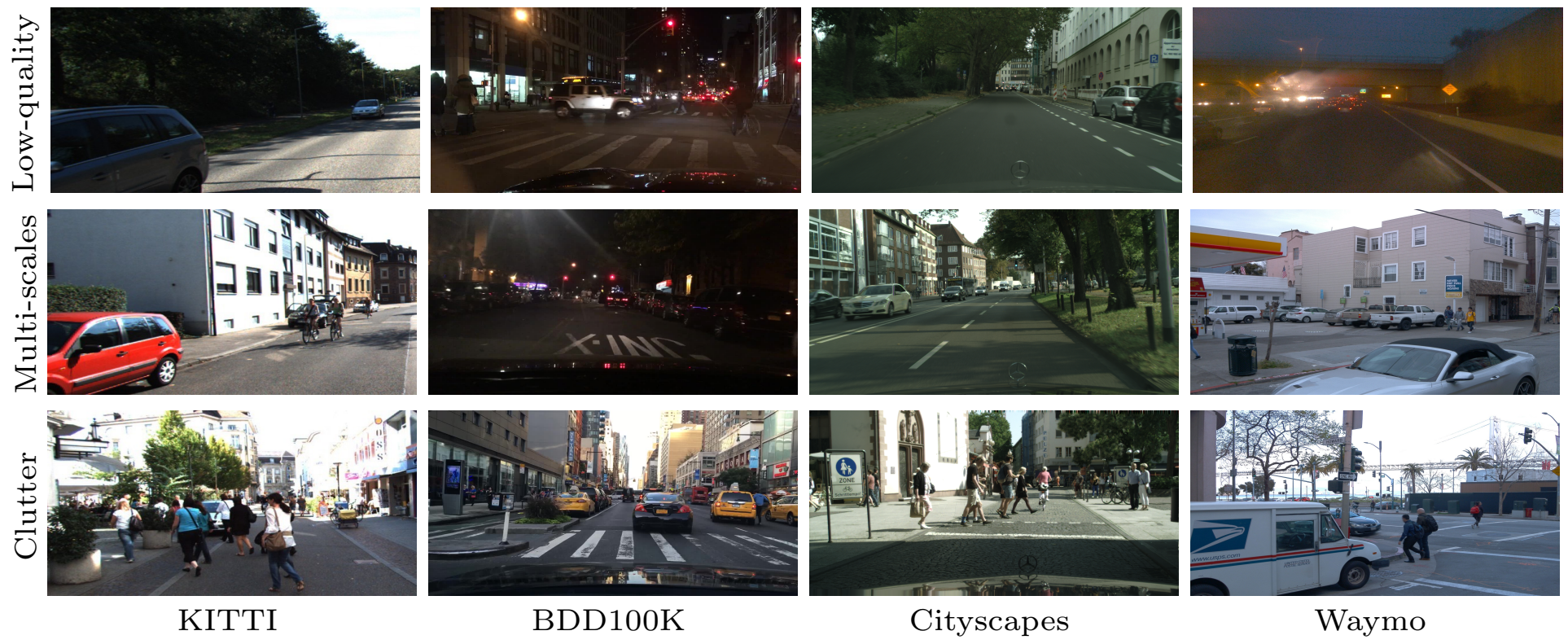


**Supplementary Fig 2**: Representative samples from KITTI, BDD100K, Cityscapes, and Waymo datasets, showing diverse traffic scenes and environmental conditions. Images from the KITTI Vision Benchmark Suite are used with permission and made available under the CC BY-NC-SA 3.0 licence (https://creativecommons.org/licenses/by-nc-sa/3.0/).

**Supplementary Table 2**: Statistical significance analysis on KITTI (5 runs, paired t-test).

| Method | Mean mAP (%) | Best (%) | Std | 95% CI |
|---|---|---|---|---|
| YOLOv11 | 88.2 | 88.4 | 0.16 | 88.1–88.4 |
| RT-DETR | 88.3 | 88.5 | 0.19 | 88.1–88.5 |
| **Ours** | **90.5** | **90.9** | **0.25** | **90.3–90.7** |
| Significance (vs. Ours) | | | | |
| YOLOv11 | | +2.3 | | **p = 0.003** |
| RT-DETR | | +2.2 | | **p = 0.001** |

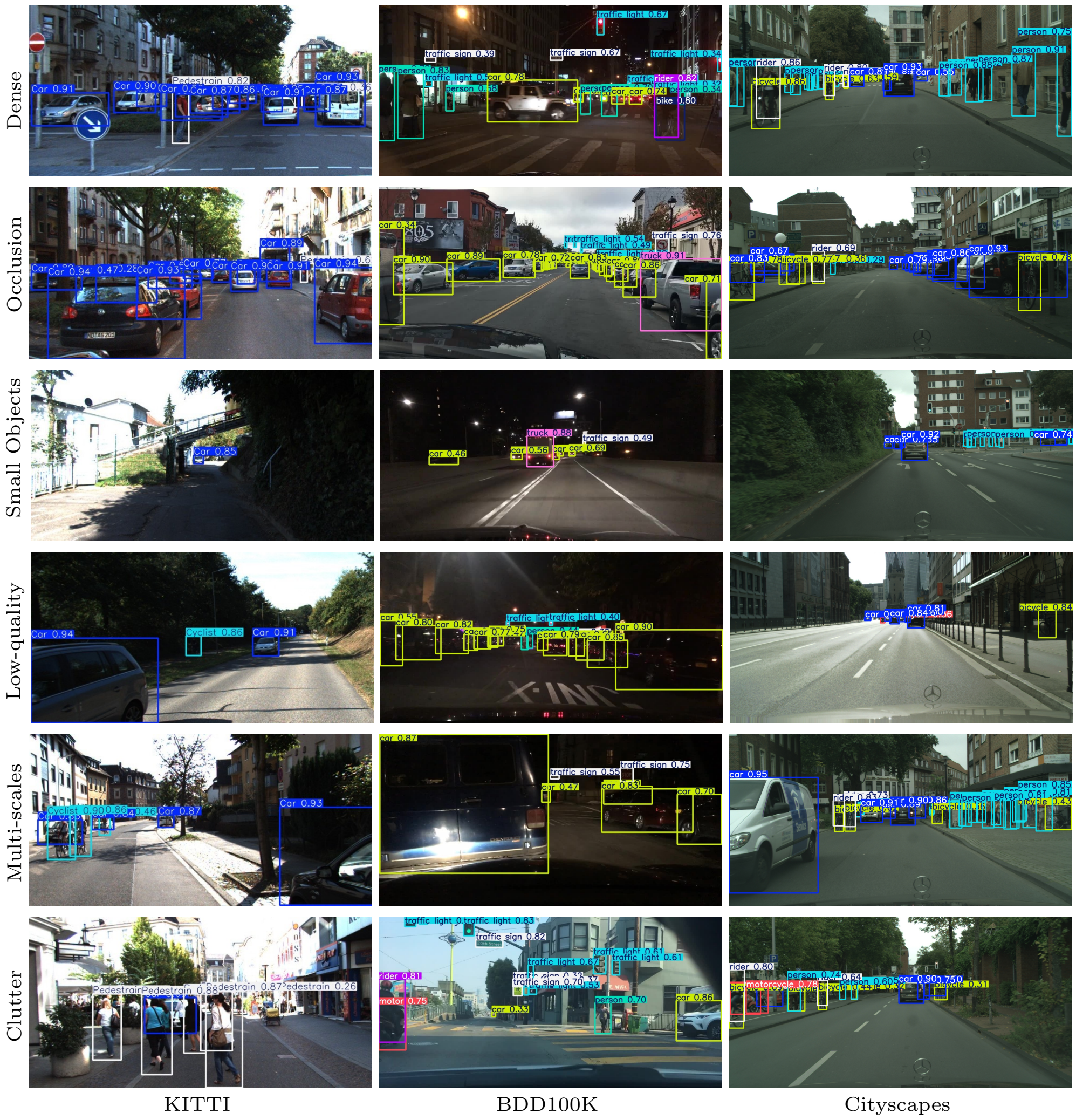


**Supplementary Fig 3**: Representative detection results of FAD on the KITTI, BDD100K and Cityscapes datasets. FAD in accurately detecting objects of traffic scenario, such as dense, occlusion, small objects, low-quality, multi-scale and clutter. Since the images we selected contain different scale objects, zooming in on the page gives a better visual effect. Images from the KITTI Vision Benchmark Suite are used with permission and made available under the CC BY-NC-SA 3.0 licence (https://creativecommons.org/licenses/by-nc-sa/3.0/).

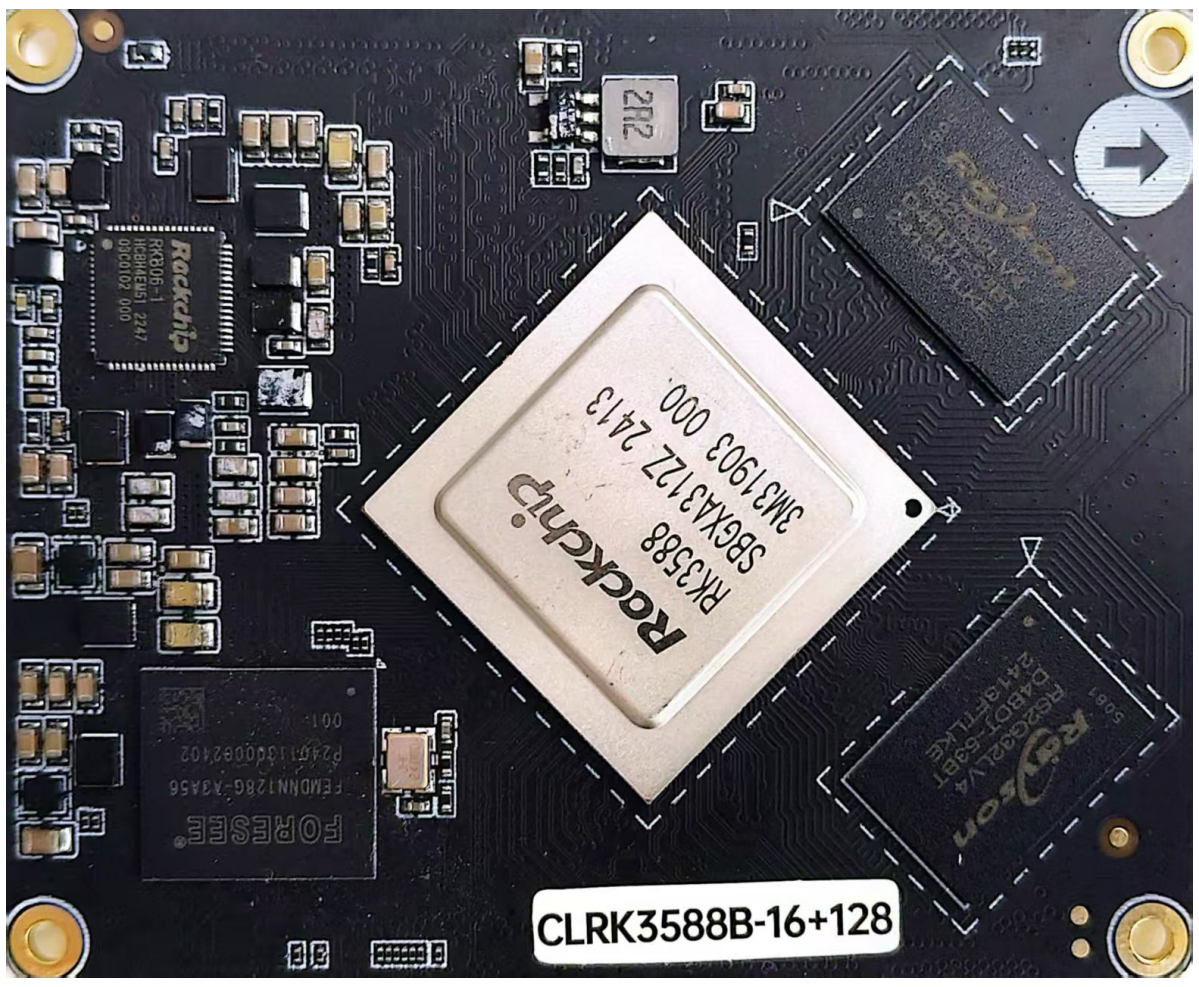


| Component | Specification |
|---|---|
| CPU | 4× Cortex-A76 (2.4 GHz) + 4× Cortex-A55 (1.8 GHz) |
| GPU | Mali-G610 MP4, up to 800 MHz |
| NPU | 6 TOPS (AI inference acceleration) |
| Memory | 8 GB LPDDR4/4X |
| Storage | 32 GB eMMC / UFS 2.1 |
| Power Consumption | Typical 10 W, Max 15 W |

**Supplementary Fig 4**: Schematic layout and specifications of the RK3588 in-vehicle hardware platform. The module integrates CPU, GPU, NPU, and memory units for efficient on-board deep learning inference.

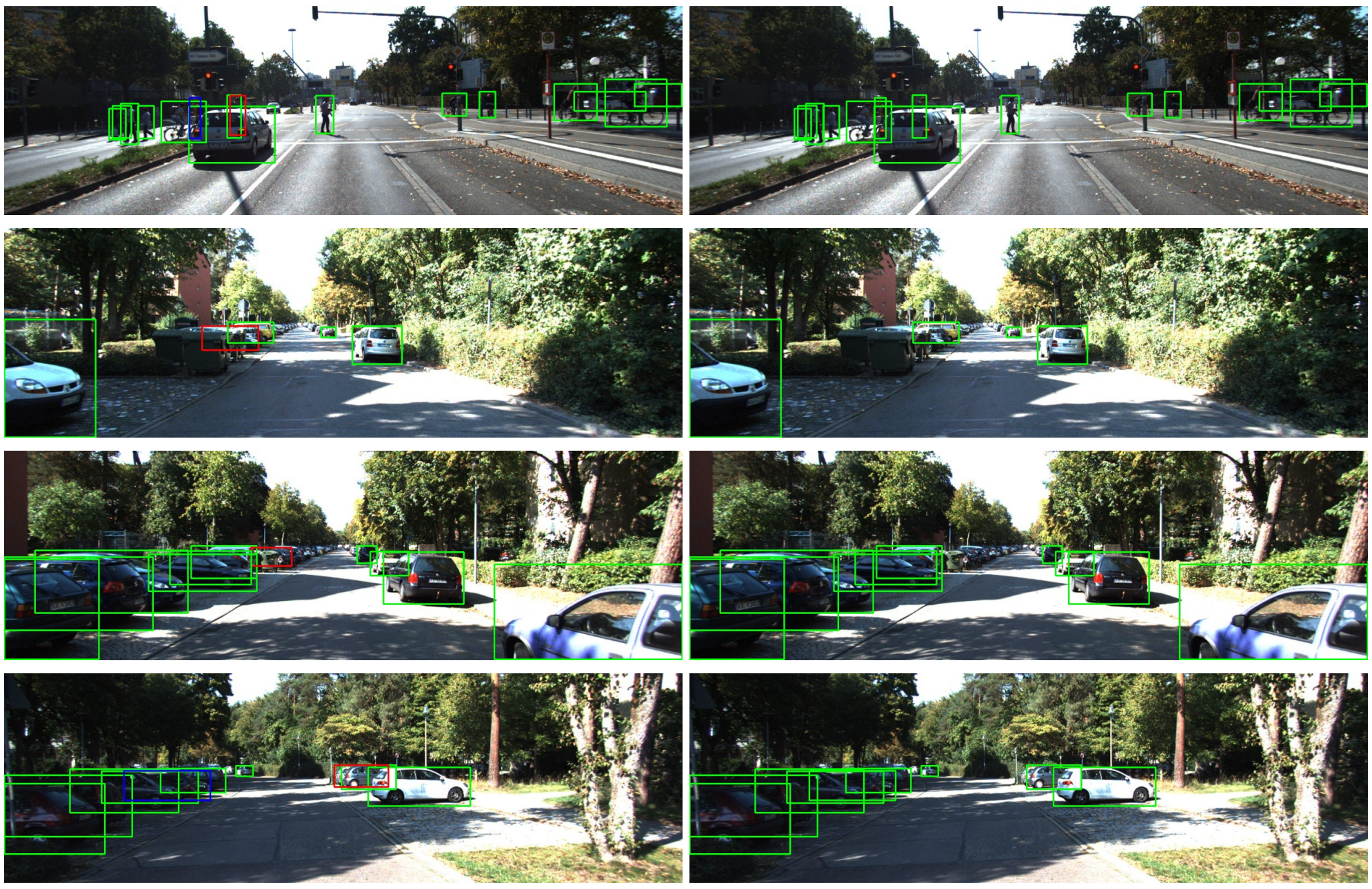

Baseline FAD

**Supplementary Fig 5**: Representative enhancement results of FAD on the KITTI dataset are displayed. FAD notably excels in reducing false positives and missed detections, as well as enhancing the detection efficiency of occluded and small-scale objects. In the figure, correctly detected objects are denoted by green boxes, missed detections are represented by blue boxes, and false positives are indicated by red boxes. Given the variety of object scales in the selected images, zooming in on the page would offer a more detailed visual representation. Images from the KITTI Vision Benchmark Suite are used with permission and made available under the CC BY-NC-SA 3.0 licence (https://creativecommons.org/licenses/by-nc-sa/3.0/).

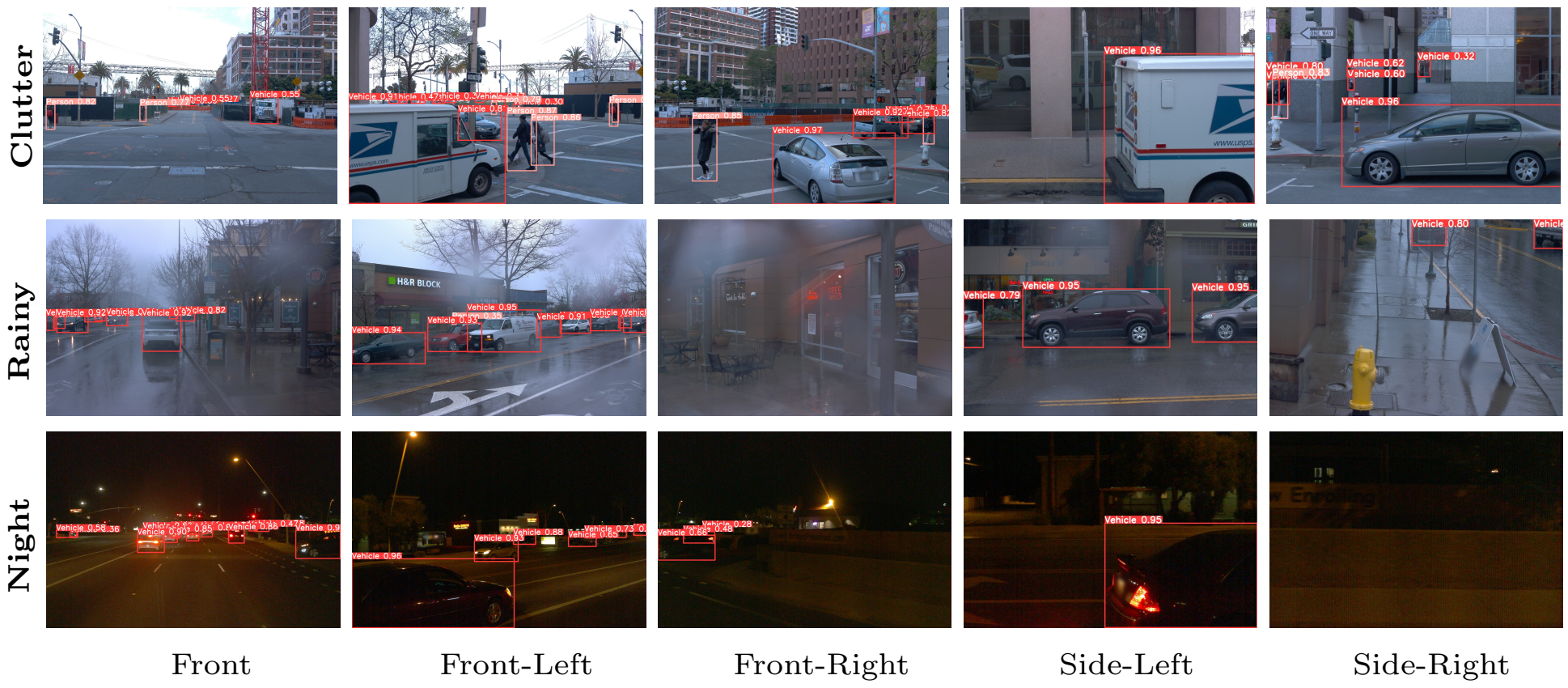


**Supplementary Fig 6**: Representative detection results of FAD on the Waymo 2D dataset, illustrating various traffic scenarios captured from 5 on-board cameras, including front, front-left, front-right, side-left, and side-right views.
