# Supplementary material for: Frequency-oriented adaptive real-time object detector for cluttered traffic scenes
Source: Nat Commun. 2026 Sep 14;17:9787. doi: 10.1038/s41467-026-76346-1 (PMC13575256; doi:10.1038/s41467-026-76346-1)
Supplement: Supplementary file 2 [file 41467_2026_76346_MOESM2_ESM.pdf]

nature portfolio

Peer Review File

Frequency-oriented Adaptive Real-Time Object Detector for Cluttered Traffic Scenes

Corresponding Author: Dr Ziqi Li

This file contains all reviewer reports in order by version, followed by all author rebuttals in order by version.

Version 0:

Reviewer comments:

Reviewer #1

(Remarks to the Author)
The proposed Frequency-Oriented Adaptive Detector (FAD) enhances vehicle-mounted intelligent traffic object detection by effectively integrating high- and low-frequency information to improve texture and semantic features of multi-scale objects in complex scenarios, while maintaining low computational overhead. It introduces Frequency Dynamic Convolution (FDC), a lightweight backbone that adaptively adjusts dilated receptive fields in the frequency domain by decomposing and recalibrating convolution weights into high- and low-frequency components. Additionally, the Adaptive Frequency-Oriented Fusion (AFF) framework reorganizes frequency features across scales to balance object details, fine boundaries, and deep semantics, addressing feature inconsistencies. Experiments show best accuracy, and robustness across multiple datasets. I recommend some adjustments to further improve the presentation of the results and to remove some ambiguity before recommending this
paper for acceptance:
1. The dataset used in this method need more information and give some sample image data.
2. The quantitative and qualitative results are not enough. The authors should include a separate section with resulting images to make the readers easy
understanding.
3. How to balance the computational cost and detection accuracy improvement in this method?
4. There are some issues with writing in various parts of the article. Hence, the writing of this paper needs further polishing.
5. I suggest that the authors discuss the limitations of the proposed method. In fact, any method has its own limitations and shortcomings.
6. Overall, the literature review should be improved. Without discussing the previous works, the novelty of the paper cannot be assessed properly. Discuss the following papers in related work section, Hybrid multi-attention transformer for robust video object detection, Learning dynamic spatial-temporal regularized correlation filter tracking with response deviation suppression via multi-feature fusion, Learning disruptor-suppressed response variation-aware multi-regularized correlation filter for visual tracking, Adaptive spatially regularized target attribute-aware background suppressed deep correlation filter for object tracking

Reviewer #2

(Remarks to the Author)
The authors propose a frequency-oriented adaptive detector (FAD) for vehicle-mounted intelligent traffic object detection. The FAD effectively integrates high- and low-frequency information to enhance the texture and semantic features of multi-scale objects in complex traffic scenarios, all while maintaining minimal computational overhead. Experimental results demonstrate that the FAD outperforms state-of-the-art detectors, obtaining the mean average precision (mAP) of 90.9%, 53.7% and 50.2% on KITTI, BDD100K and Cityscapes datasets with the parameter size of only 8.77MB, respectively.
However,the manuscript would be significantly improved by addressing the following issues:
1. The authors emphasize in the abstract that the research motivation is to address perception challenges on in-vehicle platforms. However, the experiments were conducted using a consumer-grade RTX 4070-Ti workstation. My key question is: Can this workstation be considered a compliant automotive-grade hardware platform? If not, the authors should clarify the automotive-grade compliance of the experimental platform, or modify the claims of innovation to accurately reflect the research scope.

2. The authors have conducted validation experiments on the KITTI, BDD100K, and Cityscapes datasets. To further strengthen the robustness of the findings, it is recommended to perform additional validation on larger-scale datasets such as Waymo and nuScenes.
3. While the current manuscript demonstrates algorithmic validation across three standard datasets (KITTI, BDD100K, Cityscapes), we recommend the authors strengthen the application perspective by: platform deployment details, real-world performance, and system integration.

Reviewer #3

(Remarks to the Author)
The paper proposes a frequency-oriented adaptive detector (FAD) for real-time traffic object detection. The key contributions are (1) Frequency Dynamic Convolution (FDC), which decomposes convolution kernels into high- and low-frequency components and adaptively adjusts dilation rates; (2) Adaptive Frequency-oriented Fusion (AFF), which applies low-pass/high-pass adaptive filters, offset correction, and cross-scale contribution allocation to better combine features at different levels. The authors evaluate the method on KITTI, BDD100K, and Cityscapes, claiming superior performance over existing detectors with a lightweight computational cost.

I found this paper has the following strengths: firstly, this work focuses on a practically relevant problem (lightweight object detection in autonomous driving under cluttered scenes). Secondly, both the FDC and AFF modules are described in detail, with equations and visualizations. Thirdly, experiments are conducted on three widely used benchmarks with ablation studies.

However, the proposed ideas largely combine existing concepts (frequency-domain analysis, dilated convolution adaptation, feature pyramid fusion). FDC essentially resembles prior works on deformable/dynamic convolutions or spectral-aware convolution (e.g., frequency-aware CNNs, adaptive filtering) with minimal new insights. AFF is essentially a re-implementation of feature alignment + multi-scale fusion strategies (similar to CARAFE, FaPN, AlignSeg, etc.), only rephrased with frequency terminology. So, the “frequency” perspective is more a reinterpretation rather than a fundamentally new method. The claimed novelty is incremental and not sufficient for the NC.

In addition, baselines are somewhat outdated: comparisons focus on YOLOv3, YOLOX, YOLOv8, etc., but do not include more recent lightweight detectors or transformer-based architectures that dominate current literature. And most comparisons are from IEEE TITS, not from top-tier journals like NC. And reported improvements (1–3% mAP) are marginal and within the range of hyperparameter tuning or stronger data augmentation. No statistical significance analysis is provided. And this work only presents simulation results, did not conduct any real-world experiments. Therefore, the paper presents an application-driven detector design but lacks substantial novelty, rigorous justification, and convincing experimental validation. The main contributions are incremental improvements over existing adaptive convolution and feature fusion strategies, with limited theoretical depth and modest empirical gains. Writing is verbose, and the comparisons do not cover the true state of the art.

Reviewer #4

(Remarks to the Author)
The paper proposes a new object detector, called Frequency-oriented Adaptive Detector (FAD), designed for traffic scenes in autonomous driving. The key idea is to exploit frequency-domain information to improve detection of small, occluded, and noisy objects in cluttered environments. The method combines two main contributions: Frequency Dynamic Convolution (FDC), which adaptively adjusts dilation rates and kernel weights to balance receptive field and frequency bandwidth, and Adaptive Frequency-oriented Fusion (AFF), which reorganizes high- and low-frequency features across multiple scales to improve consistency and boundary precision. Experiments on KITTI, BDD100K, and Cityscapes show that FAD achieves state-of-the-art accuracy with very small model size (8.77M parameters), demonstrating both efficiency and robustness across datasets.

The main strengths of the paper are the originality of combining frequency-domain analysis with convolution and feature fusion, and the strong empirical performance across the three aforementioned benchmarks. The introduction of FDC is well-motivated, as it provides a principled way to avoid the frequency loss typically caused by dilated convolution. The experimental section is well made: ablations show the incremental contribution of each component, comparisons demonstrate superiority over recent lightweight and heavy detectors. Another strength is the efficiency of the model: despite introducing new modules, the parameter size remains small and inference speed is high, which is crucial for deployment on automotive hardware.

On the other hand, the paper has some weaknesses. First, while the proposed modules are conceptually novel, the technical description is a bit complex, also with many hand-crafted design choices (e.g., decomposition of frequency bands, the use of adaptive filters and offsets) that may limit reproducibility and generalization. The reliance on Fourier-domain interpretations is also not always fully justified. Second, the comparison with state-of-the-art detectors does not always ensure fairness: some baselines are outdated (e.g., SSD, YOLOv3) or not tuned to the same lightweight constraints, while very recent strong baselines (e.g., YOLOv9 or 10, transformer-based detectors) are missing. Third, although the model is lightweight, the reported FLOPs (39.7G) are relatively high compared to other detectors, raising doubts about deployment feasibility on resource-constrained embedded systems.

In summary, the paper addresses an important problem with an original frequency-based perspective, shows strong empirical results and efficiency, but suffers from presentation complexity, incomplete comparisons, and some over-claims.

Version 1:

Reviewer comments:

Reviewer #1

(Remarks to the Author)
The authors have addressed all of my comments. This paper can be accepted for publication.

Reviewer #2

(Remarks to the Author)
The authors have addressed the concerns raised by the reviewers. I have no further comments.

Reviewer #3

(Remarks to the Author)
I would like to thank the authors for their detailed and comprehensive response to my previous comments. I sincerely appreciate the significant effort put into the revision, particularly the inclusion of additional experiments on the large-scale Waymo 2D dataset and the deployment validation on the RK3588 in-vehicle platform. The inclusion of recent state-of-the-art baselines, such as YOLOv10, YOLOv11, and RT-DETR, has significantly strengthened the fairness and contemporaneity of the performance evaluation. The experimental validation is now much more robust and demonstrates the practical effectiveness of the FAD framework in real-world scenarios.

However, despite these improvements, there are still critical issues that need to be addressed before I can fully recommend this manuscript for publication in Nature Communications.

1. Statistical Significance Analysis (Unresolved): In my initial review, I explicitly requested a statistical significance analysis to ensure that the reported improvements (1-3% mAP) are statistically meaningful and not merely the result of stochastic variance. While the revised manuscript and response emphasize ``consistent performance'' across datasets, formal statistical tests (e.g., t-tests, p-values, or confidence intervals across multiple runs) are still missing. Given that the performance gains are relatively marginal in some cases, providing statistical evidence is non-negotiable for a high-impact journal to confirm the reliability of the results. Please include this analysis in the final version.

2. Novelty and Theoretical Contribution: I appreciate the authors' honesty in acknowledging that the proposed modules (frequency-domain analysis, dilated convolution, etc.) conceptually align with prior works. The authors argue that the novelty lies in the ``reinterpretation and integration'' of these concepts through a frequency-based perspective. While I agree that the engineering integration is executed well and the results are solid, my concern regarding the work being an ``incremental improvement'' remains. To meet the high novelty bar of Nature Communications, the paper needs to elevate itself beyond a successful ``application-driven design''. I strongly suggest the authors strengthen the theoretical explanation of why this frequency-oriented approach is superior. Rather than just describing how it works (reinterpretation), please provide deeper theoretical insights or mathematical justifications explaining why the frequency domain is the optimal space for solving the specific problems of feature alignment and boundary precision in this context.

I am satisfied with the experimental breadth and the engineering robustness of the revised manuscript. However, to support the claims of superiority and justify publication in this venue, it is essential to: Add the missing statistical significance analysis. And deepen the theoretical discussion to demonstrate a more fundamental contribution beyond engineering integration.

I look forward to seeing these final polishings.

Version 2:

Reviewer comments:

Reviewer #3

(Remarks to the Author)
Thanks for the detailed responses. The authors have addressed all my concerns.

Open Access This Peer Review File is licensed under a Creative Commons Attribution 4.0 International License, which permits use, sharing, adaptation, distribution and reproduction in any medium or format, as long as you give appropriate credit to the original author(s) and the source, provide a link to the Creative Commons license, and indicate if changes were made.
In cases where reviewers are anonymous, credit should be given to 'Anonymous Referee' and the source.
The images or other third party material in this Peer Review File are included in the article's Creative Commons license, unless indicated otherwise in a credit line to the material. If material is not included in the article's Creative Commons license and your intended use is not permitted by statutory regulation or exceeds the permitted use, you will need to obtain permission directly from the copyright holder.
To view a copy of this license, visit https://creativecommons.org/licenses/by/4.0/

Detailed Response to Reviewers

Thank you for your insightful comments on our manuscript. We sincerely appreciate the time and effort you have devoted to providing valuable feedback and constructive suggestions. In response to your comments, we have revised the manuscript accordingly. A point-by-point response is provided below, detailing the modifications made. We hope that our revisions address your concerns and meet your expectations.

Thank you once again for your kind review~

Contents

Response to Reviewer 1(Click to jump) 1
Response to Reviewer 2(Click to jump) 12
Response to Reviewer 3(Click to jump) 19
Response to Reviewer 4(Click to jump) 31
At the End 43

Response to Reviewer 1

Comments

The proposed Frequency-Oriented Adaptive Detector (FAD) enhances vehicle-mounted intelligent traffic object detection by effectively integrating high- and low-frequency information to improve texture and semantic features of multi-scale objects in complex scenarios, while maintaining low computational overhead. It introduces Frequency Dynamic Convolution (FDC), a lightweight backbone that adaptively adjusts dilated receptive fields in the frequency domain by decomposing and recalibrating convolution weights into high- and low-frequency components. Additionally, the Adaptive Frequency-Oriented Fusion (AFF) framework reorganizes frequency features across scales to balance object details, fine boundaries, and deep semantics, addressing feature inconsistencies. Experiments show best accuracy and robustness across multiple datasets.

I recommend some adjustments to further improve the presentation of the results and to remove some ambiguity before recommending this paper for acceptance:

1. The dataset used in this method needs more information and sample image data should be provided.
2. The quantitative and qualitative results are not enough. The authors should include a separate section with resulting images to facilitate reader understanding.
3. How to balance the computational cost and detection accuracy improvement in this method?
4. There are some issues with writing in various parts of the article. Hence, the writing of this paper needs further polishing.
5. I suggest that the authors discuss the limitations of the proposed method. In fact, any method has its own limitations and shortcomings.
6. Overall, the literature review should be improved. Without discussing the previous works, the novelty of the paper cannot be assessed properly. The following papers should be discussed in the related work section:
   - Hybrid multi-attention transformer for robust video object detection
   - Learning dynamic spatial-temporal regularized correlation filter tracking with response deviation suppression via multi-feature fusion
   - Learning disruptor-suppressed response variation-aware multi-regularized correlation filter for visual tracking
   - Adaptive spatially regularized target attribute-aware background suppressed deep correlation filter for object tracking

Reply: Thank you for your insightful comments on our manuscript. We sincerely appreciate the time and effort you have devoted to providing valuable feedback and constructive suggestions. In response to your comments, we have revised the manuscript accordingly. A point-by-point response is provided below, detailing the modifications made. We hope that our revisions address your concerns and meet your expectations.

Thank you once again for your kind review~

1. The dataset used in this method need more information and give some sample image data.

Reply: Thank you for your constructive suggestions. Your feedback has been extremely helpful in improving the quality and clarity of our manuscript. We sincerely apologize for the lack of rigor and clarity in original manuscript. In the revised manuscript, we have added more details about the dataset used in our experiments, including the data source, the number of images, the annotation format, and the distribution of categories. In addition, we have included representative sample images in Figure 7 in the revised manuscript to help readers better understand the dataset.

Since the revisions are extensive, we summarize them step by step for clarity. We truly appreciate your valuable comments, which have significantly improved our manuscript. All changes are highlighted in purple in the revised version, and the corresponding updated content is presented in the green boxes below with page numbers indicated.

Revised Content in the Manuscript

4.2 Datasets

1) KITTI dataset[1] comprises 7481 training images and 7518 test images, with a total of 51867 objects in the training set, of which cars represent 28742. The main challenge in KITTI car detection lies in the abundance of small-sized cars (height < 40 pixels) and occluded instances. Due to the unavailability of ground truth annotations for the KITTI test set, divided 3,682 images and 3,799 images into training and validation, respectively, for our framework analysis. In the KITTI dataset, performance evaluation was conducted across three difficulty levels: easy, moderate, and hard. The criteria for categorizing these levels and accounting for occlusion scenarios are detailed in Table 1.

Table 1: Criteria for Dividing the Three Levels in KITTI Dataset

Criteria	Easy	Moderate	Hard
Min Height (pixel)	40	25	25
Occlusion	Fully Visible	Partially Occluded	Difficult to see
Truncation (%)	15	30	50

Low-quality

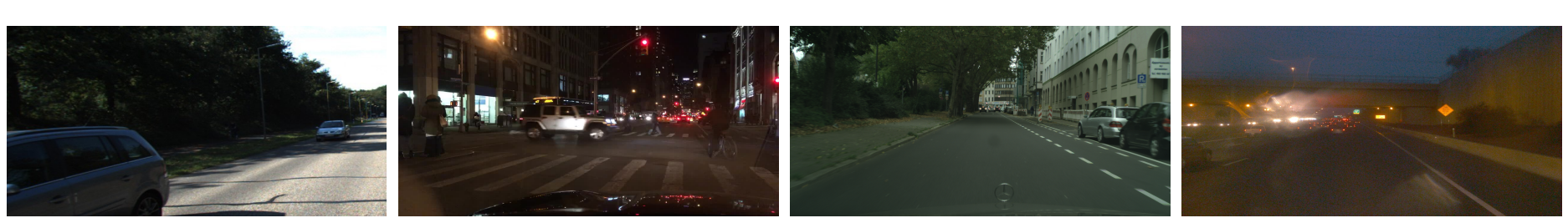

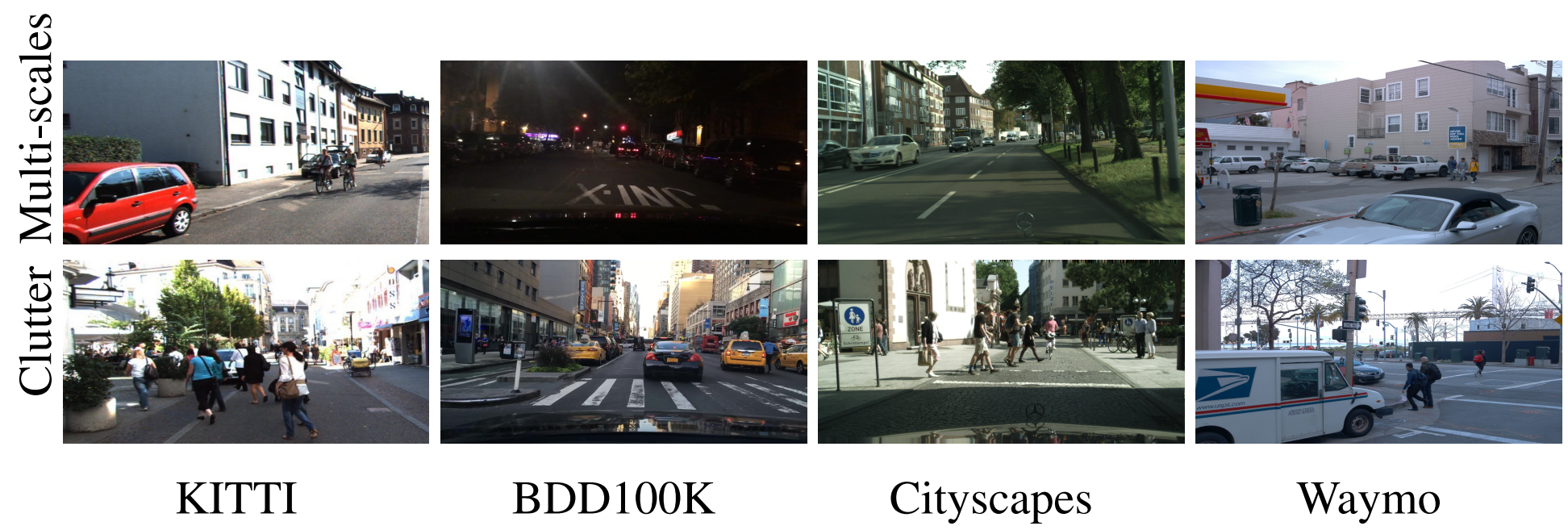

Figure 7: Representative samples from KITTI, BDD100K, Cityscapes, and Waymo datasets, showing diverse traffic scenes and environmental conditions.

2) BDD100K dataset[2] comprises 100,000 video sequences captured by a car camera, each with a resolution of 1280 × 720 pixels. These sequences cover various times of day, driving scenarios, and weather conditions, and include 10 classes: car, bus, person, bike, truck, motor, train, rider, traffic sign, and traffic light. The dataset contains images with diverse illumination conditions and object sizes. The dataset is partitioned into training, validation, and test sets in a ratio of 7:1:2. Our model is trained using the training and validation data and evaluated on the test set to assess its performance.

3) Cityscapes dataset[3] is a widely used benchmark for urban object detection, consisting of high-resolution street-view images captured in 50 European cities under diverse environmental conditions. It features densely populated urban scenes with complex backgrounds, frequent occlusions, and a wide range of object scales. Following the standard split, we use 2,975 finely annotated images for training and 500 for validation. These characteristics make Cityscapes suitable for evaluating object detection models in realistic and challenging urban environments.

4) Waymo 2D dataset[98] provides camera-based annotations for vehicles, pedestrians, and cyclists. Bounding boxes tightly enclose only the visible regions of objects without estimating occluded parts. Each object is consistently tracked across frames with occlusion states marked. Vehicles include cars and motorcycles but exclude trains and trams; pedestrians cover walking persons and those on small mobility devices; cyclists include both the rider and bicycle. This dataset offers precise, visibility-consistent labels for 2D perception and tracking tasks in autonomous driving.

Page 17-18

2. The quantitative and qualitative results are not enough. The authors should include a separate section with resulting images to make the readers easy understanding.

Reply: We thank the reviewer for this valuable suggestion. To improve the clarity and comprehensiveness of our results, we have revised the title of Section 4.4 to “Comparison Experiments and Quantitative Analysis”, and included additional comparative experiments with recent state-of-the-art methods to highlight the superiority of our approach. Furthermore, we have expanded the

ablation study by conducting detailed quantitative analyses on the three key components: FDC, AFF, and FAD. In addition, both quantitative and qualitative analyses of the receptive field transformation of FDC are presented in Fig. 8.

In Section 4.5, we have added additional zoom-in view results to provide more intuitive qualitative comparisons of FAD under various challenging scenarios in Fig. 10. Moreover, we have refined the descriptions related to the comparative visualization in Fig. 9 to help readers better understand the proposed method.

Revised Content in the Manuscript

4.5 Visualization of Detection Results

To further substantiate the effectiveness of the proposed FAD, we present a series of qualitative visualizations on the KITTI, BDD100K, Cityscapes and Waymo 2D datasets. As illustrated in Fig. 11, FAD is evaluated under six representative traffic scenarios in KITTI, exhibiting consistently strong detection performance. The model accurately localizes pedestrians partially concealed by vehicles, overlapping human instances, and distant small-scale targets, highlighting its capacity to handle complex occlusions and scale variations. Moreover, FAD achieves remarkable accuracy in identifying multi-scale, densely occluded pedestrians and vehicles, demonstrating robustness against cluttered backgrounds and severe illumination fluctuations. Quantitative comparisons in Fig. 9 further confirm FAD's superior detection accuracy relative to the baseline, with consistent visual evidence of improvement across all evaluated conditions.

Zoomed-in visualizations in Fig. 10 provide detailed comparisons between FAD and the baseline across datasets. From top to bottom, the rows correspond to KITTI, BDD100K, and Cityscapes. FAD consistently delivers more precise object localization and sharper detection boundaries, maintaining robust performance across diverse visual environments. Specifically, on KITTI, FAD markedly enhances the detection of faint cyclists, improving accuracy from 0.32 to 0.70. On BDD100K, it precisely identifies vehicles in opposing lanes, while on Cityscapes, it successfully captures distant, low-resolution pedestrians. Collectively, these results demonstrate that FAD achieves stable and reliable detection performance across a wide range of challenging traffic scenarios, confirming its strong generalization capability in real-world environments.

Furthermore, by conducting qualitative comparisons between FAD and the baseline, we provide an intuitive illustration of FAD's improvements in mitigating both missed and false detections. Green boxes indicate correct detections, blue boxes denote missed objects, and red boxes correspond to false positives. FAD enhances object feature representation via frequency-guided feature extraction and adaptive fusion, substantially reducing both missed detections and false positives. This approach improves occlusion handling while preserving robust performance on small and distant targets, such as pedestrians and vehicles.

For BDD100K, as depicted in Fig. 11, FAD demonstrates outstanding performance across diverse

environmental conditions. FAD achieves accurate multi-scale object detection across diverse traffic scenarios under both daytime and low-light conditions. As shown in the third-row subfigure, FAD accurately detects multi-scale occluded objects and low-quality small targets caused by perspective distortion from the onboard camera viewpoint. Visualization results on the BDD100K dataset further demonstrate its state-of-the-art performance.

For Cityscapes, which emphasizes dense urban environments characterized by cluttered scenes, occlusions, and small objects, FAD demonstrates accurate detection of occluded and small targets, , as further illustrated in Fig. 11. Its consistent performance across datasets underscores the effectiveness of our design in addressing the challenges posed by dense layouts and scale variations inherent in urban traffic scenarios.

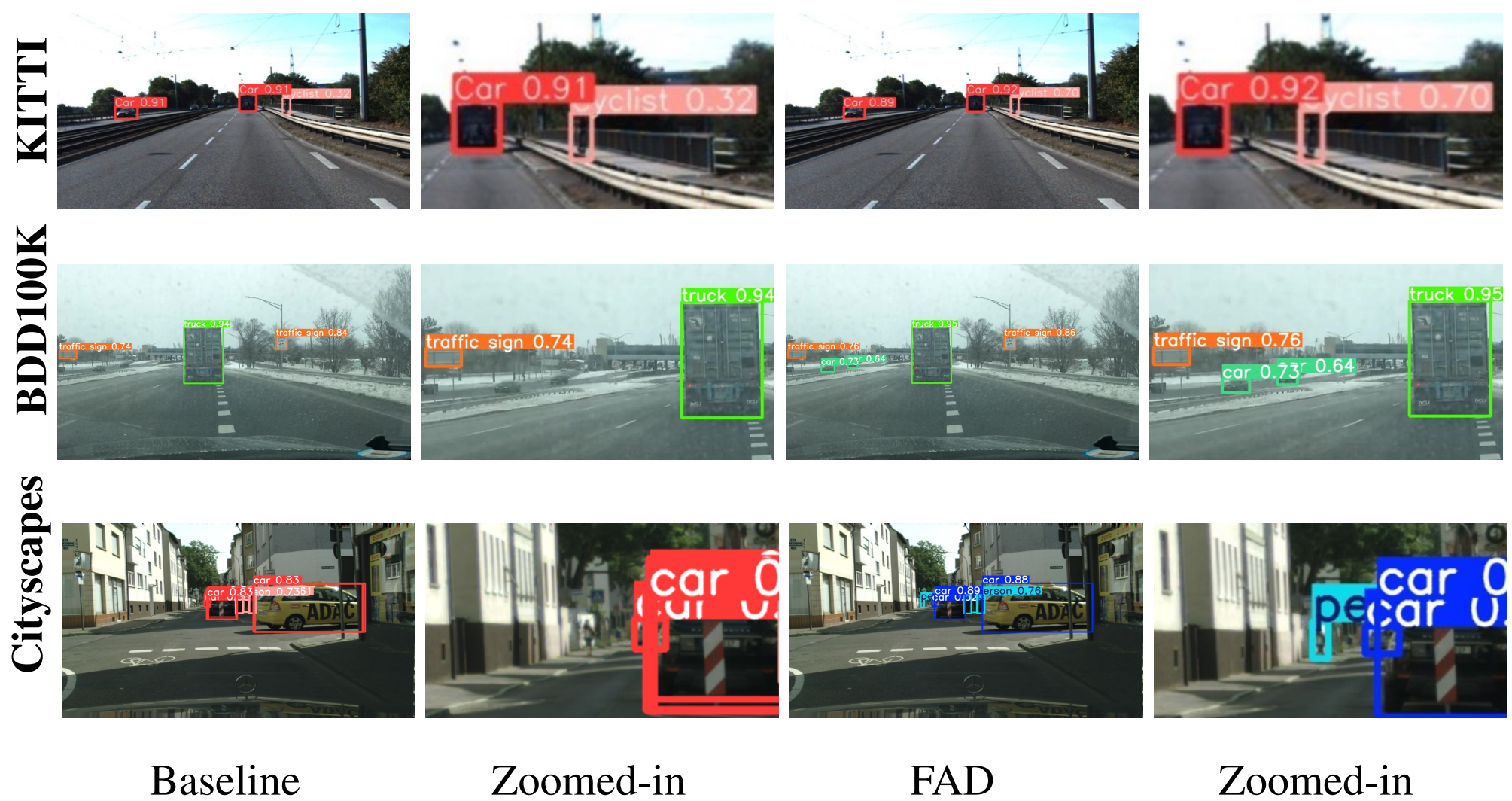

Figure 10: Representative zoom-in view results of FAD on the KITTI, BDD100K and Cityscapes datasets. Since the images we selected contain different scale targets, zooming in on the page gives a better visual effect.

3. How to balance the computational cost and detection accuracy improvement in this method?

Reply: **3. How to balance the computational cost and detection accuracy improvement in this method?**

Response: We sincerely thank the reviewer for this insightful question. Our proposed Frequency-oriented Adaptive Detector (FAD) is designed to achieve an optimal balance between computational efficiency and detection accuracy. To this end, we introduce two lightweight yet effective components, Frequency Dynamic Convolution (FDC) and Adaptive Frequency-oriented Fusion (AFF), which enhance the model's feature extraction and fusion capabilities while maintaining low computational cost.

FDC employs adaptive dilation rates, adaptive kernels, and a frequency selection strategy to expand the receptive field efficiently without introducing additional heavy convolutional operations.

AFF adopts a low-pass adaptive filter (LAF) generator, an offset generator, a high-pass adaptive filter (HAF) generator, and a cross-scale contribution allocation (CCA) mechanism to realize efficient frequency-domain guided multi-scale fusion. These designs allow the model to effectively integrate both high- and low-frequency information, thereby improving texture and semantic representations while keeping the computational overhead minimal.

As shown in Table 4, our FAD achieves higher 90.9% mAP compared with the baseline, while maintaining 39.7 GFLOPs and parameter count. Although the proposed FAD introduces a slight increase in computational cost (measured in GFLOPs), the corresponding improvement in detection accuracy demonstrates that this trade-off is worthwhile and well-balanced.

Furthermore, to verify the practical computational efficiency, we additionally conducted deployment experiments on the **RK3588 embedded hardware platform**, as described in Section 4.6. **A real-time demonstration video has been submitted as supplementary material.** The results demonstrate that FAD maintains real-time inference capability and achieves a favorable trade-off between accuracy and efficiency in real-world edge computing environments.

For your convenience, the corresponding revised content is attached in green box below, with page numbers from the manuscript shown in the lower-right corner of green box. For clarity, all modifications are also marked in purple in the revised manuscript.

Revised Content in the Manuscript

4.3 Ablation Study and Analysis

Table 2: Ablation Experiment on KITTI Dataset

Method	mAP@50	Car	Cyclist	Pedestrian	Params (M)	FLOPs (G)
Baseline	87.7	96.4	87.5	79.1	8.07	24.8
+FDC	89.3	97.1	89.0	81.7	8.15	32.6
+AFF	90.2	97.4	90.3	83.0	6.99	33.2
+FDC+AFF (FAD)	**90.9**	**97.4**	**91.8**	**83.5**	8.77	39.7

We perform the ablation experiment on KITTI benchmark to prove the effectiveness of FDC, AFF and FAD. Table 4 show the results of ablation experiments.

1) Baseline: Initially, we utilize DarkNet53 as the backbone network and PANet to extract multiscale feature maps of ADIs. The decoupling head is employed for object detection. In all experiments, uniform hyperparameters are maintained to ensure fairness in comparison. Finally, the baseline achieves 87.7% mAP on KITTI.

2) Effect of FDC: FDC is devised for frequency-oriented adaptive receptive field expansion, enhancing feature extraction capability for low-quality objects. Integrated with FDC, our model achieves a

mAP of 89.3%, indicating a 1.6% improvement. A notable 2.6% improvement was observed in the pedestrain category and a substantial 2.5% improvement in the cyclist category both prone to occlusion and forming small pixel targets in the distance. Additionally, the model's parameters (Params) saw a minor rise from 8.07M to 8.15M, while floating point operations (FLOPs) increased slightly from 24.8G to 32.6G. This highlights FDC's effectiveness in enhancing the network's comprehension of spatial-channel information, resulting in clearer object recognition and improved detection accuracy as it operates on the deep feature map.

2) Effect of AFF: AFF is engineered to effectively capture interdependencies among adjacent and non-adjacent scale features, thereby intensifying the model's emphasis on critical features while suppressing irrelevant ones. The incorporation of AFF into the baseline network led to a performance improvement of 2.5%. Additionally, integrating it into FAD resulted in a 1.6% improvement, with only a marginal increase of 0.62M parameters in the model. Furthermore, mAPs for classes cyclist and pedestrian show improvements of 2.8% and 3.9% respectively, compared to the baseline. This showcases the effectiveness of AFF in TOD, contributing to remarkable detection results with our approach.

3) Effect of FAD: FAD aims to effectively integrate high- and low-frequency information to enhance texture and semantic features, improving traffic multi- scale object detection in complex traffic scenarios while maintaining computational performance. Additionally,FAD could rectify misalignment issues between classification and localization tasks in complex environments, occlusion and small objects.

By introducing FAD, we achieve a state-of-art detection performance, with respective mAP values of 90.9% on the KITTI datasets, representing 3.2% increase over the baseline. Furthermore, the model's parameters and floating point operations amount to 8.77M and 39.7G. In conclusion, FAD demonstrates competitive performance on ADIs and facilitates the model in achieving optimal results.

4) Receptive Field: Prior work highlights the critical role of large receptive fields in TOD. Unlike conventional dilated DarkNet, which uses a fixed global dilation rate, FDC leverages the adaptive dilation rate strategy to dynamically adjust dilation rates, achieving broader receptive field coverage. As visualized in Fig. 13, FDC enhances this adaptability by prioritizing informative frequency components in feature maps, systematically increasing the average dilation rate and expanding the effective receptive field.

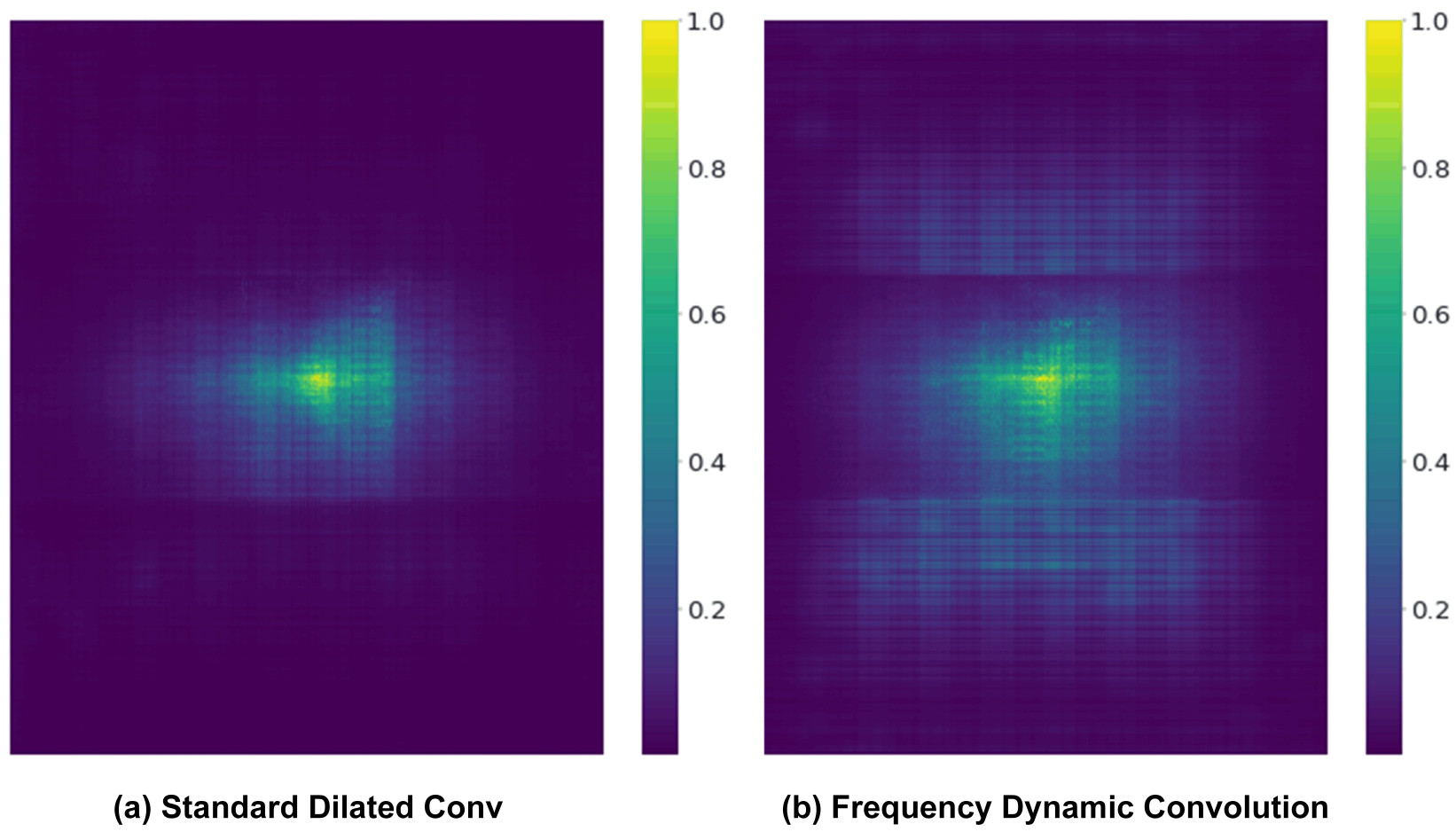

Conv Type	Standard Dilated Conv	FDC
Receptive Field	441	1100

Figure 8: Visualization of Theoretical Receptive Field Analysis

Page 18

4. There are some issues with writing in various parts of the article. Hence, the writing of this paper needs further polishing.

Reply:

We sincerely thank the reviewer for this valuable comment. We have carefully polished the entire manuscript to improve the clarity, readability, and overall presentation quality. Grammatical errors and awkward expressions have been corrected, and the writing has been refined to ensure smoother logical flow and more precise technical descriptions throughout the paper.

5. I suggest that the authors discuss the limitations of the proposed method. In fact, any method has its own limitations and shortcomings.

Reply: We appreciate the reviewer's insightful suggestion. In response, we have added a detailed discussion of the limitations of our proposed method in **Section 5 Conclusion**. Specifically, we describe potential constraints such as the sensitivity of frequency-domain operations to extreme lighting conditions and the dependency on computational resources for real-time deployment. These additions help provide a more comprehensive and balanced perspective on our work.

Revised Content in the Manuscript

5 Limitations

Despite its demonstrated effectiveness, the proposed FAD framework has certain limitations. Its performance is contingent on the quality of frequency-domain feature decomposition, which may degrade under extreme lighting conditions or adverse weather. Moreover, frequency-domain guided operations inherently incur higher computational costs in terms of GFLOPs, potentially constraining deployment on ultra-low-power edge devices.

6 Conclusion

FAD is a novel one-stage object detector designed to enhance traffic object detection performance through frequency-guided adaptive feature extraction and fusion, while maintaining a lightweight architecture. FAD, aiming at frequency-domain adaptive dilated receptive fields and balancing effective bandwidth with lightweight design and compact model size, effectively facilitates high-performance object detection tasks on automotive platforms. Additionally, AFF, tailored for TOD, adaptively reorganizes high- and low-frequency features across both adjacent and non-adjacent scales, balancing object details, fine boundaries, and deep semantic features, aiming to bridge the gap caused by feature inconsistencies. In comparative experiments with consistent dataset settings across various detection methods, FAD demonstrates superior performance, particularly in common traffic scenarios involving occlusions, small objects, complex backgrounds, and dense objects. In future work, we plan to integrate multimodal sensory data (e.g., LiDAR or radar) and explore lightweight frequency-domain operators to further improve robustness and efficiency while reducing redundant computations.

Page 24

6. Overall, the literature review should be improved. Without discussing the previous works, the novelty of the paper cannot be assessed properly. Discuss the following papers in related work section, Hybrid multi-attention transformer for robust video object detection, Learning dynamic spatial-temporal regularized correlation filter tracking with response deviation suppression via multi-feature fusion, Learning disruptor-suppressed response variation-aware multi-regularized correlation filter for visual tracking, Adaptive spatially regularized target attribute-aware background suppressed deep correlation filter for object tracking

Reply:

We are grateful for your suggestion and fully appreciate it. We have thoroughly read and studied the articles you mentioned.

References

[23] Moorthy, S., KS, S. S., Arthanari, S., Jeong, J. H., & Joo, Y. H. (2025). Hybrid multi-attention transformer for robust video object detection. *Engineering Applications of Artificial Intelli-*

gence, 139, 109606.

[27] Moorthy, S., & Joo, Y. H. (2023). Learning dynamic spatial-temporal regularized correlation filter tracking with response deviation suppression via multi-feature fusion. *Neural Networks, 167*, 360–379.

[28] Moorthy, S., KS, S. S., Arthanari, S., Jeong, J. H., & Joo, Y. H. (2025). Learning disruptor-suppressed response variation-aware multi-regularized correlation filter for visual tracking. *Journal of Visual Communication and Image Representation*, 104458.

[29] Arthanari, S., Moorthy, S., Jeong, J. H., & Joo, Y. H. (2025). Adaptive spatially regularized target attribute-aware background suppressed deep correlation filter for object tracking. *Signal Processing: Image Communication, 136*, 117305.

This paper addresses the domain generalization problem in medical image classification, caused by domain shifts from scanner and protocol variations. It proposes a novel episodic training framework with task augmentation based on meta-learning, which enhances training diversity and mitigates overfitting when only a few annotated domains are available.

In summary, the aforementioned methods represent interesting and novel work, and the related techniques are certainly worth studying and discussing. **To this end, we have cited all** the aforementioned papers in the Introduction and Related Work sections. For clarity, the corresponding revised content is attached in green box below, with page numbers from the manuscript shown in the lower-right corner of green box. For your convenience, all modifications are also marked in purple in the revised manuscript.

Revised Content in the Manuscript

1 Introduction

Vision-based traffic scene perception has become a prominent research area in intelligent transportation systems. It encompasses the detection, recognition, and tracking of various objects of interest. The ability to accurately detect these objects is fundamental to effective traffic scene perception. Vehicle-mounted traffic object detection (TOD) faces several unique challenges, including perspective-induced scale variation and occlusion, variations in lighting and weather conditions, suboptimal performance of lightweight networks, and the presence of low-quality and small-scale objects. Fig. 1 shows a few examples with varying complexities from the KITTI [1], BDD100K [2] and Cityscapes [3] datasets.

Currently, deep learning-based object detection algorithms are primarily classified into two main categories: two-stage algorithms and one-stage detectors. The two-stage algorithms, exemplified by Faster R-CNN [4], initially generate proposal regions from the input image, followed by classification. Despite their impressive accuracy, these methods are limited by the substantial computational requirements and

time overhead. One-stage algorithms, exemplified by SSD [5], YOLO [6, 7, 8, 9, 10, 11], and RetinaNet [12], treat detection as a regression task within a single network, offering high real-time processing capabilities. They strike a delicate balance between speed and accuracy by iteratively refining performance. However, vehicle computing platforms, with limited GPU and RAM, pose challenges for deploying object detection algorithms that rely on heavy computational and storage resources.

In pursuit of balancing vehicle-mounted platform performance and pruning, a range of model compression strategies and enhanced architectures have emerged to improve network efficiency. Regarding feature extraction capabilities, the importance of large receptive fields in scene understanding tasks has been emphasized [13, 14]. However, increasing the receptive field inevitably introduces computational costs. To this end, the effectiveness of content-adaptive characteristics has been demonstrated by various works [15, 16, 17, 18, 19, 20]. Recent vision transformers [14, 21, 22, 23] integrate attention mechanisms to predict input-adaptive attention values but come with high computational costs. By introducing intervals between filter elements at a specified dilation rate D, dilated convolution effectively enlarges the receptive field while maintaining low computational overhead. However, this approach compromises the response to high-frequency components [24]. Additionally, in convolution kernel sampling process, deformable convolutions [15, 25, 26] have been employed for various computer vision tasks by introducing asymmetric offsets of $K \times K \times 2$ to increase spatial deviations. However, in object detection tasks where localization sensitivity is critical, maintaining feature consistency becomes essential.

Notably, in autonomous driving images (ADIs), the prevalent traffic environmental noise primarily manifests as low-frequency interference signals in the frequency domain [27, 28, 29], while the texture features of targets are mainly located in the high-frequency signals. Frequency-domain oriented feature extraction could effectively suppress noise and enhance the discernibility of target signals. Traditional signal processing has long relied on frequency-domain analysis as a fundamental tool [30, 31]. Notably, these well-established methods have recently found applications in deep learning, facilitating the learning of non-local features [32, 33, 34, 35, 36] or domain-generalizable representations [37]. An increase in the dilation rate from 1 to D, guided by the scaling principles of Fourier Transform [38, 39], proportionally reduces the frequency response curve and kernel bandwidth by a factor of $\frac{1}{D}$. The diminished bandwidth severely restricts the layer's capacity to handle high-frequency features. Therefore, providing a frequency view for dilated convolution becomes a new approach to improve its capability to capture different frequency information [40].

Back to main

Response to Reviewer 2

Comments

The authors propose a frequency-oriented adaptive detector (FAD) for vehicle-mounted intelligent traffic object detection. The FAD effectively integrates high- and low-frequency information to enhance the texture and semantic features of multi-scale objects in complex traffic scenarios, all while maintaining minimal computational overhead. Experimental results demonstrate that the FAD outperforms state-of-the-art detectors, obtaining the mean average precision (mAP) of 90.9%, 53.7% and 50.2% on KITTI, BDD100K and Cityscapes datasets with a parameter size of only 8.77MB, respectively.

However, the manuscript would be significantly improved by addressing the following issues:

1. The authors emphasize in the abstract that the research motivation is to address perception challenges on in-vehicle platforms. However, the experiments were conducted using a consumer-grade RTX 4070-Ti workstation. My key question is: Can this workstation be considered a compliant automotive-grade hardware platform? If not, the authors should clarify the automotive-grade compliance of the experimental platform, or modify the claims of innovation to accurately reflect the research scope.
2. The authors have conducted validation experiments on the KITTI, BDD100K, and Cityscapes datasets. To further strengthen the robustness of the findings, it is recommended to perform additional validation on larger-scale datasets such as Waymo and nuScenes.
3. While the current manuscript demonstrates algorithmic validation across three standard datasets (KITTI, BDD100K, Cityscapes), we recommend the authors strengthen the application perspective by providing details on platform deployment, real-world performance, and system integration.

Reply: Thank you for your insightful comments on our manuscript. We sincerely appreciate the time and effort you have devoted to providing valuable feedback and constructive suggestions. In response to your comments, we have revised the manuscript accordingly. A point-by-point response is provided below, detailing the modifications made. We hope that our revisions address your concerns and meet your expectations.

Thank you once again for your kind review~

1. The authors emphasize in the abstract that the research motivation is to address perception challenges on in-vehicle platforms. However, the experiments were conducted using a consumer-grade RTX 4070-Ti workstation. My key question is: Can this workstation be considered a compliant automotive-grade hardware platform? If not, the authors should

clarify the automotive-grade compliance of the experimental platform, or modify the claims of innovation to accurately reflect the research scope.

Reply: We sincerely appreciate the reviewer's valuable comment. Your feedback has been extremely helpful in improving the quality and clarity of our manuscript. The experiments in this paper were conducted on an RTX 4070 Ti workstation to ensure sufficient computational resources for model training and evaluation. We fully agree that this platform does not represent an automotive-grade hardware environment.

Revised Content in the Manuscript

4.1 Implementation Specifications

The network is trained under the following configuration. Mosaic data augmentation is applied with a stochastic probability of 0.5. Training is performed using the Stochastic Gradient Descent (SGD) optimizer with a momentum of 0.9, an initial learning rate of 0.01, and cosine annealing to decay the learning rate to 0.0001. Input images are resized to 640×640 pixels and drawn from four datasets, with a batch size of 8. All experiments are conducted in the PyTorch framework on a Windows workstation equipped with an RTX 4070 Ti 12-GB GPU and 32-GB RAM. To further evaluate deployment feasibility, inference is additionally performed on an in-vehicle computing platform RK3588, verifying the deployment suitability and runtime efficiency of the model on an automotive-grade platform.

Page 5

To address this concern, we have added additional experiments and analysis on an in-vehicle computing platform **RK3588** in Section 4.6 to verify the real-time performance and deployment feasibility of our proposed model under automotive-grade hardware conditions. We have also clarified this distinction and experimental setup in the revised manuscript. **A real-time demonstration video has been submitted as supplementary material.** The results demonstrate that FAD maintains real-time inference capability and achieves a favorable trade-off between accuracy and efficiency in real-world edge computing environments.

Since the modifications are extensive, we explain our revisions step by step below for clarity:

Revised Content in the Manuscript

4.6 In-Vehicle Computing Platform Experiments

The in-vehicle computing platform utilized in this study is built upon the **Rockchip RK3588** system-on-chip, which offers high-performance heterogeneous computing and multimedia processing capabilities optimized for real-time visual perception and deep learning inference in autonomous driving scenarios. The schematic layout and hardware specifications of the platform are jointly presented in Fig. 12.

To evaluate the practical performance of the proposed FAD algorithm in dynamic driving conditions, we deployed the inference pipeline primarily on the RK3588's NPU, while the CPU and GPU handled pre-processing and auxiliary computations. The model was initialized using pre-trained weights on the BDD100K dataset to ensure robust generalization across diverse traffic scenarios. Experimental results indicate that FAD achieves an average inference speed of 35.14 FPS at full input resolution, with an average per-frame latency of 28.5 ms, ensuring real-time processing suitable for in-vehicle deployment. FAD maintains high detection accuracy across varying traffic densities and lighting conditions, with negligible impact on mAP. These results highlight the capability of the RK3588's heterogeneous computing architecture to efficiently manage computational load and energy consumption, making it well-suited for the real-time deployment of advanced perception algorithms such as FAD in autonomous driving scenarios.

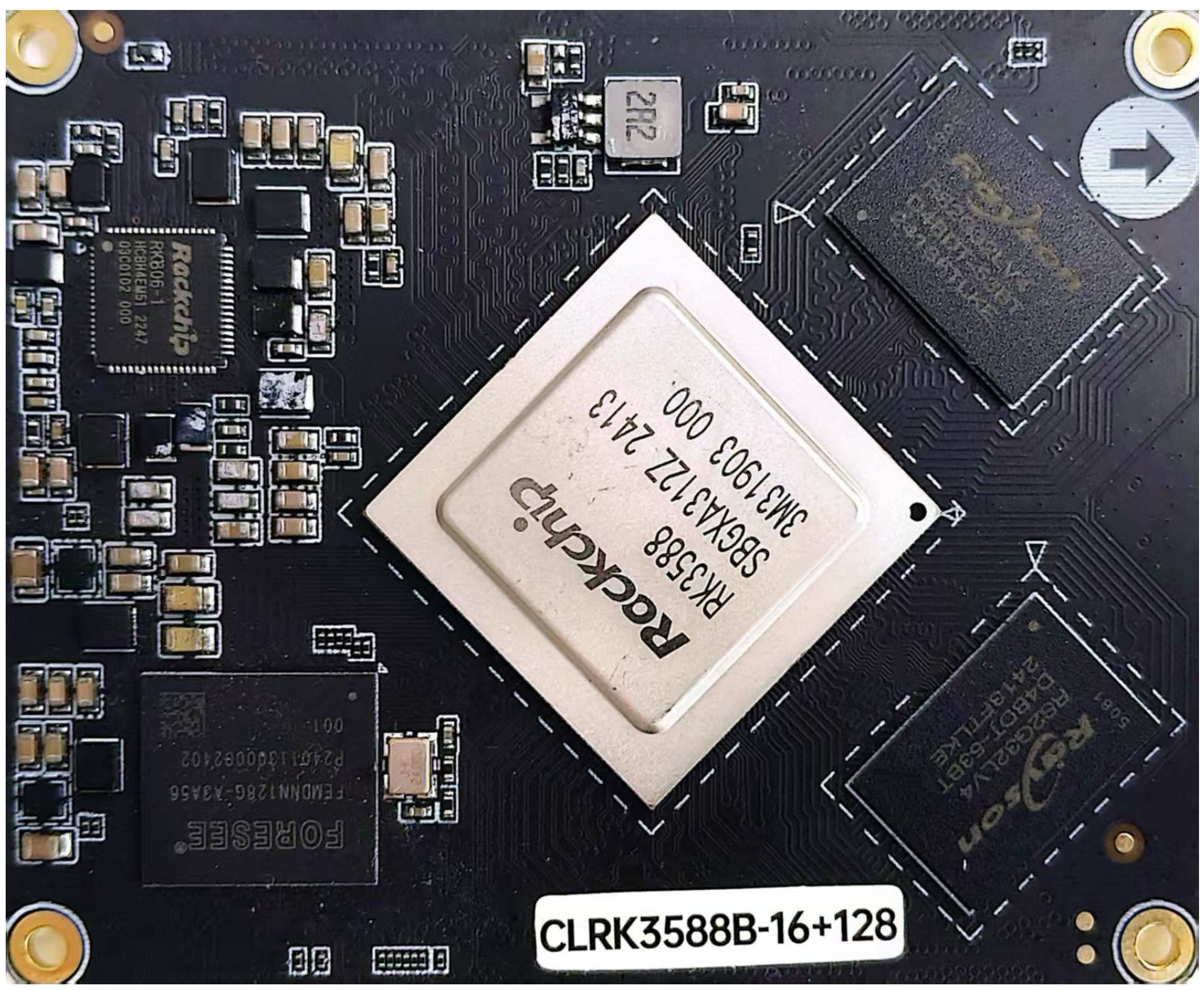

Component	Specification
CPU	4× Cortex-A76 (2.4 GHz) + 4× Cortex-A55 (1.8 GHz)
GPU	Mali-G610 MP4, up to 800 MHz
NPU	6 TOPS (AI inference acceleration)
Memory	8 GB LPDDR4/4X
Storage	32 GB eMMC / UFS 2.1
Power Consumption	Typical 10 W, Max 15 W

Figure 13: Schematic layout and specifications of the RK3588 in-vehicle hardware platform. The module integrates CPU, GPU, NPU, and memory units for efficient on-board deep learning inference.

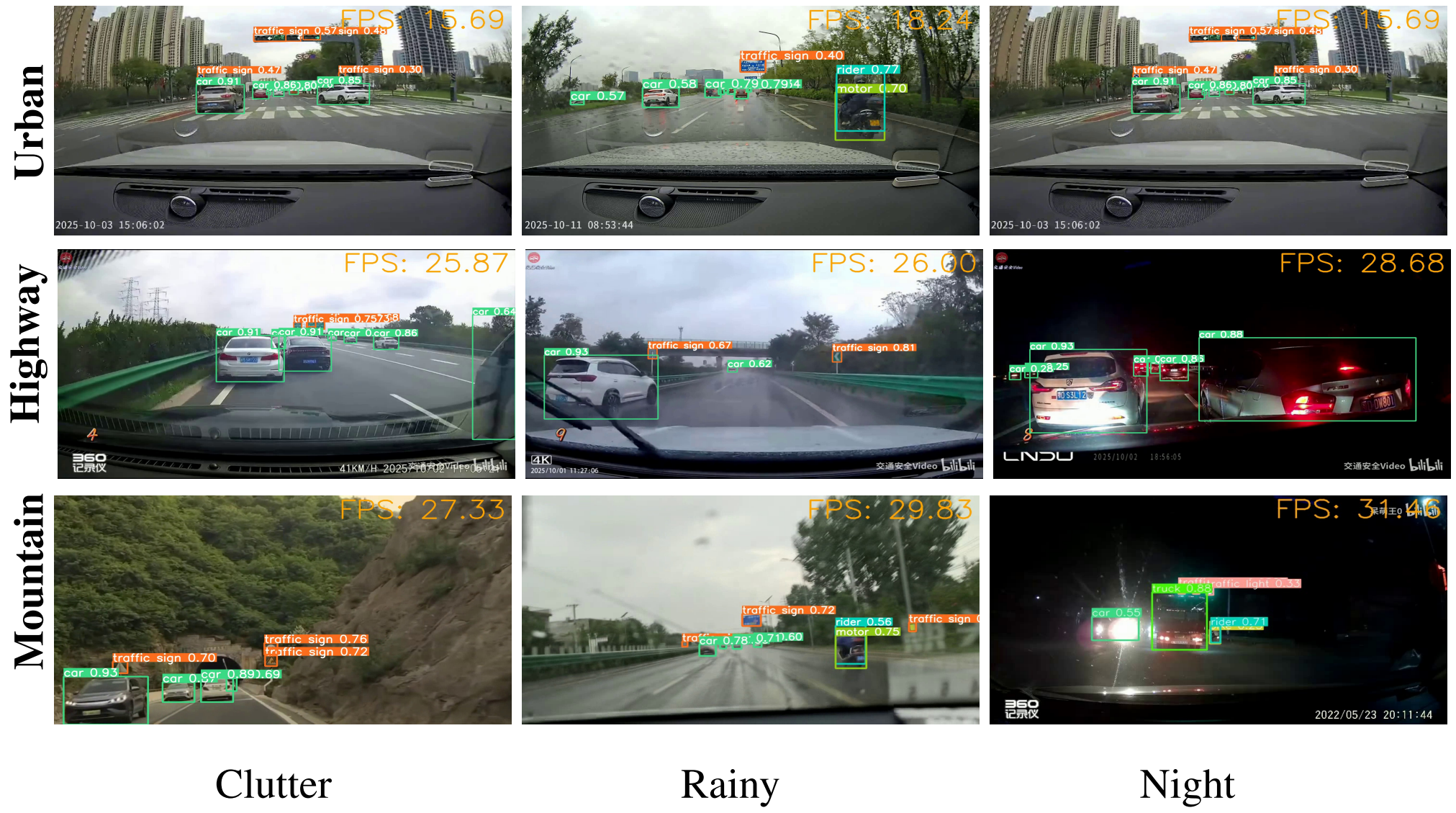

Figure 14: Representative real-time traffic scene detection results of FAD on the RK3588 in-vehicle computing platform, with real-time FPS visualized in the top-right corner.

Page 5

2. The authors have conducted validation experiments on the KITTI, BDD100K, and Cityscapes datasets. To further strengthen the robustness of the findings, it is recommended to perform additional validation on larger-scale datasets such as Waymo and nuScenes.

Reply:

We appreciate the reviewer's insightful suggestion. In response, we have conducted additional experiments on the large-scale Waymo 2D dataset to further validate the robustness and generalization ability of our method. The corresponding results have been included in the revised manuscript in Table 5 and Fig. 11. The consistent performance observed on Waymo 2D dataset demonstrates that our approach generalizes well to large and complex real-world driving scenarios.

Revised Content in the Manuscript

Table 5: Quantitative comparisons of the different methods on Waymo 2D datasets.

Method	mAP@50	mAP@50-95	Vehichle	Person	Cyclist
YOLOv8[41]	51.0	32.7	64.5	56.2	32.4
RT-DETR[42]	52.3	32.6	65.1	56.7	32.6
YOLOv10[43]	51.8	32.9	65.5	57.0	33.1
YOLOv11[44]	51.0	32.5	64.6	56.6	31.8
FAD	**53.1**	**33.0**	**68.0**	**59.7**	31.7

4) Results on Waymo 2D: As shown in Table 5, FAC attains 50.2% mAP on the Cityscapes dataset, exceeding most existing lightweight detectors, including the latest YOLO series and RT-DETR. Such

performance is particularly remarkable given the dataset's emphasis on densely structured urban environments with frequent occlusions and abundant small objects. These results highlight FAC's enhanced ability to perceive fine-grained details and maintain detection stability under complex visual conditions, underscoring its robustness in real-world traffic scenarios.

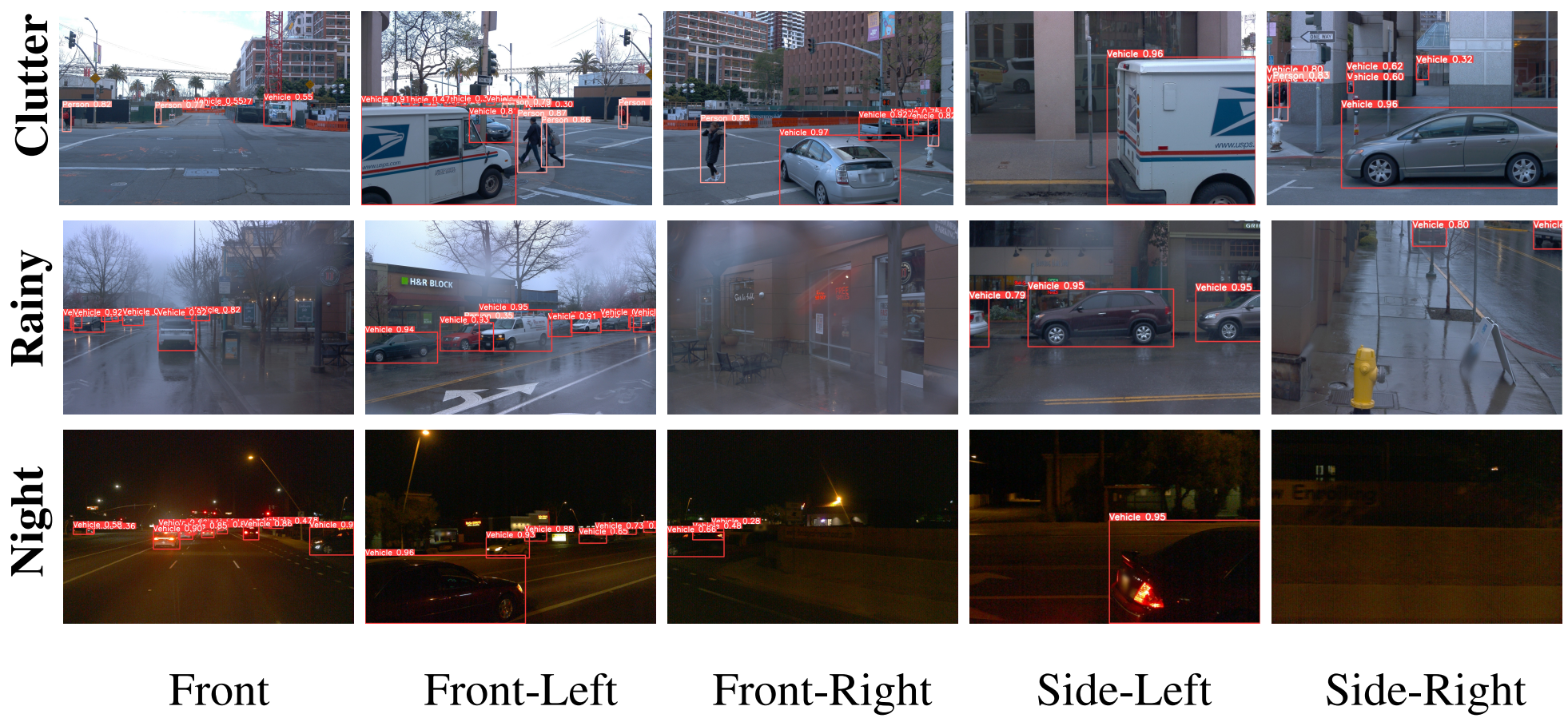

Figure 11: Representative detection results of FAD on the Waymo 2D dataset, illustrating various traffic scenarios captured from 5 on-board cameras, including front, front-left, front-right, side-left, and side-right views.

For Waymo 2D, which features real-world autonomous driving scenarios captured simultaneously from five synchronized on-board cameras, FAD demonstrates robust multi-scale object detection across diverse traffic conditions. As shown in Fig. 11, FAD accurately detects occluded vehicles, distant small objects, and objects under varying weather and lighting conditions. These results highlight FAD's ability to handle complex real-world traffic scenes with multi-camera views, reinforcing its generalization and effectiveness across large-scale autonomous driving datasets.

To ensure fairness and better reflect current research progress, we have expanded our comparisons to include several recent state-of-the-art detectors, including YOLOv8, YOLOv10, YOLOv11, and RT-DETR. All models were trained and evaluated under consistent lightweight settings to maintain comparability. The updated results in Table 4 show that our proposed FAD consistently outperforms both classical and modern baselines, confirming its robustness and generalization capability across diverse detection frameworks. Furthermore, we have validated the effectiveness of FAD on the large-scale autonomous driving dataset Waymo, demonstrating its strong adaptability and reliability in complex real-world traffic scenarios.

Revised Content in the Manuscript

Table 3: Detection Performance Comparison with Related Works on the KITTI Validation Set Across Classes and Difficulties.

Method	mAP	Car			Pedestrian			Cyclist			FPS
		Easy	Mod.	Hard	Easy	Mod.	Hard	Easy	Mod.	Hard	
5-Ensemble[45]	73.70	97.84	89.75	80.87	77.08	67.66	59.17	79.45	57.30	54.17	5.10
CertainNet[46]	73.00	93.81	89.36	82.11	76.33	66.13	58.54	78.02	57.49	55.20	16.46
Centernet[47]	71.49	90.30	89.15	82.17	76.53	67.53	59.37	73.48	52.63	50.25	20.21
SSD[48]	60.79	87.34	87.74	77.27	50.38	48.41	43.46	48.25	52.31	52.13	33.17
ASSD[49]	75.28	89.28	89.95	82.11	69.07	62.49	60.18	75.23	76.16	72.83	33.04
SA-YOLO[50]	83.82	91.71	87.16	80.97	86.49	80.63	76.91	87.28	83.44	79.83	32.68
YOLOv8[41]	89.31	98.32	98.50	90.54	90.63	87.65	80.03	89.26	87.01	79.39	69.04
YOLOv10[43]	88.80	98.58	98.60	90.53	90.74	86.69	86.91	89.28	87.04	79.21	66.33
YOLOv11[44]	88.40	98.46	98.37	90.44	90.45	85.00	87.96	87.98	85.76	78.21	85.49
**FAD**	**90.90**	98.55	**98.91**	**90.63**	89.85	**88.16**	**88.13**	**90.84**	**90.33**	**89.71**	57.60

Table 4: Quantitative comparisons of the different methods on KITTI, BDD100K and Cityscapes datasets. Our proposed method achieves the best metrical scores across the three datasets.

Method	KITTI			Method	BDD100K			Method	Cityscapes		
	mAP	Params	GFLOPS		mAP	FPS	Params		mAP	Params	GFLOPS
SA-YOLO[50]	81.8	–	–	CertainNet[46]	40.9	–	–	MDNet[61]	37.0	15.11	**12.3**
YOLOv3[7]	82.2	234.94	65.3	SA-YOLO[50]	17.3	48.5	–	DAYOLO[64]	49.3	9.35	19.7
MobileSSD[51]	71.8	33.86	12.5	CenterNet[47]	44.5	46.0	93.50	PDEM[65]	43.3	–	26.7
EdgeYOLO[52]	82.1	24.48	**9.9**	YOLOv7[10]	44.3	71.0	42.00	UMS^2-ODNet[66]	48.3	34.9	85.9
BHF-MTM[53]	88.6	35.80	–	PASFLN[58]	46.3	40.0	117.00	Faster R-CNN[67]	40.8	41.5	180.5
FD-Head[54]	90.5	–	23.2	SDA-OD[59]	42.8	33.0	–	YOLOv7[10]	33.5	36.9	55.5
YOLOX[11]	89.0	9.00	26.7	OFFR-YOLO[60]	45.3	-	-	PWNet[68]	49.1	–	–
YOLOv8[55]	89.3	11.20	28.7	MDNet[61]	45.0	46.0	14.80	CPSS-FAT[69]	39.4	48.4	181.1
SHW[56]	86.8	8.30	22.9	SpikeTOD[62]	43.7	–	–	AugFCOS[70]	42.3	–	–
PIAENet[57]	83.4	17.90	3.2	CSIM[63]	50.8	–	–	IRG[71]	47.7	–	–
YOLOv8[41]	89.3	11.40	29.7	YOLOv8[41]	51.0	69.8	29.7	YOLOv8[41]	47.9	11.40	29.7
YOLOv10[43]	88.8	9.17	24.8	YOLOv10[43]	51.8	67.3	24.8	YOLOv10[43]	47.3	9.17	24.8
YOLOv11[44]	88.4	9.40	21.5	YOLOv11[44]	51.5	80.7	21.5	YOLOv11[44]	47.8	9.40	21.5
RT-DETR[42]	88.5	42.70	136.1	RT-DETR[42]	51.6	45.1	136.1	RT-DETR[42]	48.5	42.70	136.1
**Ours**	**90.9**	**8.77**	39.7	**Ours**	**53.7**	55.6	**8.77**	**Ours**	**50.2**	**8.77**	39.7

4.4 Comparison Experiments and Quantitative Analysis

1) Results on KITTI: We compare our method with state-of-the-art approaches to the KITTI validation criteria, and detailed results are shown in Table 3. FAD achieves the highest mAP of 90.90%, outperforming all competitors. This highlights the effectiveness of our proposed method. Additionally, our approach excels in 2 specific categories: car and cyclist. Notably, FAD achieves an FPS of 57.60, demonstrating the balance between performance and computational resources achieved by our approach.

As shown in Table 4, our method surpasses the mainstream YOLO series and RT-DETR in mAP. Notably, it achieves the highest mAP while maintaining exceptional efficiency, with only 8.77M parameters—making it the most lightweight model among all compared approaches.

2) Results on BDD100K: We conducted a comprehensive evaluation of FAD on the BDD100K dataset, benchmarking it against the latest advancements in the field. FAD notably outperforms its

counterparts, achieving a remarkable mAP of over 53.7% (as displayed in Table 4). Our method showcases exceptional generalization capabilities, demonstrating outstanding performance even on larger traffic datasets. Notably, with an input image size of 640×640, FAD surpasses all existing detectors, boasting an impressive FPS of 55.6 while maintaining the smallest model size among its peers.

Moreover, our achieved mAP of 53.7% represents a substantial 1.9% improvement over the runner-up, YOLOv11. Additionally, the visualization results presented in Fig. 11 vividly demonstrates FAD's precision in detecting objects across diverse scales and aspect ratios, effectively handling complex backgrounds, occlusions, low-quality objects, dense scenes, nighttime conditions, and small objects.

3) Results on Cityscapes: As shown in Table 4, FAC attains 50.2% mAP on the Cityscapes dataset, exceeding most existing lightweight detectors, including the latest YOLO series and RT-DETR. Such performance is particularly remarkable given the dataset's emphasis on densely structured urban environments with frequent occlusions and abundant small objects. These results highlight FAC's enhanced ability to perceive fine-grained details and maintain detection stability under complex visual conditions, underscoring its robustness in real-world traffic scenarios.

3. While the current manuscript demonstrates algorithmic validation across three standard datasets (KITTI, BDD100K, Cityscapes), we recommend the authors strengthen the application perspective by: platform deployment details, real-world performance, and system integration.

Reply: We thank the reviewer for this valuable suggestion. In the revised manuscript, we have substantially reinforced the application perspective by incorporating comprehensive deployment details and real-world evaluation. Specifically, we describe the implementation of the proposed FAD algorithm on the Rockchip RK3588 in-vehicle computing platform, highlighting the heterogeneous CPU–GPU–NPU collaboration and runtime optimization strategies. Furthermore, we include real-time detection results across representative driving scenarios, reporting average per-frame latency of 28.5 ms, and 35.14 FPS to substantiate the system's real-time capability. These additions collectively demonstrate the algorithm's seamless integration into embedded platforms and its robustness under practical deployment conditions.

Back to main

Response to Reviewer 3

Comments

The paper proposes a frequency-oriented adaptive detector (FAD) for real-time traffic object detection. The key contributions are (1) Frequency Dynamic Convolution (FDC), which decomposes convolution kernels into high- and low-frequency components and adaptively adjusts dilation rates; (2) Adaptive Frequency-oriented Fusion (AFF), which applies low-pass/high-pass adaptive filters, offset correction, and cross-scale contribution allocation to better combine features at different levels. The authors evaluate the method on KITTI, BDD100K, and Cityscapes, claiming superior performance over existing detectors with a lightweight computational cost.

I found this paper has the following strengths: firstly, this work focuses on a practically relevant problem (lightweight object detection in autonomous driving under cluttered scenes). Secondly, both the FDC and AFF modules are described in detail, with equations and visualizations. Thirdly, experiments are conducted on three widely used benchmarks with ablation studies.

However, the proposed ideas largely combine existing concepts (frequency-domain analysis, dilated convolution adaptation, feature pyramid fusion). FDC essentially resembles prior works on deformable/dynamic convolutions or spectral-aware convolution (e.g., frequency-aware CNNs, adaptive filtering) with minimal new insights. AFF is essentially a re-implementation of feature alignment + multi-scale fusion strategies (similar to CARAFE, FaPN, AlignSeg, etc.), only rephrased with frequency terminology. So, the “frequency” perspective is more a reinterpretation rather than a fundamentally new method. The claimed novelty is incremental and not sufficient for the NC.

In addition, baselines are somewhat outdated: comparisons focus on YOLOv3, YOLOX, YOLOv8, etc., but do not include more recent lightweight detectors or transformer-based architectures that dominate current literature. And most comparisons are from IEEE TITS, not from top-tier journals like NC. And reported improvements (1–3% mAP) are marginal and within the range of hyperparameter tuning or stronger data augmentation. No statistical significance analysis is provided. And this work only presents simulation results, did not conduct any real-world experiments. Therefore, the paper presents an application-driven detector design but lacks substantial novelty, rigorous justification, and convincing experimental validation. The main contributions are incremental improvements over existing adaptive convolution and feature fusion strategies, with limited theoretical depth and modest empirical gains. Writing is verbose, and the comparisons do not cover the true state of the art.

Reply: Thank you for your insightful comments on our manuscript. We sincerely appreciate the time and effort you have devoted to providing valuable feedback and constructive suggestions. In response to your comments, we have revised the manuscript accordingly. A point-by-point

response is provided below, detailing the modifications made. We hope that our revisions address your concerns and meet your expectations.

Thank you once again for your kind review~

1. The proposed ideas largely combine existing concepts (frequency-domain analysis, dilated convolution adaptation, feature pyramid fusion). FDC essentially resembles prior works on deformable/dynamic convolutions or spectral-aware convolution (e.g., frequency-aware CNNs, adaptive filtering) with minimal new insights. AFF is essentially a re-implementation of feature alignment + multi-scale fusion strategies (similar to CARAFE, FaPN, AlignSeg, etc.), only rephrased with frequency terminology. So, the "frequency" perspective is more a reinterpretation rather than a fundamentally new method. The claimed novelty is incremental and not sufficient for the NC. In addition, baselines are somewhat outdated: comparisons focus on YOLOv3, YOLOX, YOLOv8, etc., but do not include more recent lightweight detectors or transformer-based architectures that dominate current literature. And most comparisons are from IEEE TITS, not from top-tier journals like NC. And reported improvements (1–3% mAP) are marginal and within the range of hyperparameter tuning or stronger data augmentation. No statistical significance analysis is provided. And this work only presents simulation results, did not conduct any real-world experiments. Therefore, the paper presents an application-driven detector design but lacks substantial novelty, rigorous justification, and convincing experimental validation. The main contributions are incremental improvements over existing adaptive convolution and feature fusion strategies, with limited theoretical depth and modest empirical gains. Writing is verbose, and the comparisons do not cover the true state of the art.

Reply: Thank you for your insightful comments on our manuscript. We sincerely appreciate the time and effort you have devoted to providing valuable feedback and constructive suggestions. In response to your comments, we have revised the manuscript accordingly. A point-by-point response is provided below, detailing the modifications made. We hope that our revisions address your concerns and meet your expectations.

For your convenience, the corresponding revised content is attached in green box below, with page numbers from the manuscript shown in the lower-right corner of green box. For clarity, all modifications are also marked in purple in the revised manuscript.

1. The proposed ideas largely combine existing concepts (frequency-domain analysis, dilated convolution adaptation, feature pyramid fusion). FDC essentially resembles prior works on deformable/dynamic convolutions or spectral-aware convolution (e.g., frequency-aware CNNs, adaptive filtering) with minimal new insights. AFF is essentially a re-implementation of feature alignment + multi-scale fusion strategies (similar to CARAFE, FaPN, AlignSeg,

etc.), only rephrased with frequency terminology. So, the "frequency" perspective is more a reinterpretation rather than a fundamentally new method. The claimed novelty is incremental and not sufficient for the NC.

Reply:

We sincerely thank the reviewer for their insightful feedback. We acknowledge that some elements of our method, such as frequency-domain analysis, dilated convolution adaptation, and feature pyramid fusion, may seem conceptually aligned with prior works in deformable convolutions, spectral-aware convolutions, and multi-scale fusion techniques. However, the core novelty of our approach lies in the reinterpretation and integration of these concepts through a frequency-based perspective, which introduces a systematic and novel method for addressing scale variations and feature alignment.

While the frequency-based approach draws inspiration from the foundational principles of spectral analysis, it extends beyond the mere rephrasing of existing methods. By leveraging frequency-domain processing, it offers a new framework for optimizing feature extraction and fusion across multiple scales and resolutions. This perspective not only enhances feature alignment but also improves processing efficiency—advantages that were not fully explored in prior models. Although the work might appear incremental from one perspective, it represents a significant advancement in the application of existing techniques. This reinterpretation of well-established methods presents a new approach for improving real-time performance and robustness in complex visual tasks. To clarify these contributions, we have updated the manuscript and provided additional experimental results to validate the effectiveness of the proposed approach.

Furthermore, the novelty of our method lies in the integration of frequency-domain filtering mechanisms with adaptive feature fusion. Specifically, AFF consists of two key stages: the frequency-guided adjacent scale fusion and the Cross-scale Contribution Allocation (CCA) strategy for non-adjacent scale fusion.

In the adjacent scale fusion, LAF smooths coarse high-level features and reduces pixel value disparities, addressing low intra-category similarity. The offset generation allows for precise boundary refinement by resampling high-similarity regions, overcoming the limitations of standard multi-scale fusion. HAF extracts high-frequency boundary details that are typically lost during standard downsampling procedures, thereby improving boundary definition beyond the Nyquist limit.

In the non-adjacent scale fusion, to assign varying spatial weights to features from different levels during the multi-level feature fusion process, we propose the Cross-scale Contribution Allocation (CCA) module, which enhances the importance of key levels while mitigating the impact of conflicting information from different objects. This fusion approach, depicted in Fig. 6, effi-

ciently merges features from four scales. Moreover, to accommodate the varying number of fused features at different stages of the AFF, we introduce a stage-specific number of adaptive spatial fusion modules at each stage. This process allows CCA to progressively merge high-level and low-level features from non-adjacent scales, bridging the semantic gaps that often cause feature misalignment.

The incremental novelty of our method is not only in the components themselves but also in how these components are coordinated to tackle specific challenges related to intra-category consistency and object boundary precision—crucial elements for real-time detection in dynamic, high-traffic environments. We believe that this unique perspective on frequency-domain processing provides valuable insights into object detection tasks, contributing to the development of more robust, efficient detection systems, particularly for resource-constrained environments such as autonomous driving.

We sincerely hope that these revisions address your concerns. For your convenience, the corresponding revised content is highlighted in a green box below, with page numbers from the manuscript shown in the lower-right corner of the green box. Additionally, all modifications are marked in purple in the revised manuscript.

Revised Content in the Manuscript

1 Introduction

Vision-based traffic scene perception has emerged as a key research frontier in intelligent transportation systems, enabling the detection, recognition, and tracking of diverse road participants. Accurate object detection forms the foundation of reliable scene understanding, serving as a critical component for downstream tasks such as autonomous navigation and traffic management. However, vehicle-mounted traffic object detection (TOD) remains a challenging task due to several inherent difficulties, including scale variation induced by perspective changes, frequent occlusions, diverse lighting and weather conditions, and the limited performance of lightweight detection networks when handling low-quality or small-scale targets. Representative examples of these complexities are illustrated in Fig. 1, drawn from the KITTI [1], BDD100K [2], and Cityscapes [3] datasets.

Deep learning–based object detection algorithms can be broadly categorized into two main paradigms: two-stage and one-stage detectors. Two-stage approaches, typified by Faster R-CNN [4], first generate region proposals and then perform classification and localization, offering high accuracy but at the expense of computational efficiency. In contrast, one-stage methods such as SSD [5], YOLO [6, 7, 8, 9, 10, 11], and RetinaNet [12] treat detection as a direct regression problem within a single network, achieving a more favorable trade-off between speed and precision. Nonetheless, deploying these models on embedded vehicle platforms remains challenging due to constraints on GPU capacity and memory resources, which limit the practicality of computation-heavy detectors in real-world

applications.

In pursuit of a balance between performance and computational efficiency on vehicle-mounted platforms, a variety of model compression strategies and enhanced network architectures have been developed to improve detection efficiency. In terms of feature extraction, recent studies have highlighted the crucial role of a large receptive field in scene understanding tasks [13, 14], yet expanding it inevitably increases computational complexity. To mitigate this trade-off, several works have explored content-adaptive mechanisms [15, 16, 17, 18, 19, 20], which dynamically adjust the convolutional process to input variations. Vision transformers [14, 21, 22, 23] further extend this paradigm by employing self-attention to model long-range dependencies, but their high computational burden limits their deployment on embedded systems. Alternatively, dilated convolution enlarges the receptive field by inserting intervals between filter elements at a predefined dilation rate D, achieving broader contextual perception with low computational overhead, though at the expense of responsiveness to high-frequency components [24]. Meanwhile, deformable convolutions [15, 25, 26] introduce asymmetric spatial offsets of $K \times K \times 2$ to adaptively capture geometric transformations across diverse visual tasks. Yet, in object detection—where precise localization is crucial—maintaining feature consistency remains a significant challenge.

Notably, in autonomous driving scenes, environmental noise predominantly appears as low-frequency interference in the frequency domain [27, 28, 29], whereas the structural and textural details of traffic targets are mainly concentrated in high-frequency components. Leveraging this spectral distinction, frequency-domain feature extraction can effectively suppress background noise while enhancing the representation of salient targets. Frequency-domain analysis has long been a cornerstone of signal processing [30, 31] and has recently been incorporated into deep learning frameworks to model non-local dependencies [32, 33, 34, 35, 36] and improve domain generalization [37]. According to the scaling law of the Fourier Transform [38, 39], increasing the dilation rate from 1 to D proportionally reduces the frequency response bandwidth by $\frac{1}{D}$, thereby diminishing the model's sensitivity to high-frequency details. This observation motivates the integration of frequency-domain principles into convolutional network design to enhance multi-frequency feature representation [40].

Building upon these insights, we propose **Frequency Dynamic Convolution (FDC)**, a novel convolutional operator designed to extract object features from frequency-domain noise in complex traffic scenes, eliminate spatial deviations, and enhance feature consistency for position-sensitive regression. FDC refines conventional dilated convolution through spectral analysis, introducing a unified single-value dilation rate that reduces redundant coordinate sampling inherent in standard convolutions. Its frequency-guided dilation strategy alleviates the heuristic and non-interpretable nature of empirically defined dilation parameters. By dynamically adjusting the dilation rate in a spatially adaptive manner, FDC optimizes kernel bandwidth utilization and learns frequency-balancing functions that promote broader receptive fields, thereby achieving a principled equilibrium between effective bandwidth and receptive field coverage.

Moreover, autonomous driving scenes pose substantial challenges, including background noise, illumination variation, motion blur, and multi-scale traffic objects induced by perspective effects, thereby necessitating the extraction of features with strong category semantics and precise spatial boundaries. To address these challenges, feature fusion [72] has been widely adopted in various architectures [73, 74, 75, 76] to integrate high-level semantic features with low-level high-resolution details. In general, high-level features provide rich categorical context, whereas low-level features preserve fine-grained spatial details. However, upsampling coarse features via nearest-neighbor or bilinear interpolation and merging them with high-resolution features often leads to *intra-category inconsistency* and *boundary displacement* [40]. The former arises from variations within the same object, such as differences in texture and brightness between a vehicle's wheel and window [77], while the latter is caused by excessive smoothing introduced during interpolation [78, 79]. Conventional fusion methods [75] are unable to effectively address these issues and may even exacerbate them by diffusing a single feature across multiple pixels. To quantify these phenomena, feature similarity analysis is employed, as illustrated in Fig. 2. Intra-category inconsistency is evaluated through *intra-category similarity*, which measures the similarity between each feature vector and its corresponding category center [80]. Likewise, *inter-category similarity* is computed to assess the similarity margin between different classes. Boundary displacement is characterized by low intra-category similarity and small similarity margins in boundary regions. Since classification scores depend on the similarity between category-aware weights and feature representations [81], features with reduced intra-category similarity and margin ultimately yield lower classification confidence, leading to potential misclassifications.

Therefore, rapid variations in feature values within traffic objects, corresponding to disturbed high-frequency components, lead to low intra-category similarity and feature inconsistency [82]. Additionally, blurred boundaries lack precise high-frequency information, resulting in boundary displacement. Frequency-domain processing is essential for maintaining intra-category consistency and precise object boundaries, as it enables effective preservation and adaptive utilization of high-frequency features. To leverage frequency-domain information for improved feature fusion, we propose an adaptive frequency-oriented fusion (AFF) framework. AFF consists of four key components: Low-pass Adaptive Filter (LAF) generator, offset generator, High-pass Adaptive Filter (HAF) generator, and Cross-scale Contribution Allocator (CCA).

Specifically, the LAF applies a low-pass filter to smooth and upsample coarse high-level features, reducing disparities between pixel values and mitigating feature inconsistency. LAF alone is insufficient to correct large-area inconsistencies or refine boundaries. To address this limitation, we introduce an offset generator. The offset generator first calculates local similarity and then predicts offsets toward regions of high similarity for resampling, effectively replacing features with low intra-category similarity. It rectifies inconsistent features across both large regions and narrow boundary areas. Restoring fine boundary details lost during downsampling of low-level features into high-level representations remains challenging. According to the Nyquist-Shannon sampling theorem [83, 84], frequencies exceeding the

Nyquist threshold, defined as half the sampling rate, are irretrievably lost. To address this limitation, we introduce the HAF generator, which applies spatially adaptive high-pass filters to low-level features, extracting intricate boundary details and enhancing high-frequency energy beyond the Nyquist limit. During bottom-up feature integration, the CCA progressively fuses high-level features with low-level features from non-adjacent scales, reducing the semantic gap between disparate scales. Through the coordinated operation of these four components, AFF reconstructs fused features that preserve consistent category information and well-defined object boundaries in complex traffic scenarios.

Our proposed frequency-oriented adaptive detector (FAD) integrates Frequency Dynamic Convolution (FDC) for frequency-guided feature extraction with Adaptive Frequency-oriented Fusion (AFF) for frequency-guided adaptive feature integration. By explicitly capturing high- and low-frequency components, FAD strengthens both the textural and semantic features of objects across scales. By explicitly leveraging high- and low-frequency components, FAD enhances multi-scale object detection in complex traffic environments while preserving inference efficiency. We delineate our principal contributions as follows.

3 Methodology

2. In addition, baselines are somewhat outdated: comparisons focus on YOLOv3, YOLOX, YOLOv8, etc., but do not include more recent lightweight detectors or transformer-based architectures that dominate current literature. And most comparisons are from IEEE TITS, not from top-tier journals like NC. And reported improvements (1–3% mAP) are marginal and within the range of hyperparameter tuning or stronger data augmentation. No statistical significance analysis is provided. And this work only presents simulation results, did not conduct any real-world experiments.

Reply:

We appreciate the reviewer's thoughtful comment regarding the fairness and contemporaneity of baseline comparisons. Our initial selection of detectors (e.g., SSD, YOLOv3) was motivated by their frequent adoption in recent studies as standard benchmarks for evaluating lightweight object detection performance.

To ensure fairness and better reflect current research progress, we have expanded our comparisons to include several recent state-of-the-art detectors, including YOLOv8, YOLOv10, YOLOv11, and RT-DETR. All models were trained and evaluated under consistent lightweight settings to maintain comparability. The updated results in Table 4 show that our proposed FAD consistently outperforms both classical and modern baselines, confirming its robustness and generalization capability across diverse detection frameworks. Furthermore, we have validated the effectiveness of FAD on the large-scale autonomous driving dataset Waymo, demonstrating its strong adaptability

and reliability in complex real-world traffic scenarios.

Revised Content in the Manuscript

Table 3: Detection Performance Comparison with Related Works on the KITTI Validation Set Across Classes and Difficulties.

Method	mAP	Car			Pedestrian			Cyclist			FPS
		Easy	Mod.	Hard	Easy	Mod.	Hard	Easy	Mod.	Hard	
5-Ensemble[45]	73.70	97.84	89.75	80.87	77.08	67.66	59.17	79.45	57.30	54.17	5.10
CertainNet[46]	73.00	93.81	89.36	82.11	76.33	66.13	58.54	78.02	57.49	55.20	16.46
Centernet[47]	71.49	90.30	89.15	82.17	76.53	67.53	59.37	73.48	52.63	50.25	20.21
SSD[48]	60.79	87.34	87.74	77.27	50.38	48.41	43.46	48.25	52.31	52.13	33.17
ASSD[49]	75.28	89.28	89.95	82.11	69.07	62.49	60.18	75.23	76.16	72.83	33.04
SA-YOLO[50]	83.82	91.71	87.16	80.97	86.49	80.63	76.91	87.28	83.44	79.83	32.68
YOLOv8[41]	89.31	98.32	98.50	90.54	90.63	87.65	80.03	89.26	87.01	79.39	69.04
YOLOv10[43]	88.80	98.58	98.60	90.53	90.74	86.69	86.91	89.28	87.04	79.21	66.33
YOLOv11[44]	88.40	98.46	98.37	90.44	90.45	85.00	87.96	87.98	85.76	78.21	85.49
**FAD**	**90.90**	98.55	**98.91**	**90.63**	89.85	**88.16**	**88.13**	**90.84**	**90.33**	**89.71**	57.60

Table 4: Quantitative comparisons of the different methods on KITTI, BDD100K and Cityscapes datasets. Our proposed method achieves the best metrical scores across the three datasets.

Method	KITTI			Method	BDD100K			Method	Cityscapes		
	mAP	Params	GFLOPS		mAP	FPS	Params		mAP	Params	GFLOPS
SA-YOLO[50]	81.8	–	–	CertainNet[46]	40.9	–	–	MDNet[61]	37.0	15.11	**12.3**
YOLOv3[7]	82.2	234.94	65.3	SA-YOLO[50]	17.3	48.5	–	DAYOLO[64]	49.3	9.35	19.7
MobileSSD[51]	71.8	33.86	12.5	CenterNet[47]	44.5	46.0	93.50	PDEM[65]	43.3	–	26.7
EdgeYOLO[52]	82.1	24.48	**9.9**	YOLOv7[10]	44.3	71.0	42.00	UMS^2-ODNet[66]	48.3	34.9	85.9
BHF-MTM[53]	88.6	35.80	–	PASFLN[58]	46.3	40.0	117.00	Faster R-CNN[67]	40.8	41.5	180.5
FD-Head[54]	90.5	–	23.2	SDA-OD[59]	42.8	33.0	–	YOLOv7[10]	33.5	36.9	55.5
YOLOX[11]	89.0	9.00	26.7	OFFR-YOLO[60]	45.3	-	-	PWNet[68]	49.1	–	–
YOLOv8[55]	89.3	11.20	28.7	MDNet[61]	45.0	46.0	14.80	CPSS-FAT[69]	39.4	48.4	181.1
SHW[56]	86.8	8.30	22.9	SpikeTOD[62]	43.7	–	–	AugFCOS[70]	42.3	–	–
PIAENet[57]	83.4	17.90	3.2	CSIM[63]	50.8	–	–	IRG[71]	47.7	–	–
YOLOv8[41]	89.3	11.40	29.7	YOLOv8[41]	51.0	69.8	29.7	YOLOv8[41]	47.9	11.40	29.7
YOLOv10[43]	88.8	9.17	24.8	YOLOv10[43]	51.8	67.3	24.8	YOLOv10[43]	47.3	9.17	24.8
YOLOv11[44]	88.4	9.40	21.5	YOLOv11[44]	51.5	80.7	21.5	YOLOv11[44]	47.8	9.40	21.5
RT-DETR[42]	88.5	42.70	136.1	RT-DETR[42]	51.6	45.1	136.1	RT-DETR[42]	48.5	42.70	136.1
**Ours**	**90.9**	**8.77**	39.7	**Ours**	**53.7**	55.6	**8.77**	**Ours**	**50.2**	**8.77**	39.7

4.4 Comparison Experiments and Quantitative Analysis

1) Results on KITTI: We compare our method with state-of-the-art approaches to the KITTI validation criteria, and detailed results are shown in Table 3. FAD achieves the highest mAP of 90.90%, outperforming all competitors. This highlights the effectiveness of our proposed method. Additionally, our approach excels in 2 specific categories: car and cyclist. Notably, FAD achieves an FPS of 57.60, demonstrating the balance between performance and computational resources achieved by our approach.

As shown in Table 4, our method surpasses the mainstream YOLO series and RT-DETR in mAP. Notably, it achieves the highest mAP while maintaining exceptional efficiency, with only 8.77M parameters—making it the most lightweight model among all compared approaches.

2) Results on BDD100K: We conducted a comprehensive evaluation of FAD on the BDD100K dataset, benchmarking it against the latest advancements in the field. FAD notably outperforms its counterparts, achieving a remarkable mAP of over 53.7% (as displayed in Table 4). Our method showcases exceptional generalization capabilities, demonstrating outstanding performance even on larger traffic datasets. Notably, with an input image size of 640×640, FAD surpasses all existing detectors, boasting an impressive FPS of 55.6 while maintaining the smallest model size among its peers.

Moreover, our achieved mAP of 53.7% represents a substantial 1.9% improvement over the runner-up, YOLOv11. Additionally, the visualization results presented in Fig. 11 vividly demonstrates FAD's precision in detecting objects across diverse scales and aspect ratios, effectively handling complex backgrounds, occlusions, low-quality objects, dense scenes, nighttime conditions, and small objects.

3) Results on Cityscapes: As shown in Table 4, FAC attains 50.2% mAP on the Cityscapes dataset, exceeding most existing lightweight detectors, including the latest YOLO series and RT-DETR. Such performance is particularly remarkable given the dataset's emphasis on densely structured urban environments with frequent occlusions and abundant small objects. These results highlight FAC's enhanced ability to perceive fine-grained details and maintain detection stability under complex visual conditions, underscoring its robustness in real-world traffic scenarios.

All experiments were conducted under identical hyperparameter settings and training protocols, without any additional hyperparameter tuning or stronger data augmentation, to ensure a fair and unbiased comparison across all methods. Initial training and evaluation were performed on an RTX 4070 Ti workstation, providing sufficient computational resources for stable optimization and reproducibility. We acknowledge that this workstation does not fully reflect the computational constraints of automotive-grade hardware. To address this limitation, FAD was further deployed and tested on an embedded in-vehicle computing platform (RK3588), as detailed in Section **??**. The experimental results demonstrate that FAD achieves real-time inference while maintaining a favorable balance between detection accuracy and computational efficiency under realistic embedded hardware conditions. A real-time demonstration video is also provided as supplementary material, illustrating the model's practical performance in dynamic, real-world traffic environments.

Revised Content in the Manuscript

4.6 In-Vehicle Computing Platform Experiments

The in-vehicle computing platform utilized in this study is built upon the **Rockchip RK3588** system-on-chip, which offers high-performance heterogeneous computing and multimedia processing capabilities optimized for real-time visual perception and deep learning inference in autonomous driving

scenarios. The schematic layout and hardware specifications of the platform are jointly presented in Fig. 12.

To evaluate the practical performance of the proposed FAD algorithm in dynamic driving conditions, we deployed the inference pipeline primarily on the RK3588's NPU, while the CPU and GPU handled pre-processing and auxiliary computations. The model was configured with an input resolution of 640×640 and converted into the RKCNN format to fully exploit the NPU's parallel acceleration capability. Leveraging hardware-level NPU inference substantially reduced computational latency and ensured stable real-time performance. FAD was initialized using pre-trained weights on the BDD100K dataset to ensure robust generalization and performance in real-world traffic scenarios. Experimental results indicate that FAD achieves an average inference speed of 35.14 FPS at full input resolution, with an average per-frame latency of 28.5 ms, ensuring real-time processing suitable for in-vehicle deployment. FAD maintains high detection accuracy across varying traffic densities and lighting conditions, with negligible impact on mAP. These results highlight the capability of the RK3588's heterogeneous computing architecture to efficiently manage computational load and energy consumption, making it well-suited for the real-time deployment of advanced perception algorithms such as FAD in autonomous driving scenarios.

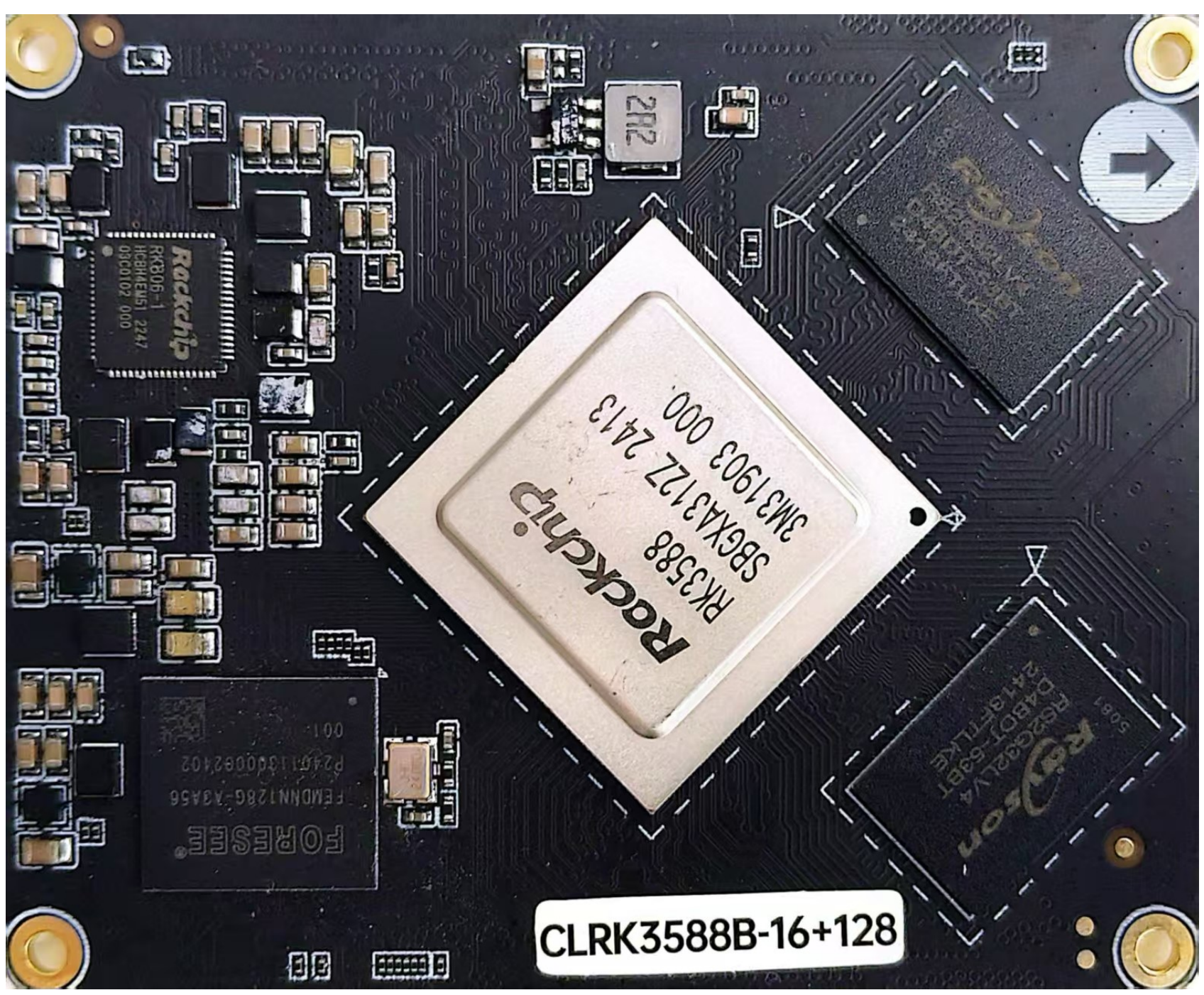

Component	Specification
CPU	4× Cortex-A76 (2.4 GHz) + 4× Cortex-A55 (1.8 GHz)
GPU	Mali-G610 MP4, up to 800 MHz
NPU	6 TOPS (AI inference acceleration)
Memory	8 GB LPDDR4/4X
Storage	32 GB eMMC / UFS 2.1
Power Consumption	Typical 10 W, Max 15 W

Figure 13: Schematic layout and specifications of the RK3588 in-vehicle hardware platform. The module integrates CPU, GPU, NPU, and memory units for efficient on-board deep learning inference.

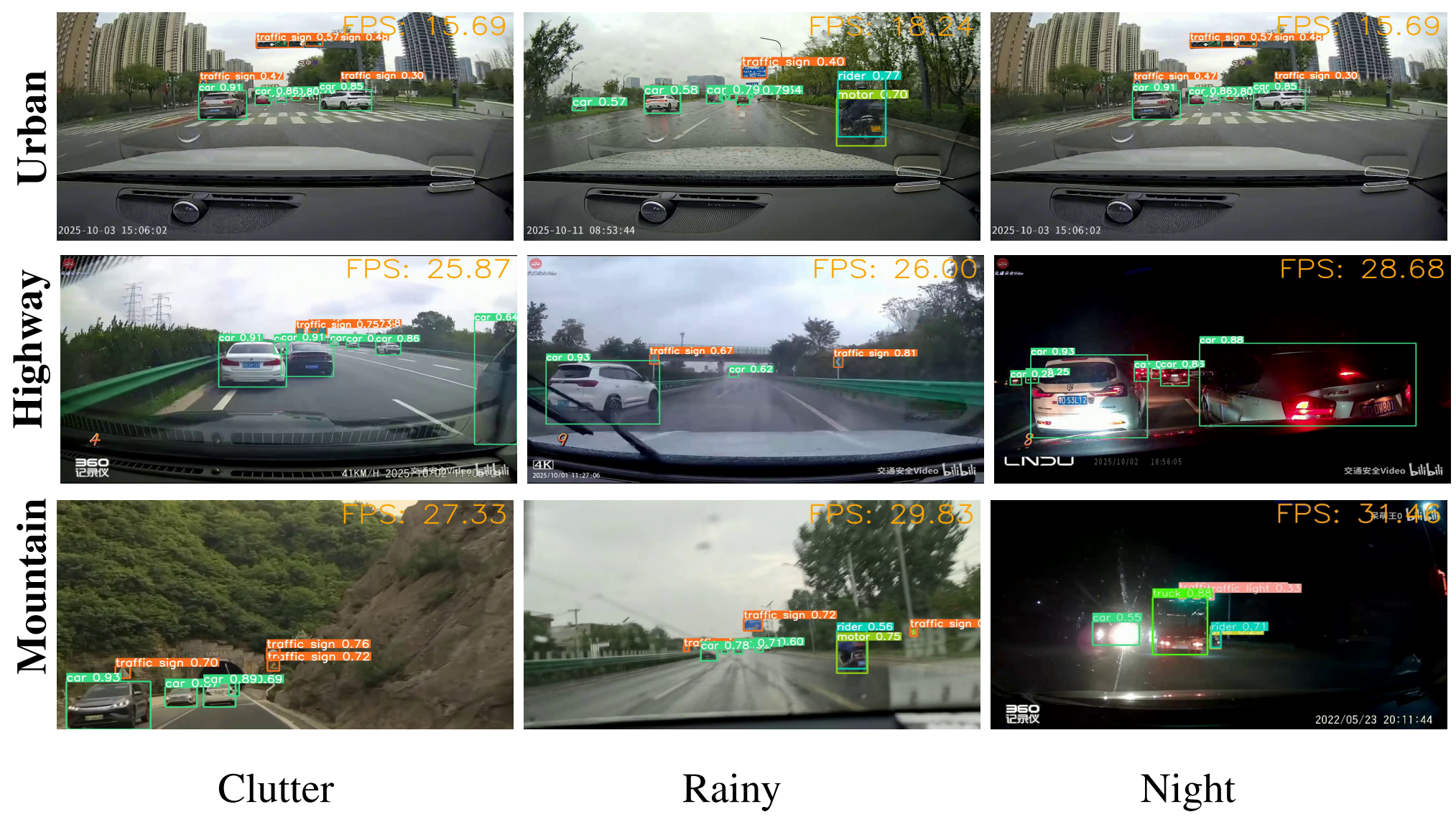

Figure 14: Representative real-time traffic scene detection results of FAD on the RK3588 in-vehicle computing platform, with real-time FPS visualized in the top-right corner.

Page 5

Regarding the reported performance gains (1–3% mAP), we emphasize that the improvement is particularly pronounced for small-object detection and under complex environmental conditions such as low contrast and motion blur. These enhancements are not attributable merely to hyper-parameter tuning, but to the proposed frequency-domain feature extraction and adaptive fusion strategy, which jointly enhance the detector's spatial–spectral representation capability. Importantly, this work bridges frequency-domain feature modeling with real-time edge deployment for traffic scene perception — a novel and meaningful contribution that extends beyond incremental optimization.

We sincerely hope that these revisions and newly added experiments adequately address the

reviewer's concerns. For convenience, the corresponding revised content is provided in the green box below, with page numbers indicated in the lower-right corner. All modifications are also highlighted in purple in the revised manuscript.

Back to main

Response to Reviewer 4

Comments

The paper proposes a new object detector, called Frequency-oriented Adaptive Detector (FAD), designed for traffic scenes in autonomous driving. The key idea is to exploit frequency-domain information to improve detection of small, occluded, and noisy objects in cluttered environments. The method combines two main contributions: Frequency Dynamic Convolution (FDC), which adaptively adjusts dilation rates and kernel weights to balance receptive field and frequency bandwidth, and Adaptive Frequency-oriented Fusion (AFF), which reorganizes high- and low-frequency features across multiple scales to improve consistency and boundary precision. Experiments on KITTI, BDD100K, and Cityscapes show that FAD achieves state-of-the-art accuracy with very small model size (8.77M parameters), demonstrating both efficiency and robustness across datasets.

The main strengths of the paper are the originality of combining frequency-domain analysis with convolution and feature fusion, and the strong empirical performance across the three aforementioned benchmarks. The introduction of FDC is well-motivated, as it provides a principled way to avoid the frequency loss typically caused by dilated convolution. The experimental section is well made: ablations show the incremental contribution of each component, comparisons demonstrate superiority over recent lightweight and heavy detectors. Another strength is the efficiency of the model: despite introducing new modules, the parameter size remains small and inference speed is high, which is crucial for deployment on automotive hardware.

On the other hand, the paper has some weaknesses. First, while the proposed modules are conceptually novel, the technical description is a bit complex, also with many hand-crafted design choices (e.g., decomposition of frequency bands, the use of adaptive filters and offsets) that may limit reproducibility and generalization. The reliance on Fourier-domain interpretations is also not always fully justified. Second, the comparison with state-of-the-art detectors does not always ensure fairness: some baselines are outdated (e.g., SSD, YOLOv3) or not tuned to the same lightweight constraints, while very recent strong baselines (e.g., YOLOv9 or 10, transformer-based detectors) are missing. Third, although the model is lightweight, the reported FLOPs (39.7G) are relatively high compared to other detectors, raising doubts about deployment feasibility on resource-constrained embedded systems.

In summary, the paper addresses an important problem with an original frequency-based perspective, shows strong empirical results and efficiency, but suffers from presentation complexity, incomplete comparisons, and some over-claims.

Reply: Thank you for your insightful comments on our manuscript. We sincerely appreciate the time and effort you have devoted to providing valuable feedback and constructive suggestions. In response to your comments, we have revised the manuscript accordingly. A point-by-point

response is provided below, detailing the modifications made. We hope that our revisions address your concerns and meet your expectations. Thank you once again for your kind review~

Since the revisions are extensive, we summarize them step by step for clarity. We truly appreciate your valuable comments, which have significantly improved our manuscript. All changes are highlighted in purple in the revised version, and the corresponding updated content is presented in the green boxes below with page numbers indicated.

1. While the proposed modules are conceptually novel, the technical description is a bit complex, also with many hand-crafted design choices (e.g., decomposition of frequency bands, the use of adaptive filters and offsets) that may limit reproducibility and generalization. The reliance on Fourier-domain interpretations is also not always fully justified.

Reply:

We appreciate the reviewer's thoughtful comments. In the revision, we have clarified the technical motivation and mathematical formulation of each module, and we will also release the source code to enhance transparency and reproducibility.

The decomposition of frequency bands and the design of adaptive filters are now presented as data-driven processes rather than fixed, hand-crafted operations. We further provide ablation studies to verify the stability of these design choices across different datasets, demonstrating consistent performance gains and strong generalization.

In addition, regarding the motivation for the effectiveness of the Fourier-domain interpretation in visual tasks, we have expanded the theoretical justification and provided intuitive explanations that connect frequency representations to spatial feature enhancement. These revisions collectively improve conceptual clarity and further support the theoretical soundness of our approach.

Revised Content in the Manuscript

1 Introduction

Vision-based traffic scene perception has emerged as a key research frontier in intelligent transportation systems, enabling the detection, recognition, and tracking of diverse road participants. Accurate object detection forms the foundation of reliable scene understanding, serving as a critical component for downstream tasks such as autonomous navigation and traffic management. However, vehicle-mounted traffic object detection (TOD) remains a challenging task due to several inherent difficulties, including scale variation induced by perspective changes, frequent occlusions, diverse lighting and weather conditions, and the limited performance of lightweight detection networks when handling low-quality or small-scale targets. Representative examples of these complexities are illustrated in Fig. 1, drawn from the KITTI [1], BDD100K [2], and Cityscapes [3] datasets.

Deep learning–based object detection algorithms can be broadly categorized into two main

paradigms: two-stage and one-stage detectors. Two-stage approaches, typified by Faster R-CNN [4], first generate region proposals and then perform classification and localization, offering high accuracy but at the expense of computational efficiency. In contrast, one-stage methods such as SSD [5], YOLO [6, 7, 8, 9, 10, 11], and RetinaNet [12] treat detection as a direct regression problem within a single network, achieving a more favorable trade-off between speed and precision. Nonetheless, deploying these models on embedded vehicle platforms remains challenging due to constraints on GPU capacity and memory resources, which limit the practicality of computation-heavy detectors in real-world applications.

In pursuit of a balance between performance and computational efficiency on vehicle-mounted platforms, a variety of model compression strategies and enhanced network architectures have been developed to improve detection efficiency. In terms of feature extraction, recent studies have highlighted the crucial role of a large receptive field in scene understanding tasks [13, 14], yet expanding it inevitably increases computational complexity. To mitigate this trade-off, several works have explored content-adaptive mechanisms [15, 16, 17, 18, 19, 20], which dynamically adjust the convolutional process to input variations. Vision transformers [14, 21, 22, 23] further extend this paradigm by employing self-attention to model long-range dependencies, but their high computational burden limits their deployment on embedded systems. Alternatively, dilated convolution enlarges the receptive field by inserting intervals between filter elements at a predefined dilation rate D, achieving broader contextual perception with low computational overhead, though at the expense of responsiveness to high-frequency components [24]. Meanwhile, deformable convolutions [15, 25, 26] introduce asymmetric spatial offsets of $K \times K \times 2$ to adaptively capture geometric transformations across diverse visual tasks. Yet, in object detection—where precise localization is crucial—maintaining feature consistency remains a significant challenge.

Notably, in autonomous driving scenes, environmental noise predominantly appears as low-frequency interference in the frequency domain [27, 28, 29], whereas the structural and textural details of traffic targets are mainly concentrated in high-frequency components. Leveraging this spectral distinction, frequency-domain feature extraction can effectively suppress background noise while enhancing the representation of salient targets. Frequency-domain analysis has long been a cornerstone of signal processing [30, 31] and has recently been incorporated into deep learning frameworks to model non-local dependencies [32, 33, 34, 35, 36] and improve domain generalization [37]. According to the scaling law of the Fourier Transform [38, 39], increasing the dilation rate from 1 to D proportionally reduces the frequency response bandwidth by $\frac{1}{D}$, thereby diminishing the model's sensitivity to high-frequency details. This observation motivates the integration of frequency-domain principles into convolutional network design to enhance multi-frequency feature representation [40].

Building upon these insights, we propose **Frequency Dynamic Convolution (FDC)**, a novel convolutional operator designed to extract object features from frequency-domain noise in complex traffic scenes, eliminate spatial deviations, and enhance feature consistency for position-sensitive regression.

FDC refines conventional dilated convolution through spectral analysis, introducing a unified single-value dilation rate that reduces redundant coordinate sampling inherent in standard convolutions. Its frequency-guided dilation strategy alleviates the heuristic and non-interpretable nature of empirically defined dilation parameters. By dynamically adjusting the dilation rate in a spatially adaptive manner, FDC optimizes kernel bandwidth utilization and learns frequency-balancing functions that promote broader receptive fields, thereby achieving a principled equilibrium between effective bandwidth and receptive field coverage.

Moreover, autonomous driving scenes pose substantial challenges, including background noise, illumination variation, motion blur, and multi-scale traffic objects induced by perspective effects, thereby necessitating the extraction of features with strong category semantics and precise spatial boundaries. To address these challenges, feature fusion [72] has been widely adopted in various architectures [73, 74, 75, 76] to integrate high-level semantic features with low-level high-resolution details. In general, high-level features provide rich categorical context, whereas low-level features preserve fine-grained spatial details. However, upsampling coarse features via nearest-neighbor or bilinear interpolation and merging them with high-resolution features often leads to *intra-category inconsistency* and *boundary displacement* [40]. The former arises from variations within the same object, such as differences in texture and brightness between a vehicle's wheel and window [77], while the latter is caused by excessive smoothing introduced during interpolation [78, 79]. Conventional fusion methods [75] are unable to effectively address these issues and may even exacerbate them by diffusing a single feature across multiple pixels. To quantify these phenomena, feature similarity analysis is employed, as illustrated in Fig. 2. Intra-category inconsistency is evaluated through *intra-category similarity*, which measures the similarity between each feature vector and its corresponding category center [80]. Likewise, *inter-category similarity* is computed to assess the similarity margin between different classes. Boundary displacement is characterized by low intra-category similarity and small similarity margins in boundary regions. Since classification scores depend on the similarity between category-aware weights and feature representations [81], features with reduced intra-category similarity and margin ultimately yield lower classification confidence, leading to potential misclassifications.

Therefore, rapid variations in feature values within traffic objects, corresponding to disturbed high-frequency components, lead to low intra-category similarity and feature inconsistency [82]. Additionally, blurred boundaries lack precise high-frequency information, resulting in boundary displacement. Frequency-domain processing is essential for maintaining intra-category consistency and precise object boundaries, as it enables effective preservation and adaptive utilization of high-frequency features. To leverage frequency-domain information for improved feature fusion, we propose an adaptive frequency-oriented fusion (AFF) framework. AFF consists of four key components: Low-pass Adaptive Filter (LAF) generator, offset generator, High-pass Adaptive Filter (HAF) generator, and Cross-scale Contribution Allocator (CCA).

Specifically, the LAF applies a low-pass filter to smooth and upsample coarse high-level features, re-

ducing disparities between pixel values and mitigating feature inconsistency. LAF alone is insufficient to correct large-area inconsistencies or refine boundaries. To address this limitation, we introduce an offset generator. The offset generator first calculates local similarity and then predicts offsets toward regions of high similarity for resampling, effectively replacing features with low intra-category similarity. It rectifies inconsistent features across both large regions and narrow boundary areas. Restoring fine boundary details lost during downsampling of low-level features into high-level representations remains challenging. According to the Nyquist-Shannon sampling theorem [83, 84], frequencies exceeding the Nyquist threshold, defined as half the sampling rate, are irretrievably lost. To address this limitation, we introduce the HAF generator, which applies spatially adaptive high-pass filters to low-level features, extracting intricate boundary details and enhancing high-frequency energy beyond the Nyquist limit. During bottom-up feature integration, the CCA progressively fuses high-level features with low-level features from non-adjacent scales, reducing the semantic gap between disparate scales. Through the coordinated operation of these four components, AFF reconstructs fused features that preserve consistent category information and well-defined object boundaries in complex traffic scenarios.

Our proposed frequency-oriented adaptive detector (FAD) integrates Frequency Dynamic Convolution (FDC) for frequency-guided feature extraction with Adaptive Frequency-oriented Fusion (AFF) for frequency-guided adaptive feature integration. By explicitly capturing high- and low-frequency components, FAD strengthens both the textural and semantic features of objects across scales. By explicitly leveraging high- and low-frequency components, FAD enhances multi-scale object detection in complex traffic environments while preserving inference efficiency.

2.1 Frequency Domain Learning

Frequency-domain analysis has long been established as a fundamental technique in traditional signal processing [30, 31], and recent studies have increasingly adopted it within deep learning contexts to enhance model efficiency and representation capacity, particularly for visual tasks where spatial structures can be effectively decomposed and analyzed in the spectral domain. Rahaman *et al.* [85] and Xu *et al.* [86] identify a tendency of deeper hidden layers to favor lower-frequency components during training, resulting in a learning bias toward low-frequency modes, a phenomenon referred to as the spectral bias or frequency principle. Zhang *et al.* [87] examine the impact of frequency aliasing on the shift-invariance of contemporary models, and AdaBlur [88] addresses this issue by incorporating content-aware low-pass filters during downsampling to mitigate aliasing effects. Similarly, FLC [89] demonstrates that frequency aliasing can substantially compromise model robustness. Qin *et al.* [90] and Magid *et al.* [91] incorporate additional frequency components derived from discrete cosine transform (DCT) coefficients into channel attention mechanisms, thereby improving feature representation. Huang *et al.* [34] apply the classical convolution theorem to develop adaptive frequency filters that function as global token mixers within DNNs. Furthermore, a range of frequency-domain techniques is integrated into network architectures to facilitate the modeling of non-local dependencies [34, 32, 36, 35, 92]. Chen *et al.* [93] employ low-pass filters to suppress high-frequency noise introduced by image degradation,

effectively enhancing the performance of instance segmentation under low-light conditions. In parallel, numerous studies demonstrate that adversarial attacks can be realized by manipulating high-frequency components [82, 94, 95]. For example, Luo *et al.* [82] show that perturbations in the high-frequency domain significantly reduce intra-category similarity, thereby deteriorating the quality of learned representations. Therefore, our proposed approach introduces a frequency-domain interpretation of dilated convolution to better motivate and strengthen its effectiveness for visual perception tasks, enhancing its capacity to capture features across diverse frequency components. In addition, intra-category inconsistency and boundary displacement are attributed to the absence of high-frequency content, aligning with findings in [91]. To address these challenges, adaptive low-pass filters are employed to suppress inconsistent high-frequency noise, while high-pass filters are utilized to preserve task-relevant high-frequency details and to sharpen boundaries, ultimately improving the fidelity and discriminative power of visual representations.

2. The comparison with state-of-the-art detectors does not always ensure fairness: some baselines are outdated (e.g., SSD, YOLOv3) or not tuned to the same lightweight constraints, while very recent strong baselines (e.g., YOLOv9 or 10, transformer-based detectors) are missing.

Reply:

We appreciate the reviewer's thoughtful comment regarding the fairness of baseline comparisons. Our initial choice of detectors (e.g., SSD, YOLOv3) was made because several recent studies have adopted these models as standard baselines for evaluating lightweight detection performance.

To further ensure fairness and contemporaneity, we have additionally compared our method with several recent state-of-the-art detectors, including YOLOv8, YOLOv10, YOLOv11, and RT-DETR, all trained and evaluated under consistent lightweight settings. The updated results in Table 4 demonstrate that our method continues to outperform these advanced baselines, confirming its robustness and superiority across both classical and modern detection frameworks.

Revised Content in the Manuscript

Table 3: Detection Performance Comparison with Related Works on the KITTI Validation Set Across Classes and Difficulties.

Method	mAP	Car			Pedestrian			Cyclist			FPS
		Easy	Mod.	Hard	Easy	Mod.	Hard	Easy	Mod.	Hard	
5-Ensemble[45]	73.70	97.84	89.75	80.87	77.08	67.66	59.17	79.45	57.30	54.17	5.10
CertainNet[46]	73.00	93.81	89.36	82.11	76.33	66.13	58.54	78.02	57.49	55.20	16.46
Centernet[47]	71.49	90.30	89.15	82.17	76.53	67.53	59.37	73.48	52.63	50.25	20.21
SSD[48]	60.79	87.34	87.74	77.27	50.38	48.41	43.46	48.25	52.31	52.13	33.17
ASSD[49]	75.28	89.28	89.95	82.11	69.07	62.49	60.18	75.23	76.16	72.83	33.04
SA-YOLO[50]	83.82	91.71	87.16	80.97	86.49	80.63	76.91	87.28	83.44	79.83	32.68
YOLOv8[41]	89.31	98.32	98.50	90.54	90.63	87.65	80.03	89.26	87.01	79.39	69.04
YOLOv10[43]	88.80	98.58	98.60	90.53	90.74	86.69	86.91	89.28	87.04	79.21	66.33
YOLOv11[44]	88.40	98.46	98.37	90.44	90.45	85.00	87.96	87.98	85.76	78.21	85.49
**FAD**	**90.90**	98.55	**98.91**	**90.63**	89.85	**88.16**	**88.13**	**90.84**	**90.33**	**89.71**	57.60

Table 4: Quantitative comparisons of the different methods on KITTI, BDD100K and Cityscapes datasets. Our proposed method achieves the best metrical scores across the three datasets.

Method	KITTI			Method	BDD100K			Method	Cityscapes		
	mAP	Params	GFLOPS		mAP	FPS	Params		mAP	Params	GFLOPS
SA-YOLO[50]	81.8	–	–	CertainNet[46]	40.9	–	–	MDNet[61]	37.0	15.11	**12.3**
YOLOv3[7]	82.2	234.94	65.3	SA-YOLO[50]	17.3	48.5	–	DAYOLO[64]	49.3	9.35	19.7
MobileSSD[51]	71.8	33.86	12.5	CenterNet[47]	44.5	46.0	93.50	PDEM[65]	43.3	–	26.7
EdgeYOLO[52]	82.1	24.48	**9.9**	YOLOv7[10]	44.3	71.0	42.00	UMS^2-ODNet[66]	48.3	34.9	85.9
BHF-MTM[53]	88.6	35.80	–	PASFLN[58]	46.3	40.0	117.00	Faster R-CNN[67]	40.8	41.5	180.5
FD-Head[54]	90.5	–	23.2	SDA-OD[59]	42.8	33.0	–	YOLOv7[10]	33.5	36.9	55.5
YOLOX[11]	89.0	9.00	26.7	OFFR-YOLO[60]	45.3	-	-	PWNet[68]	49.1	–	–
YOLOv8[55]	89.3	11.20	28.7	MDNet[61]	45.0	46.0	14.80	CPSS-FAT[69]	39.4	48.4	181.1
SHW[56]	86.8	8.30	22.9	SpikeTOD[62]	43.7	–	–	AugFCOS[70]	42.3	–	–
PIAENet[57]	83.4	17.90	3.2	CSIM[63]	50.8	–	–	IRG[71]	47.7	–	–
YOLOv8[41]	89.3	11.40	29.7	YOLOv8[41]	51.0	69.8	29.7	YOLOv8[41]	47.9	11.40	29.7
YOLOv10[43]	88.8	9.17	24.8	YOLOv10[43]	51.8	67.3	24.8	YOLOv10[43]	47.3	9.17	24.8
YOLOv11[44]	88.4	9.40	21.5	YOLOv11[44]	51.5	80.7	21.5	YOLOv11[44]	47.8	9.40	21.5
RT-DETR[42]	88.5	42.70	136.1	RT-DETR[42]	51.6	45.1	136.1	RT-DETR[42]	48.5	42.70	136.1
**Ours**	**90.9**	**8.77**	39.7	**Ours**	**53.7**	55.6	**8.77**	**Ours**	**50.2**	**8.77**	39.7

4.4 Comparison Experiments and Quantitative Analysis

1) Results on KITTI: We compare our method with state-of-the-art approaches to the KITTI validation criteria, and detailed results are shown in Table 3. FAD achieves the highest mAP of 90.90%, outperforming all competitors. This highlights the effectiveness of our proposed method. Additionally, our approach excels in 2 specific categories: car and cyclist. Notably, FAD achieves an FPS of 57.60, demonstrating the balance between performance and computational resources achieved by our approach.

As shown in Table 4, our method surpasses the mainstream YOLO series and RT-DETR in mAP. Notably, it achieves the highest mAP while maintaining exceptional efficiency, with only 8.77M parameters—making it the most lightweight model among all compared approaches.

2) Results on BDD100K: We conducted a comprehensive evaluation of FAD on the BDD100K dataset, benchmarking it against the latest advancements in the field. FAD notably outperforms its

counterparts, achieving a remarkable mAP of over 53.7% (as displayed in Table 4). Our method showcases exceptional generalization capabilities, demonstrating outstanding performance even on larger traffic datasets. Notably, with an input image size of 640×640, FAD surpasses all existing detectors, boasting an impressive FPS of 55.6 while maintaining the smallest model size among its peers.

Moreover, our achieved mAP of 53.7% represents a substantial 1.9% improvement over the runner-up, YOLOv11. Additionally, the visualization results presented in Fig. 11 vividly demonstrates FAD's precision in detecting objects across diverse scales and aspect ratios, effectively handling complex backgrounds, occlusions, low-quality objects, dense scenes, nighttime conditions, and small objects.

3) Results on Cityscapes: As shown in Table 4, FAC attains 50.2% mAP on the Cityscapes dataset, exceeding most existing lightweight detectors, including the latest YOLO series and RT-DETR. Such performance is particularly remarkable given the dataset's emphasis on densely structured urban environments with frequent occlusions and abundant small objects. These results highlight FAC's enhanced ability to perceive fine-grained details and maintain detection stability under complex visual conditions, underscoring its robustness in real-world traffic scenarios.

Table 5: Quantitative comparisons of the different methods on Waymo 2D datasets.

Method	mAP@50	mAP@50-95	Vehichle	Person	Cyclist
YOLOv8[41]	51.0	32.7	64.5	56.2	32.4
RT-DETR[42]	52.3	32.6	65.1	56.7	32.6
YOLOv10[43]	51.8	32.9	65.5	57.0	33.1
YOLOv11[44]	51.0	32.5	64.6	56.6	31.8
FAD	**53.1**	**33.0**	**68.0**	**59.7**	31.7

4) Results on Waymo 2D: As shown in Table 5, FAC attains 50.2% mAP on the Cityscapes dataset, exceeding most existing lightweight detectors, including the latest YOLO series and RT-DETR. Such performance is particularly remarkable given the dataset's emphasis on densely structured urban environments with frequent occlusions and abundant small objects. These results highlight FAC's enhanced ability to perceive fine-grained details and maintain detection stability under complex visual conditions, underscoring its robustness in real-world traffic scenarios.

3. Although the model is lightweight, the reported FLOPs (39.7G) are relatively high compared to other detectors, raising doubts about deployment feasibility on resource-constrained embedded systems.

Reply: We sincerely thank the reviewer for this insightful comment. It has significantly contributed to enhancing the clarity and quality of our manuscript. The experiments in this study were conducted on an RTX 4070 Ti workstation to ensure adequate computational resources for model training and evaluation. We acknowledge that this platform may not reflect the constraints typically

found in automotive-grade hardware environments.

To address this concern, we have included additional experiments and analysis on an in-vehicle computing platform RK3588 in Section 4.6. This allows us to assess the real-time performance and deployment feasibility of our proposed model under automotive-grade hardware conditions. We have also highlighted this distinction and the experimental setup in the revised manuscript. Furthermore, a real-time demonstration video has been provided as supplementary material. The results from these tests show that FAD maintains real-time inference capabilities while achieving a favorable trade-off between accuracy and efficiency in real-world edge computing environments.

To ensure clarity regarding the changes, we have outlined the revisions in detail below:

Revised Content in the Manuscript

4.1 Implementation Specifications

The network is trained under the following configuration. Mosaic data augmentation is applied with a stochastic probability of 0.5. Training is performed using the Stochastic Gradient Descent (SGD) optimizer with a momentum of 0.9, an initial learning rate of 0.01, and cosine annealing to decay the learning rate to 0.0001. Input images are resized to 640×640 pixels and drawn from four datasets, with a batch size of 8. All experiments are conducted in the PyTorch framework on a Windows workstation equipped with an RTX 4070 Ti 12-GB GPU and 32-GB RAM. To further evaluate deployment feasibility, inference is additionally performed on an in-vehicle computing platform RK3588, verifying the deployment suitability and runtime efficiency of the model on an automotive-grade platform.

Page 5

Revised Content in the Manuscript

4.6 In-Vehicle Computing Platform Experiments

The in-vehicle computing platform utilized in this study is built upon the **Rockchip RK3588** system-on-chip, which offers high-performance heterogeneous computing and multimedia processing capabilities optimized for real-time visual perception and deep learning inference in autonomous driving scenarios. The schematic layout and hardware specifications of the platform are jointly presented in Fig. 12.

To evaluate the practical performance of the proposed FAD algorithm in dynamic driving conditions, we deployed the inference pipeline primarily on the RK3588's NPU, while the CPU and GPU handled pre-processing and auxiliary computations. The model was configured with an input resolution of 640×640 and converted into the RKCNN format to fully exploit the NPU's parallel acceleration capability. Leveraging hardware-level NPU inference substantially reduced computational latency and ensured stable real-time performance. FAD was initialized using pre-trained weights on the BDD100K dataset to ensure robust generalization and performance in real-world traffic scenarios. Experimental results indi-

cate that FAD achieves an average inference speed of 35.14 FPS at full input resolution, with an average per-frame latency of 28.5 ms, ensuring real-time processing suitable for in-vehicle deployment. FAD maintains high detection accuracy across varying traffic densities and lighting conditions, with negligible impact on mAP. These results highlight the capability of the RK3588's heterogeneous computing architecture to efficiently manage computational load and energy consumption, making it well-suited for the real-time deployment of advanced perception algorithms such as FAD in autonomous driving scenarios.

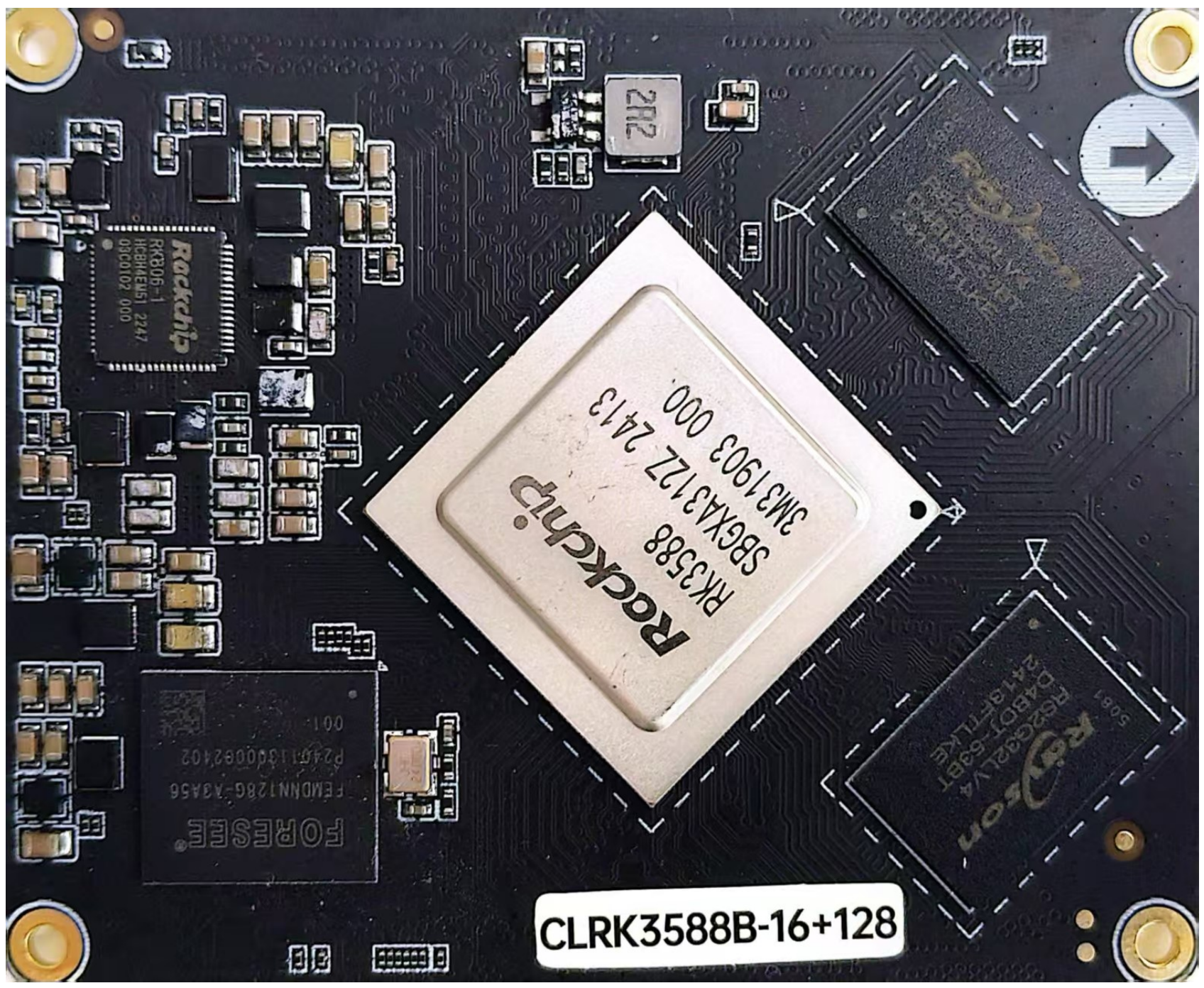

Component	Specification
CPU	4× Cortex-A76 (2.4 GHz) + 4× Cortex-A55 (1.8 GHz)
GPU	Mali-G610 MP4, up to 800 MHz
NPU	6 TOPS (AI inference acceleration)
Memory	8 GB LPDDR4/4X
Storage	32 GB eMMC / UFS 2.1
Power Consumption	Typical 10 W, Max 15 W

Figure 13: Schematic layout and specifications of the RK3588 in-vehicle hardware platform. The module integrates CPU, GPU, NPU, and memory units for efficient on-board deep learning inference.

Urban

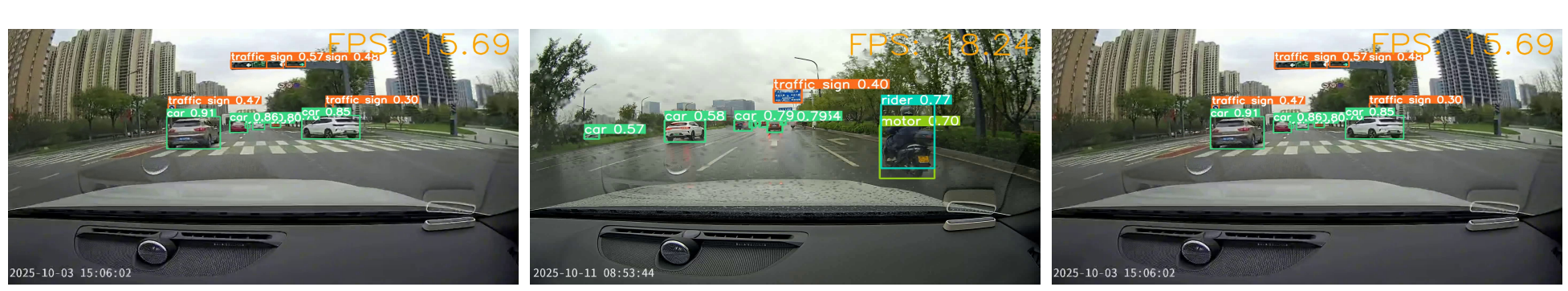

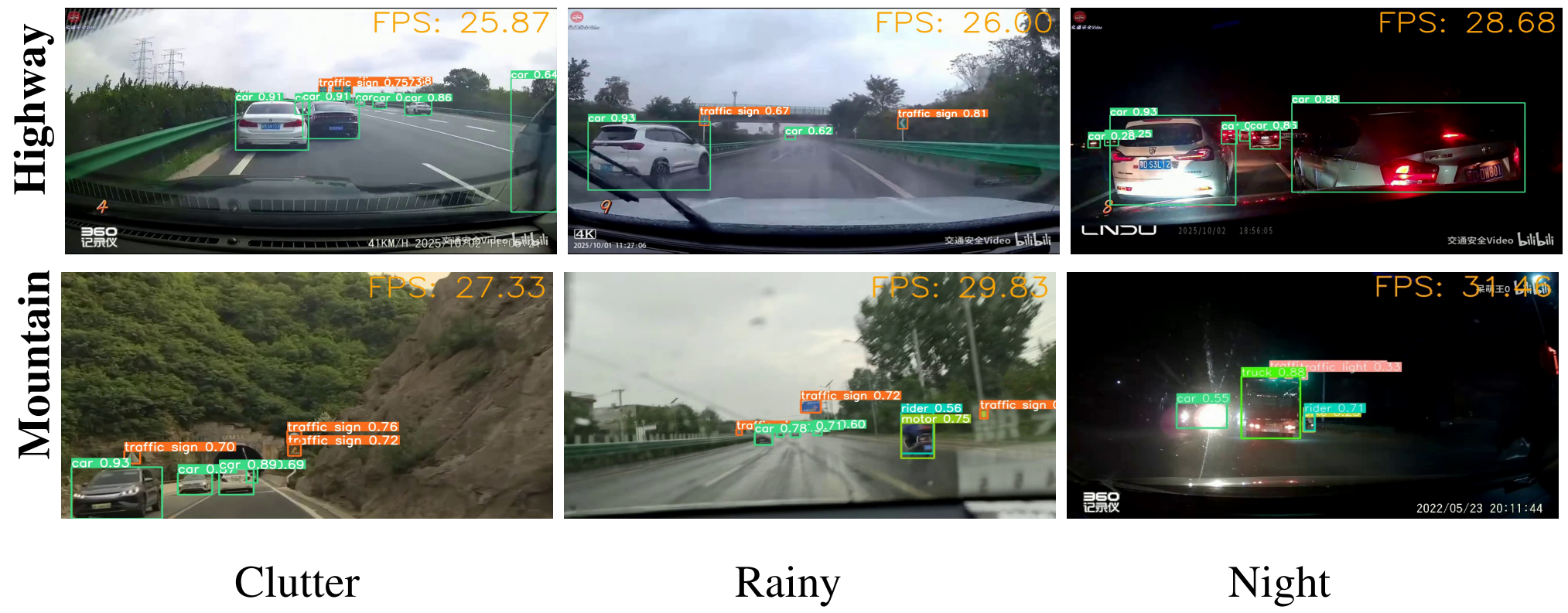

Figure 14: Representative real-time traffic scene detection results of FAD on the RK3588 in-vehicle computing platform, with real-time FPS visualized in the top-right corner.

Page 5

4. In summary, the paper addresses an important problem with an original frequency-based perspective, shows strong empirical results and efficiency, but suffers from presentation complexity, incomplete comparisons, and some over-claims.

Reply:

We thank the reviewer for their thoughtful summary of our paper. We appreciate the recognition of the originality and empirical strength of our work. We also fully acknowledge the concerns raised regarding presentation complexity, incomplete comparisons, and over-claims.

To address these issues, we have made the following revisions in the manuscript:

1. Presentation Complexity: We have reorganized certain sections to improve clarity and readability, particularly in the explanation of our frequency-based approach. We have streamlined the technical details and provided additional visual aids, including figures and tables, to make the presentation more intuitive. A new section dedicated to summarizing the key contributions and insights of our work has also been added to guide the reader through the core findings.

2. Incomplete Comparisons: In response to the comment about incomplete comparisons, we have expanded the experimental evaluation to include a broader set of baseline methods, covering both traditional and recent approaches in the field. This comparison now provides a more comprehensive assessment of our method's performance, including results on additional datasets and across different conditions. We have also updated the revised manuscript to explicitly discuss these comparisons and the improvements our method brings over existing approaches.

3. Over-claims: We appreciate the reviewer pointing out the over-claims. We have carefully revised the manuscript to ensure that all claims are precise and substantiated by the experimental results. In particular, we have clarified the scope of our method's advantages and have been more

cautious in the interpretation of certain results, ensuring they are presented within the appropriate context.

We hope these revisions address the concerns raised, and we believe they have significantly improved the overall presentation and robustness of our paper.

Back to main

At the End

At the conclusion of this response, we would like to express our sincere gratitude to the journal editors and the four reviewers for their invaluable support and guidance throughout this process. We also extend our heartfelt blessings: As autumn unfolds with its golden hues, may your research thrive and yield abundant results in this season of harvest. May the clarity and crispness of the season bring renewed inspiration and strength to your endeavors. We wish you continued success in your work, and may each day be filled with joy, fulfillment, and productivity. May your academic pursuits flourish, and your efforts be met with great success.

Thank you again for your time ~

References

[1] Geiger, A., Lenz, P. & Urtasun, R. Are we ready for autonomous driving? the kitti vision benchmark suite. In *2012 IEEE conference on computer vision and pattern recognition*, 3354–3361 (IEEE, 2012).

[2] Yu, F. *et al.* Bdd100k: A diverse driving dataset for heterogeneous multitask learning. In *2020 IEEE/CVF Conference on Computer Vision and Pattern Recognition (CVPR)*, 2633–2642 (2020).

[3] Cordts, M. *et al.* The cityscapes dataset for semantic urban scene understanding. In *Proceedings of the IEEE conference on computer vision and pattern recognition*, 3213–3223 (2016).

[4] Ren, S., He, K., Girshick, R. & Sun, J. Faster r-cnn: Towards real-time object detection with region proposal networks. *Advances in neural information processing systems* **28** (2015).

[5] Agrawal, N. *et al.* Design tradeoffs for {SSD} performance. In *2008 USENIX Annual Technical Conference (USENIX ATC 08)* (2008).

[6] Redmon, J., Divvala, S., Girshick, R. & Farhadi, A. You only look once: Unified, real-time object detection. In *Proceedings of the IEEE conference on computer vision and pattern recognition*, 779–788 (2016).

[7] Redmon, J. & Farhadi, A. Yolov3: An incremental improvement. *arXiv preprint arXiv:1804.02767* (2018).

[8] Wang, C.-Y., Bochkovskiy, A. & Liao, H.-Y. M. Scaled-yolov4: Scaling cross stage partial network. In *Proceedings of the IEEE/cvf conference on computer vision and pattern recognition*, 13029–13038 (2021).

[9] Zhu, X., Lyu, S., Wang, X. & Zhao, Q. Tph-yolov5: Improved yolov5 based on transformer prediction head for object detection on drone-captured scenarios. In *Proceedings of the IEEE/CVF international conference on computer vision*, 2778–2788 (2021).

[10] Wang, C.-Y., Bochkovskiy, A. & Liao, H.-Y. M. Yolov7: Trainable bag-of-freebies sets new state-of-the-art for real-time object detectors. In *Proceedings of the IEEE/CVF Conference on Computer Vision and Pattern Recognition*, 7464–7475 (2023).

[11] Ge, Z., Liu, S., Wang, F., Li, Z. & Sun, J. Yolox: Exceeding yolo series in 2021. *arXiv preprint arXiv:2107.08430* (2021).

[12] Lin, T.-Y., Goyal, P., Girshick, R., He, K. & Dollár, P. Focal loss for dense object detection. In *Proceedings of the IEEE international conference on computer vision*, 2980–2988 (2017).

[13] Ding, X., Zhang, X., Han, J. & Ding, G. Scaling up your kernels to 31x31: Revisiting large kernel design in cnns. In *Proceedings of the IEEE/CVF conference on computer vision and pattern recognition*, 11963–11975 (2022).

[14] Alexey, D. An image is worth 16x16 words: Transformers for image recognition at scale. *arXiv preprint arXiv: 2010.11929* (2020).

[15] Dai, J. *et al.* Deformable convolutional networks. In *Proceedings of the IEEE international conference on computer vision*, 764–773 (2017).

[16] Han, Q. *et al.* On the connection between local attention and dynamic depth-wise convolution. *arXiv preprint arXiv:2106.04263* (2021).

[17] Romero, D. W. *et al.* Flexconv: Continuous kernel convolutions with differentiable kernel sizes. *arXiv preprint arXiv:2110.08059* (2021).

[18] Su, H. *et al.* Pixel-adaptive convolutional neural networks. In *Proceedings of the IEEE/CVF Conference on Computer Vision and Pattern Recognition*, 11166–11175 (2019).

[19] Verelst, T. & Tuytelaars, T. Dynamic convolutions: Exploiting spatial sparsity for faster inference. In *Proceedings of the ieee/cvf conference on computer vision and pattern recognition*, 2320–2329 (2020).

[20] Zhou, J., Jampani, V., Pi, Z., Liu, Q. & Yang, M.-H. Decoupled dynamic filter networks. In *Proceedings of the IEEE/CVF Conference on Computer Vision and Pattern Recognition*, 6647–6656 (2021).

[21] Hassani, A., Walton, S., Li, J., Li, S. & Shi, H. Neighborhood attention transformer. In *Proceedings of the IEEE/CVF Conference on Computer Vision and Pattern Recognition*, 6185–6194 (2023).

[22] Liu, Z. *et al.* Swin transformer: Hierarchical vision transformer using shifted windows. In *Proceedings of the IEEE/CVF international conference on computer vision*, 10012–10022 (2021).

[23] Moorthy, S., KS, S. S., Arthanari, S., Jeong, J. H. & Joo, Y. H. Hybrid multi-attention transformer for robust video object detection. *Engineering Applications of Artificial Intelligence* **139**, 109606 (2025).

[24] Yu, F., Koltun, V. & Funkhouser, T. Dilated residual networks. In *Proceedings of the IEEE conference on computer vision and pattern recognition*, 472–480 (2017).

[25] Wang, W. *et al.* Internimage: Exploring large-scale vision foundation models with deformable convolutions. In *Proceedings of the IEEE/CVF conference on computer vision and pattern recognition*, 14408–14419 (2023).

[26] Zhu, X., Hu, H., Lin, S. & Dai, J. Deformable convnets v2: More deformable, better results. In *Proceedings of the IEEE/CVF conference on computer vision and pattern recognition*, 9308–9316 (2019).

[27] Moorthy, S. & Joo, Y. H. Learning dynamic spatial-temporal regularized correlation filter tracking with response deviation suppression via multi-feature fusion. *Neural Networks* **167**, 360–379 (2023).

[28] Moorthy, S., KS, S. S., Arthanari, S., Jeong, J. H. & Joo, Y. H. Learning disruptor-suppressed response variation-aware multi-regularized correlation filter for visual tracking. *Journal of Visual Communication and Image Representation* 104458 (2025).

[29] Arthanari, S., Moorthy, S., Jeong, J. H. & Joo, Y. H. Adaptive spatially regularized target attribute-aware background suppressed deep correlation filter for object tracking. *Signal Processing: Image Communication* **136**, 117305 (2025).

[30] Baxes, G. A. *Digital image processing: principles and applications* (John Wiley & Sons, Inc., 1994).

[31] Pitas, I. *Digital image processing algorithms and applications* (John Wiley & Sons, 2000).

[32] Chi, L., Jiang, B. & Mu, Y. Fast fourier convolution. *Advances in Neural Information Processing Systems* **33**, 4479–4488 (2020).

[33] Guibas, J. *et al.* Adaptive fourier neural operators: Efficient token mixers for transformers. *arXiv preprint arXiv:2111.13587* (2021).

[34] Huang, Z. *et al.* Adaptive frequency filters as efficient global token mixers. In *Proceedings of the IEEE/CVF International Conference on Computer Vision*, 6049–6059 (2023).

[35] Li, Z. *et al.* Fourier neural operator for parametric partial differential equations. *arXiv preprint arXiv:2010.08895* (2020).

[36] Rao, Y., Zhao, W., Zhu, Z., Lu, J. & Zhou, J. Global filter networks for image classification. *Advances in neural information processing systems* **34**, 980–993 (2021).

[37] Lin, S. *et al.* Deep frequency filtering for domain generalization. In *Proceedings of the IEEE/CVF conference on computer vision and pattern recognition*, 11797–11807 (2023).

[38] Proakis, J. G. & Manolakis, D. G. *Digital signal processing: Pearson new international edition* (Pearson Higher Ed, 2013).

[39] Roberts, R. A. & Mullis, C. T. *Digital signal processing* (Addison-Wesley Longman Publishing Co., Inc., 1987).

[40] Chen, L. *et al.* Frequency-aware feature fusion for dense image prediction. *IEEE Transactions on Pattern Analysis and Machine Intelligence* (2024).

[41] Zhang, H., Liang, M. & Wang, Y. Yolo-bs: a traffic sign detection algorithm based on yolov8. *Scientific Reports* **15**, 7558 (2025).

[42] Lv, W. *et al.* Rt-detrv2: Improved baseline with bag-of-freebies for real-time detection transformer. *arXiv preprint arXiv:2407.17140* (2024).

[43] Wang, A. *et al.* Yolov10: Real-time end-to-end object detection. *Advances in Neural Information Processing Systems* **37**, 107984–108011 (2024).

[44] Khanam, R. & Hussain, M. Yolov11: An overview of the key architectural enhancements. *arXiv preprint arXiv:2410.17725* (2024).

[45] Miller, D., Dayoub, F., Milford, M. & Sünderhauf, N. Evaluating merging strategies for sampling-based uncertainty techniques in object detection. In *2019 international conference on robotics and automation (icra)*, 2348–2354 (IEEE, 2019).

[46] Gasperini, S. *et al.* Certainnet: Sampling-free uncertainty estimation for object detection. *IEEE Robotics and Automation Letters* **7**, 698–705 (2022).

[47] Duan, K. *et al.* Centernet: Keypoint triplets for object detection. In *Proceedings of the IEEE/CVF international conference on computer vision*, 6569–6578 (2019).

[48] Liu, W. *et al.* Ssd: Single shot multibox detector. In *Computer Vision–ECCV 2016: 14th European Conference, Amsterdam, The Netherlands, October 11–14, 2016, Proceedings, Part I 14*, 21–37 (Springer, 2016).

[49] Yi, J., Wu, P. & Metaxas, D. N. Assd: Attentive single shot multibox detector. *Computer Vision and Image Understanding* **189**, 102827 (2019).

[50] Tian, D. *et al.* Sa-yolov3: An efficient and accurate object detector using self-attention mechanism for autonomous driving. *IEEE Transactions on Intelligent Transportation Systems* **23**, 4099–4110 (2022).

[51] Biswas, D., Su, H., Wang, C., Stevanovic, A. & Wang, W. An automatic traffic density estimation using single shot detection (ssd) and mobilenet-ssd. *Physics and Chemistry of the Earth, Parts A/B/C* **110**, 176–184 (2019).

[52] Liang, S. *et al.* Edge yolo: Real-time intelligent object detection system based on edge-cloud cooperation in autonomous vehicles. *IEEE Transactions on Intelligent Transportation Systems* **23**, 25345–25360 (2022).

[53] Zhang, Y. *et al.* Parallel attention for multi-task road object detection in autonomous driving. *IEEE Sensors Journal* (2024).

[54] Hua, J. *et al.* Re-decoupling the classification branch in object detectors for few-class scenes. *Pattern Recognition* **153**, 110541 (2024).

[55] Varghese, R. & Sambath, M. Yolov8: A novel object detection algorithm with enhanced performance and robustness. In *2024 International Conference on Advances in Data Engineering and Intelligent Computing Systems (ADICS)*, 1–6 (IEEE, 2024).

[56] Jiang, Y. *et al.* Strong-help-weak: An online multi-task inference learning approach for robust advanced driver assistance systems. *IEEE Transactions on Intelligent Transportation Systems* (2024).

[57] Tang, X. *et al.* Piaenet: pyramid integration and attention enhanced network for object detection. *Information Sciences* **670**, 120576 (2024).

[58] Yin, Y., Kou, T., Ochieng, N. D. & Bao, X. Pasfln: Positional association and semantic fusion learning network for traffic object detection. In *2023 IEEE 26th International Conference on Intelligent Transportation Systems (ITSC)*, 329–334 (2023).

[59] Li, G., Ji, Z. & Qu, X. Stepwise domain adaptation (sda) for object detection in autonomous vehicles using an adaptive centernet. *IEEE Transactions on Intelligent Transportation Systems* **23**, 17729–17743 (2022).

[60] Tian, D., Han, Y. & Wang, S. Object feedback and feature information retention for small object detection in intelligent transportation scenes. *Expert Systems with Applications* **238**, 121811 (2024).

[61] Guo, X. *et al.* Deep learning-enhanced environment perception for autonomous driving: Mdnet with csp-darknet53. *Pattern Recognition* **160**, 111174 (2025).

[62] Wang, J. *et al.* Spiketod: A biologically interpretable spike-driven object detection in challenging traffic scenarios. *IEEE Transactions on Intelligent Transportation Systems* (2024).

[63] Lu, Y.-F. *et al.* A cross-scale and illumination invariance-based model for robust object detection in traffic surveillance scenarios. *IEEE transactions on intelligent transportation systems* **24**, 6989–6999 (2023).

[64] Li, G., Ji, Z., Qu, X., Zhou, R. & Cao, D. Cross-domain object detection for autonomous driving: A stepwise domain adaptative yolo approach. *IEEE Transactions on Intelligent Vehicles* **7**, 603–615 (2022).

[65] Lin, C., Shi, Y., Xie, C. & Li, M. Quantification and mitigation of border-level localization deviation for object detectors. *Expert Systems with Applications* 127435 (2025).

[66] Li, Y., Zhang, Y., Yang, C. & Chen, Y. Ums2-odnet: Unified-scale domain adaptation mechanism driven object detection network with multi-scale attention. *Neural Networks* **181**, 106890 (2025).

[67] Girshick, R. Fast r-cnn. In *Proceedings of the IEEE international conference on computer vision*, 1440–1448 (2015).

[68] Zhang, Z., Gong, H., Feng, Y., Chu, Z. & Liu, H. Enhancing object detection in adverse weather conditions through entropy and guided multimodal fusion. In Cho, M., Laptev, I., Tran, D., Yao, A. & Zha, H. (eds.) *Computer Vision – ACCV 2024*, 22–38 (Springer Nature Singapore, Singapore, 2025).

[69] Yang, X. *et al.* Cpss-fat: A consistent positive sample selection for object detection with full adaptive threshold. *Pattern Recognition* **141**, 109627 (2023).

[70] Zhang, X. *et al.* Augfcos: Augmented fully convolutional one-stage object detection network. *Pattern Recognition* **134**, 109098 (2023).

[71] VS, V., Oza, P. & Patel, V. M. Instance relation graph guided source-free domain adaptive object detection. In *2023 IEEE/CVF Conference on Computer Vision and Pattern Recognition (CVPR)*, 3520–3530 (2023).

[72] Zhang, Z., Zhang, X., Peng, C., Xue, X. & Sun, J. Exfuse: Enhancing feature fusion for semantic segmentation. In *Proceedings of the European conference on computer vision (ECCV)*, 269–284 (2018).

[73] Long, J., Shelhamer, E. & Darrell, T. Fully convolutional networks for semantic segmentation. In *Proceedings of the IEEE conference on computer vision and pattern recognition*, 3431–3440 (2015).

[74] Falk, T. *et al.* U-net: deep learning for cell counting, detection, and morphometry. *Nature methods* **16**, 67–70 (2019).

[75] Lin, T.-Y. *et al.* Feature pyramid networks for object detection. In *Proceedings of the IEEE conference on computer vision and pattern recognition*, 2117–2125 (2017).

[76] Kirillov, A., Girshick, R., He, K. & Dollár, P. Panoptic feature pyramid networks. In *Proceedings of the IEEE/CVF conference on computer vision and pattern recognition*, 6399–6408 (2019).

[77] Yu, C. *et al.* Learning a discriminative feature network for semantic segmentation. In *Proceedings of the IEEE conference on computer vision and pattern recognition*, 1857–1866 (2018).

[78] Lu, H. *et al.* Sapa: Similarity-aware point affiliation for feature upsampling. *Advances in Neural Information Processing Systems* **35**, 20889–20901 (2022).

[79] Lu, H., Liu, W., Fu, H. & Cao, Z. Fade: Fusing the assets of decoder and encoder for task-agnostic upsampling. In *European Conference on Computer Vision*, 231–247 (Springer, 2022).

[80] Wu, D. *et al.* Semantic segmentation via pixel-to-center similarity calculation. *CAAI Transactions on Intelligence Technology* **9**, 87–100 (2024).

[81] Chang, S., Wang, P., Luo, H., Wang, F. & Shou, M. Z. Revisiting vision transformer from the view of path ensemble. In *Proceedings of the IEEE/CVF International Conference on Computer Vision*, 19889–19899 (2023).

[82] Luo, C. *et al.* Frequency-driven imperceptible adversarial attack on semantic similarity. In *Proceedings of the IEEE/CVF conference on computer vision and pattern recognition*, 15315–15324 (2022).

[83] Shannon, C. E. Communication in the presence of noise. *Proceedings of the IRE* **37**, 10–21 (1949).

[84] Nyquist, H. Certain topics in telegraph transmission theory. *Proceedings of the IEEE* **90**, 280–305 (2002).

[85] Rahaman, N. *et al.* On the spectral bias of neural networks. In *International conference on machine learning*, 5301–5310 (PMLR, 2019).

[86] Xu, Z. J. & Zhou, H. Deep frequency principle towards understanding why deeper learning is faster. In *Proceedings of the AAAI conference on artificial intelligence*, vol. 35, 10541–10550 (2021).

[87] Zhang, R. Making convolutional networks shift-invariant again. In *International conference on machine learning*, 7324–7334 (PMLR, 2019).

[88] Zou, X., Xiao, F., Yu, Z., Li, Y. & Lee, Y. J. Delving deeper into anti-aliasing in convnets. *International Journal of Computer Vision* **131**, 67–81 (2023).

[89] Grabinski, J., Jung, S., Keuper, J. & Keuper, M. Frequencylowcut pooling-plug and play against catastrophic overfitting. In *European Conference on Computer Vision*, 36–57 (Springer, 2022).

[90] Qin, Z., Zhang, P., Wu, F. & Li, X. Fcanet: Frequency channel attention networks. In *Proceedings of the IEEE/CVF international conference on computer vision*, 783–792 (2021).

[91] Magid, S. A. *et al.* Dynamic high-pass filtering and multi-spectral attention for image super-resolution. In *Proceedings of the IEEE/CVF International Conference on Computer Vision*, 4288–4297 (2021).

[92] Guibas, J. *et al.* Adaptive fourier neural operators: Efficient token mixers for transformers, 2021. *URL https://arxiv. org/abs/2111.13587* .

[93] Chen, L., Fu, Y., Wei, K., Zheng, D. & Heide, F. Instance segmentation in the dark. *International Journal of Computer Vision* **131**, 2198–2218 (2023).

[94] Yin, D., Gontijo Lopes, R., Shlens, J., Cubuk, E. D. & Gilmer, J. A fourier perspective on model robustness in computer vision. *Advances in Neural Information Processing Systems* **32** (2019).

[95] Jia, S. *et al.* Exploring frequency adversarial attacks for face forgery detection. In *Proceedings of the IEEE/CVF conference on computer vision and pattern recognition*, 4103–4112 (2022).

Detailed Response to Reviewers

Thank you for your insightful comments on our manuscript. We sincerely appreciate the time and effort you have devoted to providing valuable feedback and constructive suggestions. In response to your comments, we have revised the manuscript accordingly. A point-by-point response is provided below, detailing the modifications made. We hope that our revisions address your concerns and meet your expectations.

Thank you once again for your kind review~

Response to Reviewer 3

Comments

I would like to thank the authors for their detailed and comprehensive response to my previous comments. I sincerely appreciate the significant effort put into the revision, particularly the inclusion of additional experiments on the large-scale Waymo 2D dataset and the deployment validation on the RK3588 in-vehicle platform. The inclusion of recent state-of-the-art baselines, such as YOLOv10, YOLOv11, and RT-DETR, has significantly strengthened the fairness and contemporaneity of the performance evaluation. The experimental validation is now much more robust and demonstrates the practical effectiveness of the FAD framework in real-world scenarios.

However, despite these improvements, there are still critical issues that need to be addressed before I can fully recommend this manuscript for publication in Nature Communications.

1. Statistical Significance Analysis (Unresolved): In my initial review, I explicitly requested a statistical significance analysis to ensure that the reported improvements (1-3% mAP) are statistically meaningful and not merely the result of stochastic variance. While the revised manuscript and response emphasize "consistent performance" across datasets, formal statistical tests (e.g., t-tests, p-values, or confidence intervals across multiple runs) are still missing. Given that the performance gains are relatively marginal in some cases, providing statistical evidence is non-negotiable for a high-impact journal to confirm the reliability of the results. Please include this analysis in the final version.

2. Novelty and Theoretical Contribution: I appreciate the authors' honesty in acknowledging that the proposed modules (frequency-domain analysis, dilated convolution, etc.) conceptually align with prior works. The authors argue that the novelty lies in the "reinterpretation and integration" of these concepts through a frequency-based perspective. While I agree that the engineering integration is executed well and the results are solid, my concern regarding the work being an "incremental improvement" remains. To meet the high novelty bar of Nature Communications, the paper needs to elevate itself beyond a successful "application-driven design". I strongly suggest the authors strengthen the theoretical explanation of why this frequency-oriented approach is superior. Rather than just describing how it works (reinterpretation), please provide deeper theoretical insights or mathematical justifications explaining why the frequency domain is the optimal space for solving the specific problems of feature alignment and boundary precision in this context.

I am satisfied with the experimental breadth and the engineering robustness of the revised manuscript. However, to support the claims of superiority and justify publication in this venue, it is essential to: Add the missing statistical significance analysis. And deepen the theoretical discussion to demonstrate a more fundamental contribution beyond engineering integration.

I look forward to seeing these final polishings.

Reply: We sincerely thank the reviewer for the careful re-evaluation of our manuscript and for the constructive and insightful feedback. We greatly appreciate the reviewer's recognition of the substantial improvements in the revised version, particularly the expanded large-scale evaluation on the Waymo 2D dataset, the real-world deployment on the RK3588 in-vehicle platform, and the inclusion of recent state-of-the-art detectors (e.g., YOLOv10, YOLOv11, and RT-DETR), which have significantly strengthened the fairness and practical relevance of our study.

We also appreciate the reviewer's continued efforts in identifying the remaining critical issues. Following these suggestions, we have further strengthened the manuscript in two key aspects:

(1) *Statistical significance analysis*: We fully agree that rigorous statistical validation is essential for confirming the reliability of the reported improvements. In the revised manuscript, we provide statistical significance analysis based on multiple independent runs, including confidence intervals and hypothesis testing results, to demonstrate that the observed performance gains are statistically meaningful rather than caused by stochastic variation.

(2) *Theoretical understanding of the frequency-oriented design*: We acknowledge the reviewer's concern regarding the level of novelty and have substantially strengthened the theoretical discussion. Specifically, we provide deeper insights into why the frequency domain offers a more suitable representation for feature alignment and boundary-sensitive perception, supported by additional analysis and mathematical interpretation, which elevates the contribution beyond empirical integration.

We believe these revisions further consolidate both the reliability and the theoretical grounding of our method. Detailed point-by-point responses are provided below.

Thank you once again for your kind review~

1. Statistical significance analysis.

Reply: Thank you for your constructive suggestions. Your feedback has been extremely helpful in improving the rigor and clarity of our manuscript. We sincerely apologize for the insufficient rigor and clarity in the original version. In the revised manuscript, we have added a rigorous statistical significance analysis based on five independent runs with different random seeds, including the mean performance, standard deviation, 95% confidence interval, and paired t-test results.

For clarity, we summarize the revisions step by step below. We truly appreciate your valuable comments, which have significantly improved the quality of our work. All changes are highlighted in purple in the revised manuscript, and the corresponding updated content is presented in the green boxes below with the relevant page numbers indicated.

Revised Content in the Manuscript

5) Statistical Reliability Analysis: To verify that the observed improvements are not caused by stochastic variation, we conduct statistical analysis on KITTI over five independent runs with different random seeds.

As shown in Table 6, our method achieves a mean mAP of 90.5 with a standard deviation of 0.25, indicating stable performance across runs, while the best result reaches 90.9 mAP. The 95% confidence interval (90.3–90.7) further characterizes the uncertainty of the estimated mean. In comparison, YOLOv11 (88.1–88.4) and RT-DETR (88.1–88.5) exhibit non-overlapping confidence intervals, indicating a clear performance margin. Two-sided paired t-tests further confirm the statistical significance of the improvements. Specifically, our method achieves gains of +2.3 and +2.2 mAP over YOLOv11 and RT-DETR, with p-values of 0.003 and 0.001, respectively. Statistical significance is confirmed, and the observed improvements cannot be attributed to stochastic variation.

Table 6: Statistical significance analysis on KITTI (5 runs, paired t-test).

Method	Mean mAP (%)	Best (%)	Std	95% CI
YOLOv11	88.2	88.4	0.16	88.1–88.4
RT-DETR	88.3	88.5	0.19	88.1–88.5
**Ours**	**90.5**	**90.9**	**0.25**	**90.3–90.7**
Significance (vs. Ours)				
YOLOv11	+2.3			**p = 0.003**
RT-DETR	+2.2			**p = 0.001**

Page 22-23

2. Theoretical understanding of the frequency-oriented design.

Reply:

We sincerely thank the reviewer for this insightful suggestion. We agree that, in the previous revision, our presentation mainly emphasized how the proposed modules work, while the deeper reason for why frequency-oriented modeling is particularly suitable for this problem was not made sufficiently explicit.

Our core motivation is that the two main failure modes in cluttered traffic object detection—feature misalignment across scales and imprecise object boundaries—can be fundamentally characterized in the frequency domain. Semantic consistency is mainly supported by low-/mid-frequency structures, whereas boundary localization and fine object details depend on preserving discriminative high-frequency components. In this sense, the frequency domain is not merely a reinterpretation tool, but a physically grounded space in which these two requirements can be explicitly separated

and jointly controlled. More specifically, our theoretical rationale is three-fold.

(1) For receptive-field enlargement, dilation introduces a deterministic frequency trade-off: according to the Fourier scaling property, increasing dilation compresses the kernel's effective frequency response and weakens sensitivity to high-frequency details. Therefore, adaptive dilation in FDC is designed to balance contextual coverage and bandwidth preservation, rather than serving as a purely empirical modification.

(2) For cross-scale fusion, conventional upsampling and downsampling alter the spectrum in predictable ways: smoothing suppresses boundary-related high-frequency content, while downsampling causes loss or aliasing of frequencies beyond the Nyquist limit. This directly explains why naive fusion often leads to intra-category inconsistency and boundary displacement.

(3) For accurate detection, not all high-frequency components should be treated equally. Some correspond to discriminative boundaries, while others reflect noise or inconsistent local textures. Hence, the key is not indiscriminate high-frequency enhancement, but adaptive frequency reallocation: low-pass filtering improves intra-category consistency, high-pass filtering restores missing boundary cues, and similarity-guided resampling corrects spatial misalignment.

These observations collectively indicate that the difficulty of jointly achieving semantic consistency and boundary precision originates from a frequency imbalance introduced by multi-scale operations. By explicitly modeling and reallocating frequency components, our approach transforms this coupled spatial problem into a structured and analyzable spectral optimization problem.

Therefore, the frequency domain provides a theoretically grounded and more suitable representation for jointly modeling semantic consistency and boundary precision, rather than a heuristic reinterpretation of existing modules.

Based on this suggestion, we will revise the manuscript to make this theoretical line more explicit and add a dedicated explanation clarifying that the superiority of our frequency-oriented design comes from its ability to transform the coupled spatial problem of "semantic consistency vs. boundary precision" into an analyzable and optimizable spectral trade-off.

Revised Content in the Manuscript

1 Introduction

Notably, in autonomous driving scenarios, environmental noise predominantly manifests as low-frequency interference in the frequency domain [1, 2, 3], whereas the structural and textural details of traffic objects are primarily concentrated in high-frequency components. Exploiting this spectral distinction, frequency-domain feature extraction enables the suppression of background noise while enhancing the representation of salient objects. Frequency analysis, a long-standing foundation of signal processing [4, 5], has recently been incorporated into deep learning frameworks to model non-local de-

pendencies [6, 7, 8, 9, 10] and to improve domain generalization [11]. According to the scaling property of the Fourier Transform [12, 13], increasing the dilation rate from 1 to D proportionally narrows the frequency response bandwidth by a factor of $\frac{1}{D}$, thereby reducing the model's sensitivity to high-frequency details. This insight motivates the incorporation of frequency-domain principles into convolutional network design to enhance multi-frequency feature representation [14]. From a spectral perspective, feature alignment and boundary precision are governed by distinct frequency components: low-frequency structures encode semantic consistency, while high-frequency components capture boundary details. However, multi-scale operations introduce frequency-dependent distortions that disrupt this balance, leading to feature inconsistency and boundary displacement. Consequently, the underlying challenge is a spectral imbalance, for which frequency-domain modeling provides a natural and principled solution.

Page 3

Revised Content in the Manuscript

1 Introduction

Therefore, rapid variations in feature values within traffic objects, corresponding to disturbed high-frequency components, lead to low intra-category similarity and feature inconsistency [15]. Feature inconsistency and boundary degradation arise from the imbalance of frequency components across spatial locations, where insufficient high-frequency information further results in imprecise boundary localization. This highlights the necessity of frequency-aware modeling for preserving feature consistency and boundary precision. Accordingly, we propose an adaptive frequency-oriented fusion (AFF) framework to regulate the allocation of frequency components during multi-scale feature integration. AFF consists of four key components: Low-pass Adaptive Filter (LAF) generator, offset generator, High-pass Adaptive Filter (HAF) generator, and Cross-scale Contribution Allocator (CCA).

Page 4

Revised Content in the Manuscript

3.1 Frequency Dynamic Convolution

An overview of the proposed FDC is provided in Fig. 3. Initially, we present the frequency selection strategy for balancing bandwidth and receptive field, followed by a discussion of adaptive dilation rates and kernels that jointly enhance bandwidth utilization and receptive field size FDC is designed to address the bandwidth–receptive field trade-off caused by dilation-induced spectral compression, thereby preserving high-frequency sensitivity while enlarging contextual coverage.

Page 9

Revised Content in the Manuscript

3.2 Adaptive Frequency-oriented Fusion

In this section, we present Adaptive Frequency-oriented Fusion (AFF) framework, as depicted in Fig. 5. AFF comprises four key components: the low-pass adaptive filter generator (LAF), the offset generator, the high-pass adaptive filter generator (HAF), and cross-scale contribution allocation (CCA). AFF is designed to mitigate the spectral imbalance introduced during multi-scale feature fusion by adaptively reallocating low- and high-frequency components for feature consistency and boundary precision.

Page 13

At the End

At the conclusion of this response, we would like to express our sincere gratitude to the journal editors and Reviewer 3 for their invaluable support and guidance throughout this process. As spring arrives with renewed vitality, we wish you continued success in your research and academic endeavors. May this season of growth and renewal bring fresh inspiration, clarity, and momentum to your work. We hope your efforts flourish and lead to meaningful and rewarding outcomes.

Thank you again for your time ~

References

[1] Moorthy, S. & Joo, Y. H. Learning dynamic spatial-temporal regularized correlation filter tracking with response deviation suppression via multi-feature fusion. *Neural Networks* **167**, 360–379 (2023).

[2] Moorthy, S., KS, S. S., Arthanari, S., Jeong, J. H. & Joo, Y. H. Learning disruptor-suppressed response variation-aware multi-regularized correlation filter for visual tracking. *Journal of Visual Communication and Image Representation* 104458 (2025).

[3] Arthanari, S., Moorthy, S., Jeong, J. H. & Joo, Y. H. Adaptive spatially regularized target attribute-aware background suppressed deep correlation filter for object tracking. *Signal Processing: Image Communication* **136**, 117305 (2025).

[4] Baxes, G. A. *Digital image processing: principles and applications* (John Wiley & Sons, Inc., 1994).

[5] Pitas, I. *Digital image processing algorithms and applications* (John Wiley & Sons, 2000).

[6] Chi, L., Jiang, B. & Mu, Y. Fast fourier convolution. *Advances in Neural Information Processing Systems* **33**, 4479–4488 (2020).

[7] Guibas, J. *et al.* Adaptive fourier neural operators: Efficient token mixers for transformers. *arXiv preprint arXiv:2111.13587* (2021).

[8] Huang, Z. *et al.* Adaptive frequency filters as efficient global token mixers. In *Proceedings of the IEEE/CVF International Conference on Computer Vision*, 6049–6059 (2023).

[9] Li, Z. *et al.* Fourier neural operator for parametric partial differential equations. *arXiv preprint arXiv:2010.08895* (2020).

[10] Rao, Y., Zhao, W., Zhu, Z., Lu, J. & Zhou, J. Global filter networks for image classification. *Advances in neural information processing systems* **34**, 980–993 (2021).

[11] Lin, S. *et al.* Deep frequency filtering for domain generalization. In *Proceedings of the IEEE/CVF conference on computer vision and pattern recognition*, 11797–11807 (2023).

[12] Proakis, J. G. & Manolakis, D. G. *Digital signal processing: Pearson new international edition* (Pearson Higher Ed, 2013).

[13] Roberts, R. A. & Mullis, C. T. *Digital signal processing* (Addison-Wesley Longman Publishing Co., Inc., 1987).

[14] Chen, L. *et al.* Frequency-aware feature fusion for dense image prediction. *IEEE Transactions on Pattern Analysis and Machine Intelligence* (2024).

[15] Luo, C. *et al.* Frequency-driven imperceptible adversarial attack on semantic similarity. In *Proceedings of the IEEE/CVF conference on computer vision and pattern recognition*, 15315–15324 (2022).
